# Effects of during-exercise oral hydration strategies on endurance performance and thermophysiological strain: a systematic review with meta-analysis

**DOI:** 10.3389/fnut.2026.1968824

**Published:** 2026-10-27

**Authors:** Xiaojie Zhang, Ling Wang, Ziyu Liu

**Affiliations:** 1 Hebei Sport University, Shijiazhuang, China; 2 Shijiazhuang Institute of Railway Technology, Shijiazhuang, China; 3 Northeast Electric Power University, Jilin, China

**Keywords:** endurance performance, exercise, fluid replacement, hydration, meta-analysis, sodium, systematic review, thermoregulation

## Abstract

**Background:**

The effects of during-exercise drinking may differ by strategy and outcome construct. Methods: Six databases were searched from database inception to 1 June 2026 for randomized trials in healthy adults completing at least 60 min of continuous endurance exercise. Two reviewers independently screened records, extracted data, and assessed risk of bias and certainty. Construct-specific random-effects meta-analyses used restricted maximum likelihood and modified Hartung–Knapp intervals.

**Results:**

Twenty-four studies were retained (225 study-reported participants: 170 men, 20 women, 35 with sex unreported); 21 contributed to pairwise meta-analysis. Oral hydration versus no or minimal fluid showed no clear time-trial/fixed-duration benefit (−0.1%, 95% CI − 3.6 to 3.5%; four studies). The time-to-exhaustion estimate favored hydration (34.1%, 11.9–60.7%; three studies, 21 participants), but its prediction interval included no benefit (−21.4 to 128.8%). Core temperature (−0.38 °C, −0.74 to −0.02; nine studies) and heart rate (−6.82 beats/min, −11.16 to −2.48; 12 studies) favored hydration on average, with high heterogeneity and prediction intervals crossing the null. Programmed/individualized versus self-regulated drinking did not clearly improve performance (0.7%, −4.4 to 6.2%) but reduced measured body-mass loss (−1.47 percentage points, −1.96 to −0.99), an expected consequence of greater intake. Volume-matched sodium evidence was sparse; plasma-volume change was imprecise (1.78 percentage points, −5.24 to 8.81; two studies). All critical/important estimates were very low certainty.

**Conclusion:**

Physiological and exhaustion-test averages favored hydration, but self-paced performance and optimal fluid or sodium dosing remain uncertain. Small samples, incomplete source coverage and *post hoc* analytical decisions limit inference.

**Systematic review registration:**

https://www.crd.york.ac.uk/PROSPERO/view/CRD420261460972.

## Introduction

Prolonged endurance exercise can produce substantial sweat losses, especially in warm or humid environments. Progressive body-water deficit reduces plasma volume, increases cardiovascular drift and body heat storage, and can affect perceived effort and exercise capacity; the size of these responses depends on environmental heat load, exercise intensity and duration, acclimatization, and the magnitude and rate of fluid loss (1–6).

Hydration practice is therefore not a choice between a single correct volume and complete fluid restriction. Sweat rate and sweat sodium concentration vary markedly between and within athletes, while gastrointestinal tolerance and access to fluid constrain what can be consumed. Planned drinking can be useful when sweat losses are predictable or dehydration carries particular risk, whereas drinking in response to thirst may be adequate in many settings. Excessive intake is also harmful because overdrinking can cause weight gain and contribute to exercise-associated hyponatremia (7–9).

The performance literature remains contested. Meta-analyses have reported context-dependent effects of dehydration and fluid ingestion, but estimates are sensitive to whether exercise is conducted in laboratory or field conditions and whether the test is a self-paced time trial, fixed-duration work task, or open-ended time-to-exhaustion test. These constructs differ in ecological validity, pacing control, and susceptibility to expectancy and motivational effects, so a mixed performance estimate can obscure clinically important differences (10–14).

Fluid composition and drinking pattern add further complexity. Beverage tonicity, electrolyte content and energy density influence gastric emptying, fluid delivery and plasma-volume responses; carbohydrate content and tonicity therefore cannot be considered independently (7, 15). Higher intake or sodium exposure does not necessarily improve performance and may increase gastrointestinal burden. Exercise-induced gastrointestinal disturbances are multifactorial and can be modified by heat, splanchnic hypoperfusion, feeding, hydration volume and prior gut training (15–17).

This review asked: among healthy adults performing at least 60 min of continuous endurance exercise, what are the effects of during-exercise oral hydration strategies, compared with another strategy within the same randomized trial, on endurance performance and thermophysiological strain? We synthesized direct head-to-head comparisons separately for drinking regimen, fluid-replacement dose and sodium/electrolyte dose. “Pairwise” describes an analytical contrast, not a restriction to two-arm studies: eligible arms from multi-condition trials could contribute. Fluid balance, perceived exertion, gastrointestinal tolerance and supporting safety outcomes were also examined.

## Methods

### Reporting framework and review status

Reporting followed PRISMA 2020 and PRISMA-S (18, 19). The review was registered in PROSPERO (CRD420261460972; 15 July 2026). The original registration plan proposed additive component network meta-analysis, treatment/component rankings and CINeMA, with GRADE for key pairwise summaries. The final synthesis instead used direct pairwise comparisons because the sparse and heterogeneous component contrasts did not provide a sufficiently secure basis for independent component effects or treatment rankings. No network model, inconsistency test or ranking was performed. A separate full protocol was not prepared.

Supplementary Table S1 compares the available registration plan with the analyses performed; inclusion in that plan does not establish prospective registration. The performance ratio-of-means scale, outcome-selection hierarchy, specific variance assumptions, GRADE decision rules, additional subgroup analyses and within-study dose–response analyses are reported as *post hoc* choices. No timestamped version history of the complete analytical plan was available to verify the prospective status of individual analytical decisions; registration before screening is therefore not asserted. The move from network to pairwise synthesis is a substantive deviation, rather than a change in terminology.

### Eligibility criteria

Eligible studies enrolled healthy adults aged at least 18 years, of either sex and any training status, undertaking continuous endurance exercise such as running or cycling for at least 60 min. The threshold included an endurance preload and subsequent performance test within the same experimental visit, but not separate familiarization or sweat-rate assessment visits. Brief interruptions required for measurements were distinguished from programmed repeated work–rest bouts. Protocols dominated by repeated passive rests, resistance exercise or repeated maximal neuromuscular-power tests were not treated as continuous endurance exercise.

Randomized crossover or parallel-group studies had to compare at least two eligible during-exercise oral strategies. Random allocation had to apply to the hydration contrast itself, not merely to environmental conditions. Eligible contrasts were oral hydration versus no or minimal fluid; programmed or individualized versus self-regulated drinking; higher versus lower fluid-replacement dose; higher versus lower sodium/electrolyte dose with fluid volume and carbohydrate appropriately matched; ad libitum versus thirst-driven drinking; and distributed versus front-loaded timing.

Trials confined to pre-exercise loading or recovery, non-oral fluid delivery, cooling interventions, or unmatched active ingredients were excluded. A carbohydrate-electrolyte beverage versus water at matched volume was not considered an isolated fluid-volume or sodium comparison. The original plan nevertheless allowed a fluid-versus-no-fluid contrast using a carbohydrate-containing beverage when no water-only arm existed; this was retained as a combined drinking strategy, not described as carbohydrate-matched or as an isolated water effect, and examined by exclusion sensitivity analysis. Sodium supplementation under ad libitum drinking was not treated as a volume-matched sodium contrast when achieved fluid intake differed.

### Information sources and search strategy

PubMed, Web of Science Core Collection, Embase.com, Cochrane Library, Scopus and SPORTDiscus were searched from each database’s inception through 1 June 2026. No publication-year or language limit was applied. Search concepts combined endurance exercise, hydration/fluid/electrolyte terms and randomized or crossover designs. Database inception refers to the start of each database’s coverage, not the beginning of this review. The search strategies in Supplementary Table S2 are reconstructed, not authenticated records of the searches originally executed; original execution histories, individual execution dates and database-specific retrieval counts were not retained.

The Cochrane Library search included CENTRAL; however, this does not constitute a separate comprehensive search of ClinicalTrials.gov or the WHO ICTRP. Embase coverage included conference records, but conference abstracts were not eligible primary reports. Dissertations and unpublished or ongoing trials were not searched separately. Reference lists and reviewer-nominated reports were used to identify additional candidates. A reproducible log of a systematic forward-citation search was not available, so complete forward-citation coverage is not claimed. These restrictions and incomplete source-level search documentation may have limited coverage.

### Study selection and data collection

Two reviewers independently screened titles and abstracts, assessed potentially eligible full texts, extracted data using standardized forms, and assessed risk of bias and certainty. Disagreements were resolved by discussion and, when needed, a third reviewer. The reconciled 93-record eligibility log was used for the final selection counts. The re-audit considered randomization of the actual hydration contrast, exercise structure, co-intervention matching and whether each extracted measurement represented an intervention outcome (Supplementary Table S3).

Study-, arm-, comparison- and result-level data captured sample size, sex, training status, exercise duration, environmental conditions, fluid amount and timing, target and achieved replacement, sodium/carbohydrate content, beverage temperature, measurement site, timepoint and numerical precision. Intervention details are provided in Supplementary Table S4. Original authors were not contacted for missing paired data or dispersion; unavailable information was not imputed as observed data. A report retained in the eligibility record but lacking a verifiable complete source was described narratively and given no inverse-variance weight.

### Outcome selection and comparison domains

Time-trial/fixed-duration work and time-to-exhaustion performance were treated as different constructs. Within the former, completion time was preferred, followed by distance, mean power or work, with equivalent speed/pace measures considered only when higher-priority measures were unavailable. An available time-to-exhaustion result was not substituted for a time trial or pooled with it. A protocol-completion duration capped by a scheduled endpoint or safety rule was not classified as either a time trial or an uncensored time-to-exhaustion endpoint.

For core temperature, the documented selection prioritized rectal, then esophageal, then other core measurements; a mixed mean-body-temperature index was not substituted for core temperature. Post-exposure values were preferred to change scores where both were available, and the latest relevant exercise assessment was selected; an end-preload value remained eligible when no later same-outcome value was reported. Baseline-only measurements were not intervention effects. For heart rate, end/fixed-timepoint and exercise-average measurements were distinguished and a restricted analysis examined fixed-timepoint values. Multiple eligible environments or exercise modes were averaged within study. The two highest eligible dose levels defined a higher-versus-lower contrast; the highest eligible level was used against zero or minimal fluid. Supplementary Table S5 documents outcome allocation, including alternative, single-study and narrative evidence, with a record-level audit in Supplementary material 2.

### Effect measures and pairwise meta-analysis

Performance effects were direction-harmonized log ratios of means, back-transformed to percentage differences (20). Positive values favor the first-named strategy: oral hydration, programmed/individualized drinking, higher fluid dose, higher sodium dose, ad libitum instructions or distributed timing. A completion-time ratio was inverted so that shorter time was favorable; distance, power and exhaustion time retained the higher-is-better direction. Physiological and symptom outcomes used mean differences in their original units, calculated as the first-named strategy minus its comparator. Smaller body-mass loss indicates a smaller measured deficit, not an independent physiological benefit. Fluid-intake-corrected mass loss was kept separate from net body-mass deficit.

At least two independent studies were required for pooling. Random-effects models used restricted maximum likelihood and modified Hartung–Knapp intervals, with the variance inflation factor constrained to at least one (21). Heterogeneity was summarized by τ^2^ and I^2^. Prediction intervals used a t distribution with k − 2 degrees of freedom and were reported only for *k* ≥ 3 (22). Different environments, durations and replacement strategies made a common-effect assumption unattractive even for two-study syntheses, but τ^2^ and Hartung–Knapp intervals are particularly unstable at *k* = 2. Such results were retained as imprecise summaries, not firm evidence of benefit. Single-study direct estimates were not meta-analyzed.

### Crossover and multi-condition dependence

Paired precision was preferred to reconstruction from marginal arm dispersions (23). Fallowfield (time to exhaustion) and Sage (time-trial performance) provided paired information from which within-participant correlations of approximately 0.940 and 0.513 were recovered for log-ratio variance propagation. Other author-reported paired mean differences and intervals, including Wijering’s plasma-volume and sodium-change results and Sage’s end-preload measurements, were used directly. When an interval rather than a standard error was available, its width was converted using t(n − 1), an approximation stated in the calculation record.

Where paired precision was unavailable, SDs were recovered from reported SEs using SD=SE × √n, and paired mean-difference variance was (SD₁^2^ + SD₀^2^ − 2rSD₁SD₀)/n with *r* = 0.50. The corresponding delta-method covariance was used for log ratios. Correlations of 0.25 and 0.75 were tested. Multiple condition-specific effects from the same participants were averaged with equal weights, using covariance ρ√(vᵢvj); the primary between-condition correlation was 0.50, with 0 and 0.90 examined. Sample sizes counted participants once within each synthesis, not arms or repeated observations. The calculations and sources are presented in Supplementary Table S6.

### Exploratory dose–response assessment

Within-study generalized least-squares slopes related target sweat-loss replacement to performance, using the zero-replacement condition as the reference and retaining the covariance shared by dose contrasts (24–26). Only Dugas and McConell supplied usable multilevel performance data. Because one assessed a time trial and the other time to exhaustion, their slopes were not pooled. Slopes per 10 percentage points, correlation sensitivity and target-versus-achieved replacement differences were retained as supplementary exploratory evidence; no nonlinear model or optimal dose was estimated.

### Risk of bias and certainty of evidence

RoB 2 judgments were linked to specific results and included crossover period/carryover considerations (27). The weights contributed by low-risk, some-concerns and high-risk results were calculated separately for each synthesis. GRADE was applied to every estimable critical or important comparison–outcome pair, including single-study estimates, but not to purely supporting intervention-manipulation outcomes (28). Detailed judgments are reported in Supplementary Table S7.

The following review-specific rules were applied consistently and were not prospectively registered. Risk of bias was downgraded one level when more than 10% of weight was from some-concerns or high-risk results, and two levels when high-risk results supplied at least half the weight. Inconsistency was downgraded one level for *I*^2^ > 50%; two levels were used when this coexisted with opposite-signed study effects and a prediction interval spanning the important-effect boundaries. A wide prediction interval alone at τ^2^ ≈ 0 was not used to duplicate an imprecision downgrade.

Imprecision was assessed against review-defined important-effect boundaries and conservative information-size benchmarks of 300 participants for performance and 200 for other critical/important outcomes. These are decision aids, not formally calculated optimal information sizes or validated universal thresholds. Two levels were deducted when N/benchmark<0.25 or the confidence interval crossed both important-effect boundaries; one level was deducted for N/benchmark<0.50 or an interval extending beyond either boundary. Boundaries were 2% for performance, 0.3 °C for core temperature, 5 beats/min for heart rate, 1 percentage point for body-mass loss, 3 percentage points for plasma-volume change, 1 scale unit for exertion/discomfort, 2 mmol/L for sodium, 5 mOsm/kg for osmolality and 0.2 L for urine volume. Indirectness and publication bias were considered separately.

### Sensitivity analyses and small-study effects

Sensitivity analyses examined paired and between-condition correlations, exclusion of high-risk results, exactly 60-min studies, preload-plus-performance-test protocols, and the carbohydrate co-ingestion contrast. Additional analyses considered a true no-fluid comparator, hot (dry-bulb temperature ≥30 °C) versus temperate environments, temperature site, fixed-timepoint versus average heart rate, omission of a change-score result, and alternative extracted measures. These *post hoc* analyses are reported in Supplementary Table S8. Leave-one-out analyses were not presented as new pooled estimates when only one study remained. Formal moderator regression was not undertaken because complete covariate data and adequate independent studies per covariate were lacking. Funnel plots and Egger tests were restricted to syntheses with at least 10 studies and interpreted cautiously (29).

The analysis was implemented in Python using NumPy, SciPy, pandas and Matplotlib. Machine-readable inputs, explicit selection specifications and the executable reproduction scripts are supplied in Supplementary material 2. AI-assisted coding support was used in preparing and checking the scripts; the supplied source values and documented analytical assumptions, rather than generated study data, determine the numerical results.

## Results

### Study selection and characteristics

The reconciled record contained 2,370 identified records, 1,568 duplicates, 802 screened records and 93 eligibility records. Sixty-nine were excluded and 24 studies were retained, with a summed study-reported sample of 225 participants (Figure 1). The decisions for the retained and reclassified source reports are documented in Supplementary Table S3 (30–57). Twenty-one studies contributed to at least one pairwise meta-analysis; Claveau et al. (32) contributed single-study comparison domains, and Montain and Coyle (49) was retained narratively without quantitative weight.

**Figure 1:**
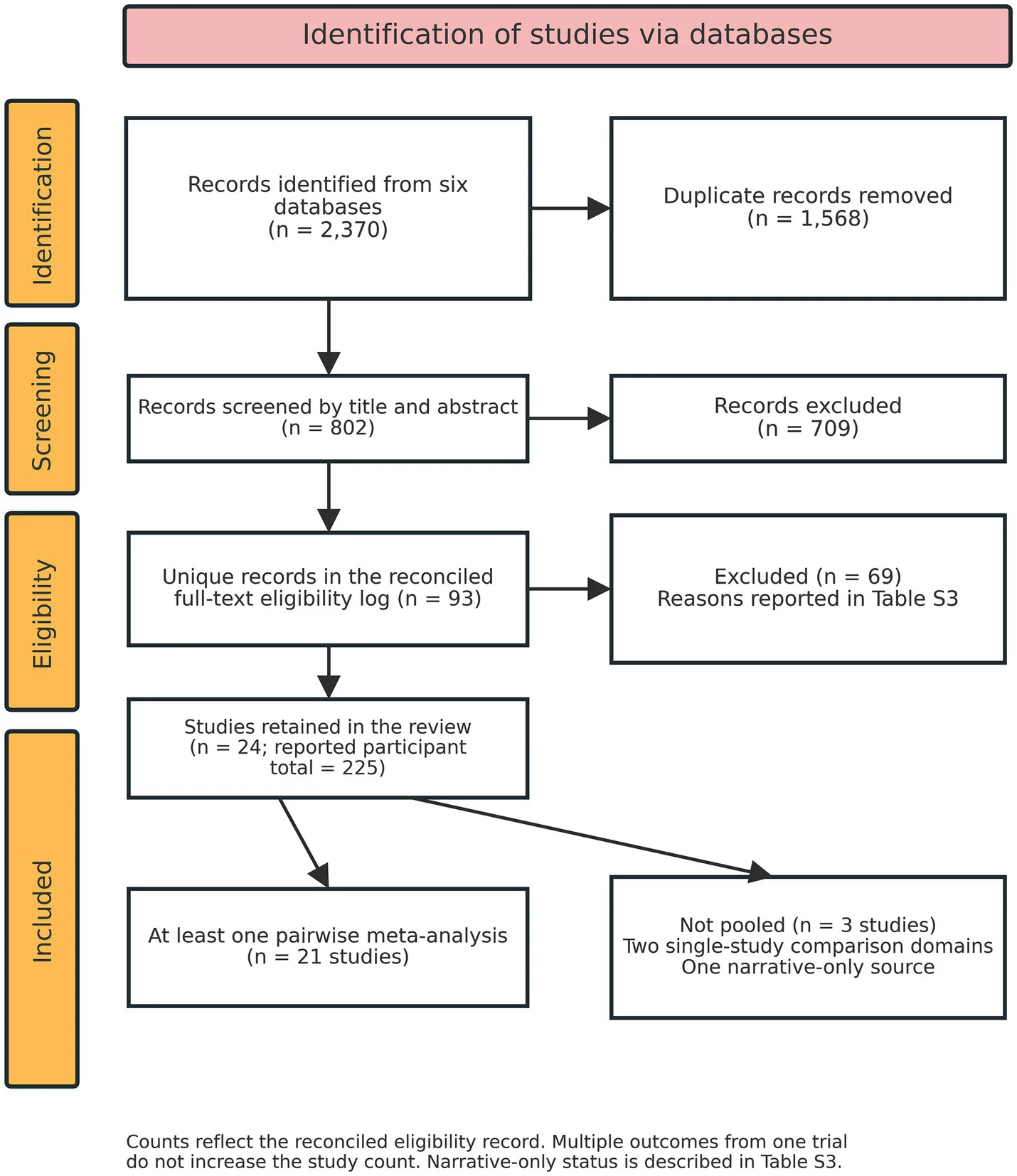
Study selection from the reconciled eligibility record. The 93 unique eligibility records comprise 24 retained studies and 69 exclusions. Twenty-one studies contribute to at least one pairwise synthesis; two represent single-study comparison domains and one is narrative only. Counts are not sums of arms, measurements or outcome-specific sample sizes. Supplementary Table S3 reports the exclusions and eligibility re-audit.

Backx and González-Alonso were incorporated after the eligibility re-audit. Four previously retained reports were removed: Funnell et al. (38) Sanders et al. (55) used programmed work–rest bouts; Che Muhamed randomized environmental order but conducted the no-fluid trials before fluid-ingestion trials; and Cosgrove did not satisfy the matched-fluid-volume requirement for a sodium comparison. Accordingly, Cosgrove’s pre-exercise sodium measurement no longer contributed to any synthesis. Unverified numerical entries for Montain and Coyle (48) were removed rather than treated as observed values.

The retained studies used crossover designs and had sample sizes of 6–20. The reported total comprised 170 men and 20 women, with sex unreported for 35 participants. Women represented 10.5% of participants with reported sex; no study recruited women exclusively. The summed N is not a count of observations and is an upper bound on distinct individuals where participant overlap between reports cannot be excluded. Outcome-specific samples were used; for example, Sage’s blood analyses included nine participants although 11 completed the exercise study. Study characteristics are given in Table 1, intervention details in Supplementary Table S4, and the recalculated study contributions in Supplementary Table S9.

**Table 1:** Characteristics of retained studies.

Study	Design	Participants	Exercise protocol and environment	Hydration strategy classification	Fluid volume during exercise	Sweat-loss replacement	Beverage sodium and carbohydrate	Core temperature site	Outcomes reported
Backx et al. (30)	Randomized crossover trial (4 trials; 3 randomized arms)	*N* = 8; trained male cyclists; age 29 ± 7 y; Male (8/8)	Acute: 60 min self-paced cycling time trial; 20 °C, 70% RH	Higher vs. lower fluid-replacement dose	900, 450 or 120 mL during exercise, plus a 6 mL/kg pre-exercise bolus	Achieved 74 ± 11%, 50 ± 10% and 33 ± 5% of body-mass loss replaced	Total 0.45 g sodium and 57.6 g carbohydrate, matched across all trials	Not measured	Distance; mean power; heart rate; body mass loss; fluid intake; stomach fullness
Claveau et al. (32)	Randomized crossover trial	*N* = 10; healthy endurance-trained men; age 27 ± 4 y; Male (10/10)	Acute: 120 km cycling (>3 h); 33 °C, 40% RH	Ad libitum drinking vs. thirst-driven drinking	2,815 ± 718 mL ad libitum vs. 2,714 ± 933 mL thirst-driven	Achieved 58 ± 11% vs. 54 ± 16%	Water	Rectal	Cycling time; fluid intake; percent sweat loss replaced; body mass loss; rectal temperature; heart rate; RPE; urine indices
Costa et al. (34)	Randomized crossover trial	*N* = 11; male endurance runners; age 34 ± 11 y; Male (11/11)	Acute: 2 h running at 70% VO2max plus early recovery sampling; 25 °C, 46% RH	Oral hydration vs. no or minimal fluid	Programmed water provision vs. total water restriction	Not reported	Water	Not measured	Body mass loss; plasma osmolality; plasma volume; heart rate; RPE; GI symptoms; I-FABP; breath hydrogen; inflammatory and endotoxin measures
Dion et al. (35)	Randomized crossover trial	*N* = 10; trained male distance runners; age 28 ± 7 y; Male (10/10)	Acute: half-marathon time trial (~90 min); 30 °C, 42% RH	Programmed or individualized vs. self-regulated drinking	2,060 ± 470 mL programmed vs. 580 ± 290 mL thirst-driven	Not reported	Water	Rectal	Half-marathon time; speed; pace; fluid intake; sweat loss/rate; body mass loss; rectal and mean body temperature; heart rate
Dugas et al. (36)	Randomized crossover trial (6 conditions; 5 eligible arms)	*N* = 6; trained male cyclists; age NR; Male (6/6)	Acute: 80 km cycling time trial; 33 °C	Oral hydration vs. no or minimal fluid; higher vs. lower fluid-replacement dose	0, 426 ± 69, 865 ± 164 or 1,324 ± 180 mL/h	Target 0, 33, 66 and 100%; achieved 0, 32.4 ± 2.9, 59.9 ± 6.4 and 94.6 ± 10.8%	Water	Rectal	Time-trial time; mean power; rectal temperature; sweat loss/rate; fluid intake; percent sweat loss replaced; body mass loss
Fallowfield et al. (37)	Randomized crossover trial	*N* = 8; healthy physically active adults; age 21.4 ± 0.7 y (SEM); 4 male, 4 female	Acute: treadmill running at 70% VO2max to exhaustion; 20 °C	Oral hydration vs. no or minimal fluid	929 ± 96 mL (pre-exercise bolus plus serial feedings) vs. no fluid	Not reported	Water	Rectal	Time to exhaustion; fluid-intake-corrected body mass loss; plasma volume; sweat rate; heart rate; rectal temperature; plasma sodium
González-Alonso et al. (39)	Randomized crossover trial	*N* = 7; endurance-trained men; age 27.6 ± 1.9 y (SEM); Male (7/7)	Acute: 2 h continuous cycling at 62% VO2max; 35.4 ± 0.2 °C, 48 ± 2% RH	Oral hydration vs. no or minimal fluid	3.60 L vs. 0.20 L; beverage temperature 38 °C; ingested at 20–95 min vs. 30–90 min	Achieved 95.1 ± 3.1% vs. 2.7 ± 1.6%	Identical total carbohydrate and electrolytes in both trials (70 g sports-drink powder: 69 g sugars, 518 mg sodium, 518 mg chloride, 118 mg potassium)	Esophageal and rectal	Esophageal and rectal temperature; heart rate; stroke volume; cardiac output; blood pressure; blood and plasma volume; serum osmolality and electrolytes; forearm and cutaneous blood flow; sweating rate; body mass loss
Jeker et al. (40)	Randomized crossover trial	*N* = 8; male endurance athletes; age 26 ± 6 y; Male (8/8)	Acute: 5 h cycling followed by a 20 km time trial; 30 °C, 35% RH	Programmed or individualized vs. self-regulated drinking	6,916 ± 1,124 mL programmed vs. 5,367 ± 925 mL thirst-driven	Not reported	Water	Rectal	20 km power and time; total performance time; fluid intake; sweat loss/rate; body mass loss; urine; rectal temperature; abdominal discomfort
Juett et al. (41)	Randomized crossover trial	*N* = 14; active healthy males; age 21 [20–22] y; Male (14/14)	Acute: up to 120 min cycling; approximately 35 °C, approximately 50% RH	Oral hydration vs. no or minimal fluid	5,360 ± 898 mL trial total vs. 4,185 ± 829 mL; 75–100 mL during exercise in the hypohydration trial	Target 100% of body mass loss vs. minimal	Water	Not measured	uKIM-1; uNGAL; body mass; plasma volume; serum and urine osmolality; heart rate; RPE; fluid intake
Kay and Marino (42)	Randomized crossover trial (4 conditions)	*N* = 7; healthy trained adults/cyclists; age NR; sex NR	Acute: 60 min self-paced cycling; moderate (21 °C) and warm-humid (31 °C)	Oral hydration vs. no or minimal fluid	1.3 ± 0.4 L (moderate) and 1.5 ± 0.4 L (warm) vs. no fluid	Target 100% of sweat loss	Water	Rectal	Distance; power; heart rate; core temperature; fluid intake; body mass change; sweat rate
Lambert et al. (43)	Randomized crossover trial (4 conditions; 2 eligible arms)	*N* = 20; healthy runners; age 22 ± 3 y; 11 male, 9 female	Acute: 60 min treadmill running at 70% VO2max; 24.4 ± 0.04 °C, 32.7 ± 0.55% RH	Oral hydration vs. no or minimal fluid	3 mL/kg every 10 min vs. no fluid	Not reported	Water placebo	Rectal	Body mass loss; sweat rate; heart rate; core temperature; RPE; GI symptoms and permeability
Lopez et al. (44)	Randomized crossover trial	*N* = 13; trained runners; age NR; 8 male, 5 female	Acute: 20 km trail-running race; hot outdoor conditions (WBGT 28.3 ± 1.9 °C)	Programmed or individualized vs. self-regulated drinking	1,700 ± 629 mL individualized vs. 749 ± 416 mL ad libitum	Achieved 64 ± 16% vs. 30 ± 14%	Water	Gastrointestinal	Finish time; fluid intake and needs; percent replacement; sweat rate; body mass loss; urine osmolality; GI temperature; heart rate
Maughan et al. (45)	Randomized crossover trial (5 trials; 2 eligible arms)	*N* = 12; healthy physically active young men; age 24 ± 1 y (SEM); Male (12/12)	Acute: cycling at 70% VO2max to exhaustion; 21 ± 1 °C	Oral hydration vs. no or minimal fluid	100 mL every 10 min vs. no drink	Not reported	Water	Rectal	Time to exhaustion; plasma volume; heart rate; rectal temperature; serum osmolality
McConell et al. (46)	Randomized crossover trial	*N* = 7; well-trained males; age 24 ± 1 y (SEM); Male (7/7)	Acute: 2 h cycling plus ride to exhaustion at 90% VO2peak; 21 °C	Oral hydration vs. no or minimal fluid; higher vs. lower fluid-replacement dose	0, 1.16 ± 0.05 or 2.32 ± 0.10 L	Target 0, 50 and 100%; achieved 0, 55.3 ± 2.0 and 113.8 ± 4.6%	Water	Rectal	Time to exhaustion; body mass loss; sweat loss; fluid intake; plasma volume; plasma sodium; urine; heart rate; rectal temperature
Montain and Coyle (47)	Randomized crossover trial (3 conditions; 2 eligible arms)	*N* = 7; trained cyclists; age 23 ± 3 y; sex NR	Acute: 2 h cycling at 62–67% VO2max; 33 °C	Oral hydration vs. no or minimal fluid	2,405 ± 103 mL vs. no fluid	Achieved 80.0 ± 2.2%	6% carbohydrate-electrolyte solution vs. no fluid; combined strategy, not carbohydrate matched	Rectal (*n* = 7); esophageal (*n* = 5)	Esophageal and rectal temperature; heart rate; plasma volume; skin blood flow; sweat rate; body mass loss; fluid intake; RPE
Montain and Coyle (48)	Randomized crossover trial (4 conditions)	*N* = 8; endurance-trained cyclists; age 23 ± 3 y (SD); sex NR	2 h cycling at 62–67% VO2max; 33 °C, 50% RH (published abstract)	Oral hydration vs. no or minimal fluid; higher vs. lower fluid-replacement dose	Graded fluid provision; quantitative intake data not verified	Approximately 0%, 20%, 48 and 81% replacement (published abstract)	Not verified from the full report	Esophageal (published abstract)	Narrative thermoregulatory and cardiovascular findings; no inverse-variance contribution.
Montain and Coyle (49)	Randomized crossover trial (4 timing conditions)	*N* = 7; trained cyclists; age NR; sex NR	Acute: 140 min cycling; 33 °C	Distributed vs. front-loaded fluid timing	1,173 ± 44 mL in all conditions; only ingestion timing differed	Achieved approximately 40.5%	Carbohydrate-electrolyte solution, identical composition and volume in all conditions	Esophageal and rectal	Esophageal and rectal temperature; heart rate; body mass loss; whole-body water loss; RPE
Nassis et al. (50)	Randomized crossover trial (2 modes × 2 hydration conditions)	*N* = 11; healthy non-heat-acclimatized men; age 25.7 ± 1.4 y (SEM); Male (11/11)	Acute: 90 min at 60% mode-specific VO2max; 23.8 ± 0.3°C, 58.8 ± 1.4% RH	Oral hydration vs. no or minimal fluid	1,165 ± 55 mL (cycling) and 1,365 ± 108 mL (running), including the post-warm-up portion, vs. no fluid	Target approximately 60%	Water	Rectal	Rectal temperature; heart rate; cardiac output; stroke volume; body mass loss; sweat rate; skin temperature; skin blood flow
Perreault-Brière et al. (51)	Randomized crossover trial	*N* = 9; trained endurance athletes; age 30 ± 9 y; 7 male, 2 female	Acute: 1 h cycling time trial; 30 °C, 50% RH	Oral hydration vs. no or minimal fluid; programmed or individualized vs. self-regulated drinking	1,606 ± 125 mL programmed, 565 ± 178 mL thirst-driven, 0 mL no fluid	Programmed targeted a body mass loss below approximately 0.5%	Water	Rectal	Distance; power; fluid intake; sweat loss; body mass loss; heart rate; plasma volume; plasma sodium; thirst; abdominal discomfort; temperature
Robinson et al. (52)	Randomized crossover trial	*N* = 8; endurance-trained male cyclists; age NR; Male (8/8)	Acute: 1 h cycling time trial; 20 °C, 60% RH	Oral hydration vs. no or minimal fluid	1.49 ± 0.14 L vs. no fluid	Target approximately 85% of fluid losses	Artificially sweetened water; no added sodium or carbohydrate	Rectal	Distance; power; heart rate; rectal and skin temperature; body mass/sweat loss; fluid retention; gastric residual; RPE; plasma sodium/osmolality
Sage et al. (53)	Randomized crossover trial	*N* = 11; male endurance-trained cyclists; age 35 ± 6 y; Male (11/11)	Acute: 180 min cycling plus time trial; 32 °C, 50% RH	Higher vs. lower fluid-replacement dose	1.125 ± 0.269 vs. 0.844 ± 0.202 L/h during the preload	Target 80% vs. 60% of sweat losses	Individualized sodium supplementation targeted comparable serum sodium concentrations; sodium intake was not identical.	Rectal	Time-trial completion time and power; body mass loss; fluid intake; rectal and skin temperature; plasma volume; serum sodium; urine
Sanders et al. (54)	Randomized crossover trial	*N* = 6; male cyclists; age NR; Male (6/6)	Acute: 90 min cycling at 65% peak oxygen uptake; 32 °C	Oral hydration vs. no or minimal fluid; higher vs. lower sodium/electrolyte dose	1.2 L water or 1.2 L saline vs. no fluid	Not reported	Water or 100 mmol/L sodium chloride solution at a matched volume; no carbohydrate	Not measured	Heart rate; plasma volume; plasma sodium; fluid and sodium balance; body-fluid compartment shifts
Walsh et al. (56)	Randomized counterbalanced crossover trial	*N* = 6; trained adult cyclists; age 25.5 y (mean; SD not reported); sex NR	Acute: 60 min cycling at 70% VO2peak plus cycling to exhaustion at 90% VO2peak; 32 °C, 60% RH	Oral hydration vs. no or minimal fluid	1.0 L in total (400 mL before exercise plus 120 mL every 10 min) vs. no fluid	Not reported	20 mmol/L sodium chloride solution; no carbohydrate	Rectal	Time to exhaustion; body mass loss; heart rate; RPE; rectal/skin temperature; plasma volume; sweat and electrolyte losses
Wijering et al. (57)	Randomized crossover trial	*N* = 11; endurance-trained males; age 36 ± 14 y; Male (11/11)	Acute: up to 3 h cycling; 34 °C, 65% RH	Higher vs. lower sodium/electrolyte dose	3.353 ± 0.666 vs. 3.296 ± 0.582 L; matched fluid replacement	Not reported	6% carbohydrate beverage with 60 vs. 21 mmol/L sodium at matched carbohydrate and volume	Rectal	Exercise time; fluid intake; sweat loss; body mass change; plasma sodium; plasma volume; heart rate; rectal temperature; thirst/RPE

Studies are ordered by first author. N is the study-reported sample; outcome-specific analyzed samples are provided in Supplementary Table S9. Montain and Coyle (48) is retained narratively and contributes no numerical weight. NR, not reported; RH, relative humidity; RPE, rating of perceived exertion. Mean ± SD unless the source states otherwise.

### Oral hydration versus no or minimal fluid

Table 2 summarizes the 22 pooled critical/important outcomes. For time-trial/fixed-duration performance, the estimate was −0.1% (−3.6 to 3.5%) (four studies, 30 participants; *I*^2^ = 1.8%; prediction interval −6.2 to 6.4%). Time to exhaustion favored hydration on average, 34.1% (11.9–60.7%) (three studies, 21 participants; *I*^2^ = 0%), but the prediction interval ranged from −21.4 to 128.8%. Both were very-low-certainty evidence, and the exhaustion estimate should not be read as an established benefit (Figures 2A,B).

**Table 2:** Summary of findings for pooled critical and important outcomes.

Comparison	Outcome	Priority	Studies (N)	Effect (95% CI)	95% prediction interval	I^2^ (%)	Certainty	Interpretation
Oral hydration vs. no or minimal fluid	Body mass loss (%)	Important	7 (72)	−2.63 (−3.58 to −1.69) percentage points	−5.41 to 0.14	98.3	Very low	Smaller measured deficit; not an independent benefit.
Oral hydration vs. no or minimal fluid	Core temperature	Critical	9 (80)	−0.38 (−0.74 to −0.02) °C	−1.46 to 0.69	87.3	Very low	Mean favors hydration; benefit remains uncertain.
Oral hydration vs. no or minimal fluid	Heart rate	Important	12 (115)	−6.82 (−11.16 to −2.48) beats/min	−20.38 to 6.73	83.3	Very low	Mean favors hydration; benefit remains uncertain.
Oral hydration vs. no or minimal fluid	Plasma sodium concentration	Important	4 (29)	−4.37 (−10.18 to 1.43) mmol/L	−21.32 to 12.57	91.7	Very low	Very uncertain; interval crosses the null.
Oral hydration vs. no or minimal fluid	Plasma volume change (%)	Important	7 (60)	3.28 (0.42 to 6.14) percentage points	−4.29 to 10.84	82.7	Very low	Mean favors hydration; benefit remains uncertain.
Oral hydration vs. no or minimal fluid	Rating of perceived exertion (Borg 6–20)	Important	3 (39)	−0.54 (−2.11 to 1.03) AU	−7.55 to 6.47	38.8	Very low	Very uncertain; interval crosses the null.
Oral hydration vs. no or minimal fluid	Serum/plasma osmolality	Important	3 (26)	−12.75 (−34.19 to 8.69) mOsm/kg	−133.64 to 108.14	92.5	Very low	Very uncertain; interval crosses the null.
Oral hydration vs. no or minimal fluid	Time-to-exhaustion performance	Critical	3 (21)	34.1% (11.9–60.7%)	−21.4 to 128.8%	0.0	Very low	Mean favors hydration; benefit remains uncertain.
Oral hydration vs. no or minimal fluid	Time-trial/fixed-duration performance	Critical	4 (30)	−0.1% (−3.6 to 3.5%)	−6.2 to 6.4%	1.8	Very low	Very uncertain; interval crosses the null.
Oral hydration vs. no or minimal fluid	Urine volume (L)	Important	4 (30)	0.06 (−0.05 to 0.16) L	−0.15 to 0.26	28.3	Very low	Very uncertain; interval crosses the null.
Programmed or individualized vs. self-regulated drinking	Abdominal discomfort	Important	2 (17)	0.23 (−1.46 to 1.91) AU	Not estimable (*k* < 3)	0.0	Very low	Very uncertain; only two studies.
Programmed or individualized vs. self-regulated drinking	Body mass loss (%)	Important	4 (40)	−1.47 (−1.96 to −0.99) percentage points	−2.61 to −0.34	62.4	Very low	Smaller measured deficit; not an independent benefit.
Programmed or individualized vs. self-regulated drinking	Core temperature	Critical	3 (31)	−0.23 (−0.58 to 0.12) °C	−1.26 to 0.79	0.0	Very low	Very uncertain; interval crosses the null.
Programmed or individualized vs. self-regulated drinking	Heart rate	Important	2 (19)	−3.24 (−20.14 to 13.65) beats/min	Not estimable (*k* < 3)	0.0	Very low	Very uncertain; only two studies.
Programmed or individualized vs. self-regulated drinking	Time-trial/fixed-duration performance	Critical	3 (27)	0.7% (−4.4 to 6.2%)	−13.7 to 17.6%	0.0	Very low	Very uncertain; interval crosses the null.
Programmed or individualized vs. self-regulated drinking	Urine volume (L)	Important	2 (17)	0.39 (−4.81 to 5.59) L	Not estimable (*k* < 3)	85.0	Very low	Very uncertain; only two studies.
Higher vs. lower fluid-replacement dose	Body mass loss (%)	Important	3 (26)	−1.10 (−2.54 to 0.34) percentage points	−9.28 to 7.09	94.0	Very low	Mean deficit smaller; interval includes no difference.
Higher vs. lower fluid-replacement dose	Core temperature	Critical	2 (17)	−0.30 (−1.20 to 0.60) °C	Not estimable (*k* < 3)	0.0	Very low	Very uncertain; only two studies.
Higher vs. lower fluid-replacement dose	Plasma volume change (%)	Important	2 (16)	5.18 (−7.21 to 17.57) percentage points	Not estimable (*k* < 3)	0.0	Very low	Very uncertain; only two studies.
Higher vs. lower fluid-replacement dose	Time-trial/fixed-duration performance	Critical	3 (25)	2.7% (−8.8 to 15.6%)	−43.3 to 85.9%	67.4	Very low	Very uncertain; interval crosses the null.
Higher vs. lower fluid-replacement dose	Urine volume (L)	Important	3 (24)	0.13 (−0.05 to 0.30) L	−0.39 to 0.64	0.0	Very low	Very uncertain; interval crosses the null.
Higher vs. lower sodium/electrolyte dose	Plasma volume change (%)	Important	2 (17)	1.78 (−5.24 to 8.81) percentage points	Not estimable (*k* < 3)	0.0	Very low	Very uncertain; only two studies.

Mean differences are first-named strategy minus comparator. Positive performance differences indicate better performance. Prediction intervals are reported only for *k* ≥ 3. GRADE rules are review-specific and *post hoc*; single-study critical/important estimates and domain reasons are provided in Supplementary Table S7. Supporting manipulation outcomes are reported separately in Supplementary Table S10.

**Figure 2:**
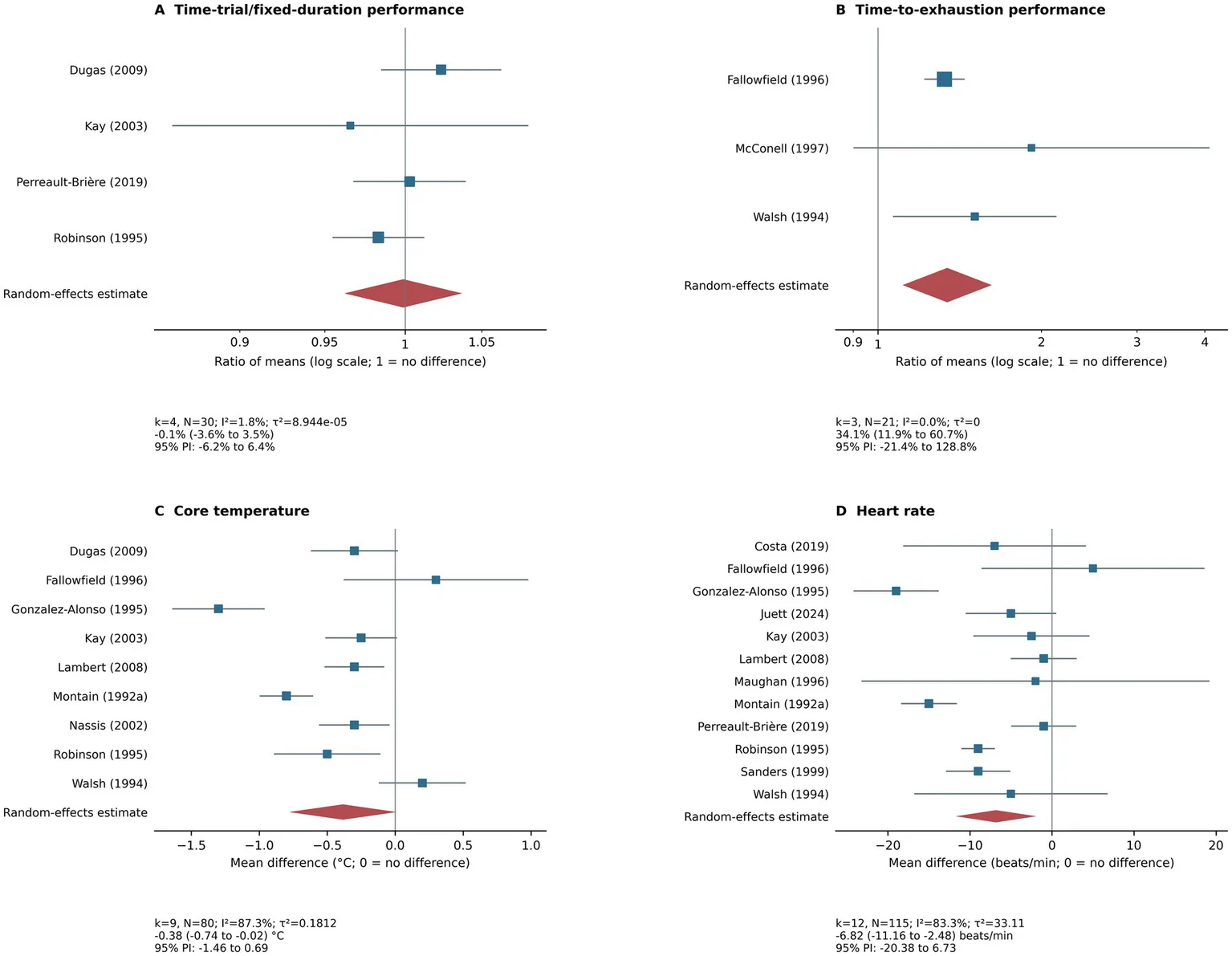
Oral hydration versus no or minimal fluid: Performance, core temperature and heart rate. **(A,B)** Direction-harmonized ratios of means on logarithmic axes; values above 1 favor hydration. **(C,D)** Mean differences, hydration minus comparator; values below 0 indicate lower temperature or heart rate with hydration. Squares represent studies and increase in size with random-effects weight; diamonds show REML estimates with modified Hartung–Knapp 95% confidence intervals. Each panel reports *k*, *N*, *I*^2^, *τ*^2^ and the 95% prediction interval (*PI*). All four estimates are very low certainty.

Core temperature was −0.38 (−0.74 to −0.02) °C (nine studies, 80 participants; *I*^2^ = 87.3%; prediction interval −1.46 to 0.69 °C). Heart rate was −6.82 (−11.16 to −2.48) beats/min (12 studies, 115 participants; *I*^2^ = 83.3%; prediction interval −20.38 to 6.73 beats/min). These averages favored hydration but had substantial heterogeneity, prediction intervals crossing the null and very-low certainty (Figures 2C,D).

Body-mass loss was −2.63 (−3.58 to −1.69) percentage points (seven studies, 72 participants; *I*^2^ = 98.3%; prediction interval −5.41 to 0.14). This largely reflects the volume ingested and is not an independent physiological benefit. Plasma-volume change was 3.28 (0.42–6.14) percentage points (seven studies, 60 participants; *I*^2^ = 82.7%; prediction interval −4.29 to 10.84). Perceived exertion remained uncertain, −0.54 (−2.11 to 1.03) AU (three studies, 39 participants; very low certainty) (Figures 3A–C).

**Figure 3:**
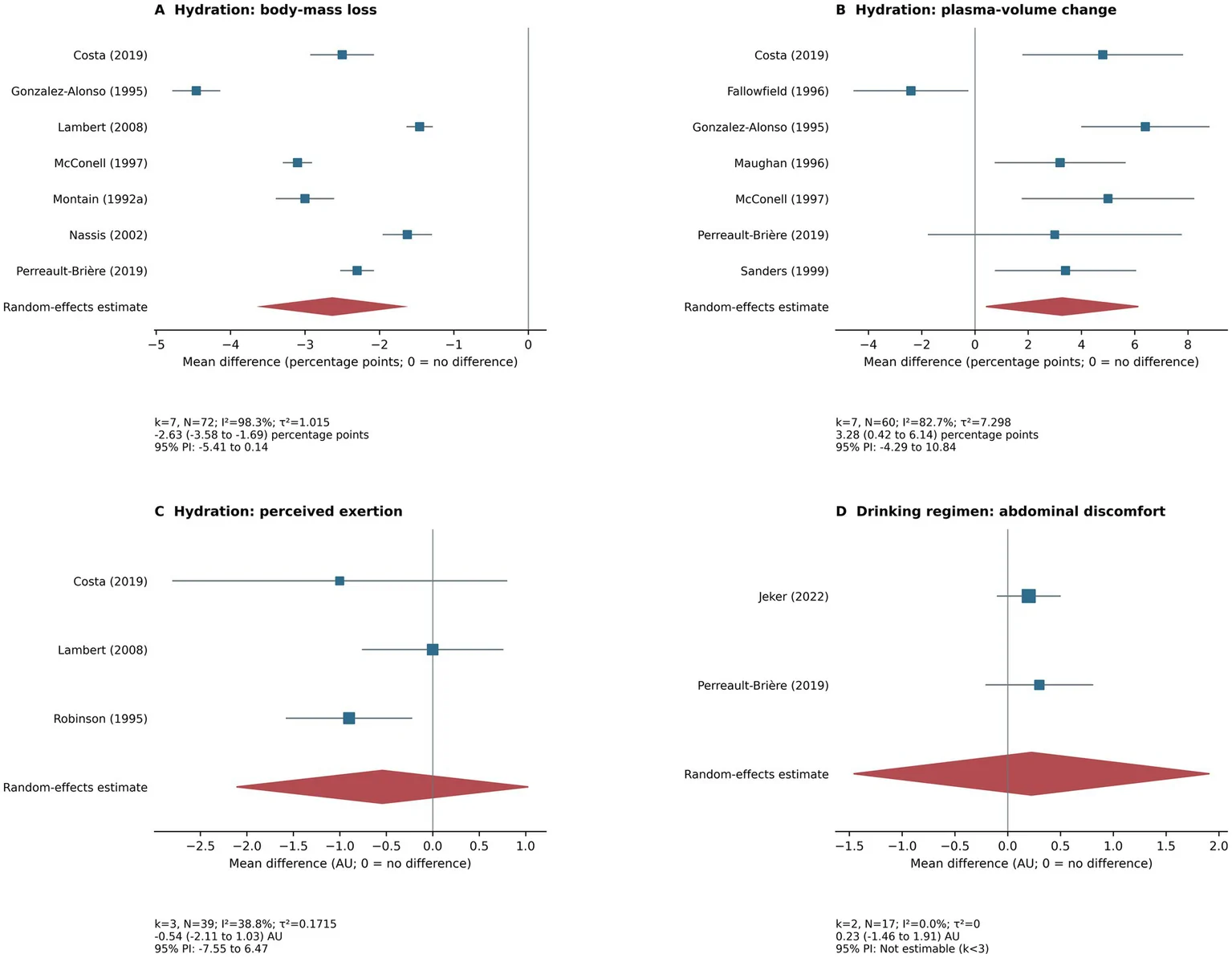
Fluid balance, exertion and abdominal discomfort. **(A)** Hydration versus no/minimal fluid, body-mass loss; **(B)** The same comparison, plasma-volume change. **(C)** The same comparison, perceived exertion. **(D)** Programmed/individualized versus self-regulated drinking, abdominal discomfort. Values below 0 indicate a smaller deficit or lower score in **A, C** and **D**; Values above 0 in **B** indicate a less negative plasma-volume change. A smaller body-mass deficit partly follows directly from the fluid ingested and is not an independent physiological benefit. A prediction interval is not estimated for *k* < 3.

Plasma sodium concentration was −4.37 (−10.18 to 1.43) mmol/L (four studies, 29 participants), and serum/plasma osmolality was −12.75 (−34.19 to 8.69) mOsm/kg (three studies, 26 participants). Both intervals crossed the null and heterogeneity was high. Urine volume was also uncertain (Supplementary Figure S1). Other fluid-balance and intervention-manipulation outcomes were treated as supporting analyses rather than additional primary efficacy claims (Supplementary Table S10).

### Programmed or individualized versus self-regulated drinking

Time-trial/fixed-duration performance was 0.7% (−4.4 to 6.2%) (three studies, 27 participants; *I*^2^ = 0%). Body-mass loss was −1.47 (−1.96 to −0.99) percentage points (four studies, 40 participants; *I*^2^ = 62.4%; prediction interval −2.61 to −0.34). This difference indicates a smaller measured deficit under larger-volume regimens, not a separate physiological benefit. Core temperature and heart-rate intervals crossed the null (Supplementary Figure S2). Abdominal discomfort was 0.23 (−1.46 to 1.91) AU and urine volume was 0.39 (−4.81 to 5.59) L (each two studies, 17 participants). Certainty was very low, particularly for unblinded symptom outcomes (Figure 3D).

### Higher versus lower fluid-replacement dose

Time-trial/fixed-duration performance was 2.7% (−8.8 to 15.6%) (three studies, 25 participants; *I*^2^ = 67.4%). McConell’s time-to-exhaustion contrast remained a separate single-study estimate. Body-mass loss was −1.10 (−2.54 to 0.34) percentage points (three studies, 26 participants). Core temperature was −0.30 (−1.20 to 0.60) °C (two studies, 17 participants) and plasma-volume change was 5.18 (−7.21 to 17.57) percentage points (two studies, 16 participants). The latter analyses use Sage’s latest available end-preload results; no later same-outcome measurement was available. Intervals were wide and certainty was very low (Supplementary Figure S3).

### Higher versus lower sodium/electrolyte dose

Under the fluid/carbohydrate-matching rule, only Sanders et al. (54) and Wijering contributed eligible sodium contrasts. The pooled plasma-volume change was 1.78 (−5.24 to 8.81) percentage points (two studies, 17 participants; *I*^2^ = 0%; very low certainty). A prediction interval was not estimated for two studies. Other sodium outcomes were single-study direct evidence (Supplementary Figure S4; Supplementary Table S5). Wijering’s capped exercise duration was described as a protocol-completion measure, not pooled as time-trial performance. No eligible pooled performance estimate supports an optimal sodium dose.

### Single-study and narrative evidence

Claveau’s ad libitum-versus-thirst-driven comparison and Montain’s distributed-versus-front-loaded timing comparison were retained as direct single-study evidence. Montain and Coyle (48) described a randomized four-dose, 2-h cycling experiment with graded dehydration in the published summary, but its numerical results and full-text risk-of-bias details could not be verified; it received no quantitative weight. Supplementary Table S5 also retains point estimates lacking compatible precision, incompatible performance measures and nonselected timepoints.

### Exploratory within-study dose–response results

Dugas (six participants, time trial) and McConell (seven participants, time to exhaustion) yielded within-study slopes of 0.3% (95% CI − 0.1 to 0.7%) and 6.6% (−1.0 to 14.9%) per 10 percentage points of target replacement, respectively. They were not pooled across constructs (Supplementary Figure S5). Target and achieved doses differed: Dugas achieved approximately 0, 32.4, 59.9, and 94.6% for targets of 0, 33, 66, and 100%, whereas McConell achieved 0, 55.3 and 113.8% for targets of 0, 50, and 100%. These very small, assumption-dependent analyses do not establish linearity or an optimal dose.

### Sensitivity analyses and small-study effects

With assumed paired correlations of 0.25 and 0.75, hydration core-temperature estimates ranged from −0.39 to −0.38 °C and heart-rate estimates from −7.00 to −6.58 beats/min. Restricting the comparator to true no fluid gave −0.29 °C (−0.57 to −0.01; eight studies) and −5.74 beats/min (−10.13 to −1.34; 10 studies). These are descriptive restrictions, not proof that comparator type caused between-study differences. Removing the Montain and Coyle (47) carbohydrate co-ingestion contrast changed core temperature to −0.33 °C (−0.72 to 0.07), showing sensitivity of the thermal inference to that contrast.

Rectal-only core-temperature estimates were −0.29 °C (−0.57 to −0.01; eight studies), and rectal/esophageal estimates were −0.38 °C (−0.74 to −0.02; nine studies). Hot and temperate subgroups were sparse, with no reliable between-subgroup causal inference. Excluding exactly 60-min or preload-plus-performance-test protocols and substituting alternative extracted measures did not remedy the small information size. Leave-one-out results are shown in Supplementary Figure S6 and Supplementary Table S8; when only one study remained, no pooled result was reported. Only heart rate reached 10 studies (*k* = 12; Egger *p* = 0.413). The funnel plot could not exclude reporting bias because of limited power and substantial heterogeneity (Supplementary Figure S7).

### Risk of bias and certainty of evidence

The source assessment file contained 194 outcome-specific judgments. Objective results predominantly had some concerns, reflecting incomplete allocation/analysis-plan reporting; subjective exertion and discomfort results were at high risk when participants knew the strategy. Weighted contributions for the main models are summarized in Table 3 and Figure 4. The 40 estimable critical/important comparison–outcome pairs, including 18 single-study pairs, were all rated very low certainty under the consistently applied rules. The TTE result was downgraded for risk of bias and twice for imprecision because 21 participants represented only 0.07 of the review-defined information benchmark; its statistically non-null confidence interval did not override that limitation. Full reasons are given in Supplementary Table S7.

**Table 3:** Result-specific risk-of-bias weights in the main syntheses.

Comparison	Outcome	Studies	Some-concerns weight	High-risk weight
Oral hydration vs. no or minimal fluid	Body mass loss (%)	7	100.0%	0.0%
Oral hydration vs. no or minimal fluid	Core temperature	9	100.0%	0.0%
Oral hydration vs. no or minimal fluid	Heart rate	12	100.0%	0.0%
Oral hydration vs. no or minimal fluid	Plasma sodium concentration	4	100.0%	0.0%
Oral hydration vs. no or minimal fluid	Plasma volume change (%)	7	100.0%	0.0%
Oral hydration vs. no or minimal fluid	Rating of perceived exertion (Borg 6–20)	3	0.0%	100.0%
Oral hydration vs. no or minimal fluid	Serum/plasma osmolality	3	100.0%	0.0%
Oral hydration vs. no or minimal fluid	Time-to-exhaustion performance	3	100.0%	0.0%
Oral hydration vs. no or minimal fluid	Time-trial/fixed-duration performance	4	100.0%	0.0%
Oral hydration vs. no or minimal fluid	Urine volume (L)	4	100.0%	0.0%
Programmed or individualized vs. self-regulated drinking	Abdominal discomfort	2	0.0%	100.0%
Programmed or individualized vs. self-regulated drinking	Body mass loss (%)	4	100.0%	0.0%
Programmed or individualized vs. self-regulated drinking	Core temperature	3	100.0%	0.0%
Programmed or individualized vs. self-regulated drinking	Heart rate	2	100.0%	0.0%
Programmed or individualized vs. self-regulated drinking	Time-trial/fixed-duration performance	3	100.0%	0.0%
Programmed or individualized vs. self-regulated drinking	Urine volume (L)	2	100.0%	0.0%
Higher vs. lower fluid-replacement dose	Body mass loss (%)	3	100.0%	0.0%
Higher vs. lower fluid-replacement dose	Core temperature	2	100.0%	0.0%
Higher vs. lower fluid-replacement dose	Plasma volume change (%)	2	100.0%	0.0%
Higher vs. lower fluid-replacement dose	Time-trial/fixed-duration performance	3	100.0%	0.0%
Higher vs. lower fluid-replacement dose	Urine volume (L)	3	100.0%	0.0%
Higher vs. lower sodium/electrolyte dose	Plasma volume change (%)	2	100.0%	0.0%

No low-risk result contributed weight to these main models. The underlying domain-level judgments are supplied in data/04_source_risk_of_bias.csv in Supplementary material 2.

**Figure 4:**
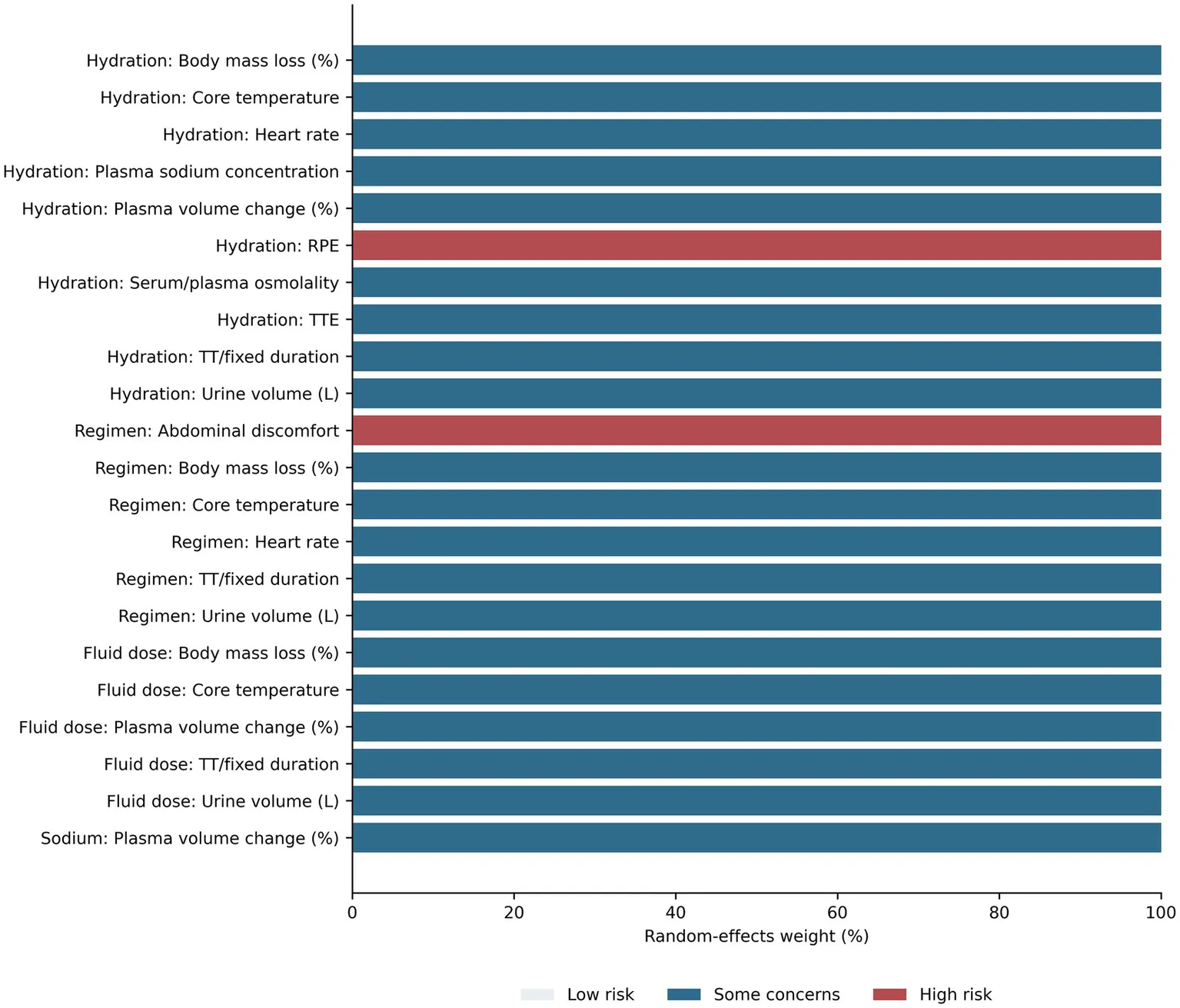
Result-specific risk-of-bias contributions to the main meta-analyses. Bars show the percentage of random-effects weight from low-risk, some-concerns, and high-risk results. Judgments refer to the specific result entering each model, not a worst-case label applied to every outcome in a study. TT, time trial; TTE, time to exhaustion; RPE, rating of perceived exertion.

## Discussion

The effect of drinking during prolonged endurance exercise depends on what is measured and what is compared. The average hydration estimate favored time to exhaustion, core temperature and heart rate, whereas time-trial/fixed-duration performance was close to the null. Substantial uncertainty, including prediction intervals crossing the null and very-low-certainty evidence, prevents treating these averages as established effects. Programmed or individualized drinking changed measured fluid balance more consistently than performance; evidence for fluid-dose and sodium-dose decisions remained sparse.

Time-to-exhaustion and time-trial/fixed-duration tests have different response properties. Open-ended exhaustion tests generally have greater within-person variability and may amplify expectancy, discomfort and motivation, whereas self-paced trials more closely resemble competitive pacing (14). Previous syntheses also found that hydration effects depend on protocol and environmental context (10–12, 14). The revised analysis therefore preserves the construct distinction in both pairwise and dose–response reporting rather than retaining a mixed-performance slope.

The thermal and cardiovascular averages are compatible with effects of fluid availability on plasma volume, cardiovascular drift, skin blood flow and heat dissipation (1, 3–5). They nevertheless summarize a dispersed collection of contrasts, including laboratory heat stress and strong fluid restriction. The estimate changed when co-ingestion or measurement restrictions were applied, and the subgroup cells were too small to establish a causal explanation. Trials comparing a carbohydrate-containing drink with no fluid estimate a combined drinking strategy; without a network/component model, carbohydrate cannot be considered statistically adjusted away.

A smaller measured body-mass deficit is expected when more fluid is consumed. It does not by itself establish better performance, safer thermoregulation or a clinically important physiological benefit. This distinction is particularly relevant to programmed drinking and fluid-dose comparisons (8, 13, 35, 40, 44, 51). Sodium evidence was reduced further by enforcing fluid-volume matching: studies in which sodium increased ad libitum drinking estimate a different strategy effect and were not combined with fixed-volume sodium comparisons. The remaining evidence does not support a universal sodium-performance claim (53, 54, 57).

Gastrointestinal evidence was limited. The two pooled regimen studies assessed unblinded abdominal discomfort and yielded a wide interval. Backx also reported fullness under different fluid volumes, but this measure was not merged with a different discomfort scale. Beverage composition, volume, intensity, recent feeding and individual tolerance all require consideration (16, 17, 30, 34, 43). Neither the symptom evidence nor the fluid-dose evidence establishes a single volume that maximizes benefit without discomfort.

The principal strengths are preservation of direct contrasts, separation of performance constructs, explicit outcome-level selection and variance calculations, and a portable reproduction archive. All retained studies have an evidence destination, including sources that could not contribute inverse-variance precision. The distinction between critical/important outcomes and supporting manipulation measurements limits the risk of presenting numerous correlated physiological findings as independent confirmations.

Important limitations remain. Samples were small and predominantly male: only 20 of 190 participants with reported sex were women. Perreault-Brière tested women in the follicular phase, but menstrual-cycle phase and hormonal contraceptive use were not consistently reported across studies; no female-only study was included (51). Generalization to women, untrained adults and field competition is therefore uncertain. Many results relied on assumed paired correlations or approximate precision recovered from rounded intervals, and study-level sample totals cannot establish uniqueness across reports.

Original database execution histories and database-specific retrieval counts were not retained, a reproducible forward-citation log was unavailable, original authors were not contacted, and complete source reports for some reviewer-nominated examples were not available for a final eligibility/extraction judgment. The absence of these reports is a coverage limitation, not evidence that they are ineligible. Below et al. (58) and Fritzsche et al. (59) include factorial contrasts that cannot be excluded solely on carbohydrate grounds; their full eligibility and extraction assessments remain incomplete. The numerical entries for Montain and Coyle (48) were also withheld from synthesis rather than accepted without verification. Small syntheses may change materially if additional eligible data become available.

The change from the original component-network plan to direct pairwise synthesis, the unverified prospective status of individual analytical decisions, the *post hoc* selection rules and review-defined GRADE benchmarks also limit confidence. This review did not estimate independent component effects, treatment rankings or a validated dose–response relation. All subgroup analyses are exploratory and may reflect correlated design features. These limitations warrant cautious interpretation rather than numerical dosing recommendations.

For practice, individualized planning should consider expected sweat and sodium losses, duration, heat exposure, access to drinks, gastrointestinal tolerance and the consequences of both under- and overdrinking (2, 7–9). For research, larger randomized trials should report allocation methods and paired precision, include women, distinguish self-paced and exhaustion endpoints, and compare fluid or sodium doses with co-interventions controlled. Achieved replacement and adverse symptoms should be reported alongside prescribed intake.

## Conclusion

Pooled estimates favored oral hydration over no or minimal fluid for time to exhaustion and some physiological outcomes, but certainty was very low and relevant prediction intervals included no benefit. No clear average benefit was demonstrated for self-paced time-trial/fixed-duration performance. Programmed drinking changed measured fluid balance more consistently than performance, and the evidence does not define an optimal fluid-replacement or sodium dose. These findings should inform cautious, individualized planning, not universal performance prescriptions.

## Data Availability

The original contributions presented in the study are included in the article/Supplementary material, further inquiries can be directed to the corresponding author.
