# Supplementary material for: Effects of during-exercise oral hydration strategies on endurance performance and thermophysiological strain: a systematic review with meta-analysis
Source: Front Nutr. 2026 Oct 27;13:1968824. doi: 10.3389/fnut.2026.1968824 (PMC13747748; doi:10.3389/fnut.2026.1968824)
Supplement: Supplementary file 2 [file Supplementary_file_1.docx]

**Supplementary Material 1**

Effects of during-exercise oral hydration strategies on endurance performance and thermophysiological strain: a systematic review with meta-analysis

Tables S1–S10; Figures S1–S7.

**Table S1. Available registration plan compared with the analyses performed.**

| **Item** | **Available plan** | **Analysis performed** | **Status / explanation** |
| --- | --- | --- | --- |
| Review question and population | Healthy adults; during-exercise oral hydration; continuous endurance exercise ≥60 min. | Retained, with explicit handling of measurement interruptions and hydration-contrast randomization. | Operational clarifications documented. |
| Study design | Randomized crossover or parallel trials; explicit random allocation. | Randomization must apply to hydration, not only environment. | Che Muhamed removed after source-level re-audit. |
| Sodium comparison | Fluid volume and carbohydrate matched; sodium may be delivered in drinks or capsules. | Applied to the actual compared conditions. | Cosgrove excluded because fluid was not matched. |
| Combined fluid/CHO contrast | Fluid versus no fluid may use a CHO-containing beverage without a water-only arm; CHO proposed as a component/covariate. | Retained as a combined drinking strategy and examined by exclusion sensitivity; no component adjustment performed. | No claim of an isolated water effect. |
| Duration | Total exercise ≥60 min, including a preload/dehydration phase. | Preload and performance within a visit counted; separate visits not counted; repeated programmed rests excluded. | Funnell and Sanders (2001) removed. |
| Network/component model | NMA followed by additive CNMA; independent component effects. | Not performed; direct pairwise random-effects synthesis. | Substantive deviation: sparse, heterogeneous contrasts did not support secure component interpretation. |
| Rankings and inconsistency | P-scores/SUCRA; design-by-treatment and local inconsistency tests. | Not performed. | No network model or rankings are claimed. |
| Certainty | CINeMA for networks; GRADE for key pairwise estimates. | GRADE for critical/important pairwise and single-study estimates; no CINeMA. | Specific thresholds and benchmark rules were developed post hoc. |
| Performance metric | MD/SMD with harmonized direction. | Log ratio of means, back-transformed to percentages. | Post hoc metric choice; TT/fixed-duration and TTE separated. |
| Paired data | Extract paired effects; impute correlation where needed and test sensitivity. | Reported paired precision preferred; r=.50 with .25/.75 sensitivity; study-specific calculations supplied. | General paired principle planned; exact values/selection rules post hoc. |
| Outcome/timepoint selection | Outcome domains specified; no detailed ranking of measures/sites. | Explicit within-construct, site and timepoint hierarchy; baseline-only values excluded. | Post hoc operational hierarchy. |
| Moderators | Inspect potential effect modifiers; network meta-regression where feasible. | No meta-regression; exploratory subgroup/restriction analyses. | Sparse complete covariates and dependent strata. |
| Dose response | Dose components in the network model. | Separate exploratory within-study GLS slopes; no pooled mixed-performance slope. | Post hoc exploratory analysis, not a dosing model. |
| Search documentation | Detailed database list and executed strings not contained in the supplied concise plan. | Six databases to 1 June 2026; documented reconstructed strings. | Original execution histories and database-specific counts not retained; reconstructed strings are not authenticated original search records. |
| Protocol availability | No separately deposited full protocol. | Registration plan used for this comparison; no timestamped version history. | The table does not independently authenticate an official historical registry version. |

The available concise registration plan proposed component network meta-analysis. This table distinguishes documented plan content from post hoc decisions; it does not verify prospective registration of individual analyses because no timestamped version history of the complete analytical plan is available. Registration ID and date are reported in the manuscript. CHO, carbohydrate; CNMA, component network meta-analysis; NMA, network meta-analysis; TT, time trial; TTE, time to exhaustion.

**Table S2. Reconstructed database-specific search strategies.**

| **Database** | **Reconstructed search strategy** |
| --- | --- |
| #1 PubMed | (("Endurance Training"[Mesh] OR "Running"[Mesh] OR "Bicycling"[Mesh] OR "Athletic Performance"[Mesh] OR endurance exercis*[tiab] OR endurance train*[tiab] OR prolonged exercis*[tiab] OR long-distance[tiab] OR marathon*[tiab] OR ultramarathon*[tiab] OR triathlon*[tiab] OR running[tiab] OR cycling[tiab] OR bicycl*[tiab] OR rowing[tiab]) AND ("Drinking"[Mesh] OR "Water-Electrolyte Balance"[Mesh] OR "Dehydration"[Mesh] OR "Sodium, Dietary"[Mesh] OR hydrat*[tiab] OR rehydrat*[tiab] OR dehydrat*[tiab] OR hypohydrat*[tiab] OR "fluid intake"[tiab] OR "fluid replacement"[tiab] OR "fluid strategy"[tiab] OR "fluid strategies"[tiab] OR "drinking strategy"[tiab] OR "drinking strategies"[tiab] OR "ad libitum"[tiab] OR "thirst-driven"[tiab] OR "programmed drinking"[tiab] OR "water ingestion"[tiab] OR electrolyte*[tiab] OR sodium[tiab]) AND (randomized controlled trial[pt] OR controlled clinical trial[pt] OR random*[tiab] OR crossover[tiab] OR cross-over[tiab] OR counterbalanc*[tiab])) |
| #2 Web of Science Core Collection | TS=((endurance NEAR/3 (exercise* OR train* OR sport*)) OR "prolonged exercise" OR "long-distance" OR marathon* OR ultramarathon* OR triathlon* OR running OR cycling OR bicycl* OR rowing) AND TS=(hydrat* OR rehydrat* OR dehydrat* OR hypohydrat* OR "fluid intake" OR "fluid replacement" OR "fluid strategy" OR "fluid strategies" OR "drinking strategy" OR "drinking strategies" OR "ad libitum" OR "thirst-driven" OR "programmed drinking" OR "water ingestion" OR electrolyte* OR sodium) AND TS=(random* OR crossover OR "cross-over" OR counterbalanc*) |
| #3 Embase.com | ('endurance training'/exp OR 'running'/exp OR 'cycling'/exp OR 'rowing'/exp OR 'triathlon'/exp OR 'athletic performance'/exp OR (endurance NEAR/3 (exercise* OR train* OR sport*)):ti,ab,kw OR 'prolonged exercise':ti,ab,kw OR 'long distance':ti,ab,kw OR marathon*:ti,ab,kw OR ultramarathon*:ti,ab,kw OR triathlon*:ti,ab,kw OR running:ti,ab,kw OR cycling:ti,ab,kw OR bicycl*:ti,ab,kw OR rowing:ti,ab,kw) AND ('drinking'/exp OR 'water electrolyte balance'/exp OR 'dehydration'/exp OR 'rehydration'/exp OR 'sodium intake'/exp OR hydrat*:ti,ab,kw OR rehydrat*:ti,ab,kw OR dehydrat*:ti,ab,kw OR hypohydrat*:ti,ab,kw OR 'fluid intake':ti,ab,kw OR 'fluid replacement':ti,ab,kw OR 'fluid strategy':ti,ab,kw OR 'drinking strategy':ti,ab,kw OR 'ad libitum':ti,ab,kw OR 'thirst driven':ti,ab,kw OR 'programmed drinking':ti,ab,kw OR 'water ingestion':ti,ab,kw OR electrolyte*:ti,ab,kw OR sodium:ti,ab,kw) AND ('randomized controlled trial'/exp OR 'crossover procedure'/exp OR random*:ti,ab,kw OR crossover:ti,ab,kw OR 'cross over':ti,ab,kw OR counterbalanc*:ti,ab,kw) |
| #4 Cochrane Library | ([mh "Endurance Training"] OR [mh Running] OR [mh Bicycling] OR [mh "Athletic Performance"] OR ((endurance NEAR/3 (exercise* OR train* OR sport*)) OR "prolonged exercise" OR "long-distance" OR marathon* OR ultramarathon* OR triathlon* OR running OR cycling OR bicycl* OR rowing):ti,ab,kw) AND ([mh Drinking] OR [mh "Water-Electrolyte Balance"] OR [mh Dehydration] OR [mh "Sodium, Dietary"] OR (hydrat* OR rehydrat* OR dehydrat* OR hypohydrat* OR "fluid intake" OR "fluid replacement" OR "fluid strategy" OR "fluid strategies" OR "drinking strategy" OR "drinking strategies" OR "ad libitum" OR "thirst-driven" OR "programmed drinking" OR "water ingestion" OR electrolyte* OR sodium):ti,ab,kw) AND (random* OR crossover OR "cross-over" OR counterbalanc*):ti,ab,kw |
| #5 Scopus | TITLE-ABS-KEY((endurance W/3 (exercise* OR train* OR sport*)) OR "prolonged exercise" OR "long-distance" OR marathon* OR ultramarathon* OR triathlon* OR running OR cycling OR bicycl* OR rowing) AND TITLE-ABS-KEY(hydrat* OR rehydrat* OR dehydrat* OR hypohydrat* OR "fluid intake" OR "fluid replacement" OR "fluid strategy" OR "fluid strategies" OR "drinking strategy" OR "drinking strategies" OR "ad libitum" OR "thirst-driven" OR "programmed drinking" OR "water ingestion" OR electrolyte* OR sodium) AND TITLE-ABS-KEY(random* OR crossover OR "cross-over" OR counterbalanc*) |
| #6 SPORTDiscus (EBSCOhost) | TX ((endurance N3 (exercise* OR train* OR sport*)) OR "prolonged exercise" OR "long-distance" OR marathon* OR ultramarathon* OR triathlon* OR running OR cycling OR bicycl* OR rowing) AND TX (hydrat* OR rehydrat* OR dehydrat* OR hypohydrat* OR "fluid intake" OR "fluid replacement" OR "fluid strategy" OR "fluid strategies" OR "drinking strategy" OR "drinking strategies" OR "ad libitum" OR "thirst-driven" OR "programmed drinking" OR "water ingestion" OR electrolyte* OR sodium) AND TX (random* OR crossover OR "cross-over" OR counterbalanc*) |

Reported coverage: database inception to 1 June 2026. The reconstructed strings are not authenticated records of the searches originally executed. Original execution histories, database-specific execution dates and counts were not retained. No separate comprehensive trial-registry or dissertation search, or reproducibly logged systematic forward-citation search, is claimed. No language or publication-year filter was applied to retrieval; conference abstracts were ineligible primary reports.

**Table S3. Reconciled eligibility record and reviewer-prompted re-audit.**

Panel A. All 69 excluded entries in the current 93-record eligibility log. The remaining 24 records are retained studies. Four previous inclusions were reclassified following source and criterion checks. Exclusion decisions below are not a list of every potentially relevant publication ever cited by a reviewer.

| **No.** | **Study** | **Short title** | **Source** | **Exclusion category** | **Reason for exclusion** | **DOI** |
| --- | --- | --- | --- | --- | --- | --- |
| 1 | (Conference) (2016) | Abstracts, Dec 2015 Int. Sports & Exercise Nutrition Conf. | Int J Sport Nutr Exerc Metab (Suppl) | Ineligible publication type | Conference abstract collection rather than a primary study. | Not available |
| 2 | (Conference) (2019) | Abstracts, Dec 2018 Int. Sports & Exercise Nutrition Conf. | Int J Sport Nutr Exerc Metab (Suppl) | Ineligible publication type | Conference abstract collection rather than a primary study. | 10.1123/ijsnem.2019-0057 |
| 3 | AbuMoh'd (2020) | Isotonic sports drink during exercise & recovery | Sport Mont | Carbohydrate manipulation and non-continuous exercise spanning recovery | Mixed within-exercise and post-exercise fluid provision; the performance test followed recovery, so the randomized continuous exposure was below 60 min, and the beverage manipulated carbohydrate and electrolytes together. | 10.26773/smj.200617 |
| 4 | Anastasiou (2009) | Sodium replacement & plasma Na drop (fluid matches loss) | J Athl Train | Exercise structure was not continuous endurance exercise | The protocol included resistance and intermittent segments and a change of exercise mode, so it did not meet the definition of continuous endurance exercise. | 10.4085/1062-6050-44.2.117 |
| 5 | Arnaoutis (2012) | Water ingestion vs mouth rinse in dehydrated subjects | Med Sci Sports Exerc | Exercise was not continuous and the randomized performance segment was shorter than 60 min | The randomized comparison occurred after a non-continuous pre-dehydration protocol of alternating exercise and rest, and the randomized time to exhaustion lasted about 18-22 min. | 10.1249/MSS.0b013e3182285776 |
| 6 | Backhouse (2007) | Water ingestion during prolonged exercise on affect | Appetite | Not randomized (counterbalanced order only) | The report described a counterbalanced design only, with no statement of random allocation. | 10.1016/j.appet.2006.08.064 |
| 7 | Bardis (2017) | Prescribed vs ad libitum drinking, cycling in heat | Med Sci Sports Exerc | Not randomized (counterbalanced order only) | The report described a counterbalanced order only, with no statement of random allocation. | 10.1249/MSS.0000000000001202 |
| 8 | Bergeron (2004) | Erythrocyte sickling during exercise & thermal stress | Clin J Sport Med | Randomized exercise exposure shorter than 60 min | The exercise bout lasted 45 min, below the 60 min threshold, in a small sample with sickle-cell trait. | Not available |
| 9 | Berkulo (2016) | Ad libitum drinking & performance, 40-km cycling TT in heat | Eur J Sport Sci | Not randomized (counterbalanced order only) | The report described a counterbalanced order only, with no statement of random allocation. | 10.1080/17461391.2015.1009495 |
| 10 | Che Muhamed (2019) | The Efficacy of Ingesting Water on Thermoregulatory Responses and Running Performance in a Warm-Humid Condition | Frontiers in Physiology | Non-randomized hydration contrast | The full Methods specify that no-fluid trials preceded fluid-ingestion trials; only environmental order was randomized, not the hydration contrast. | 10.3389/fphys.2019.00507 |
| 11 | Cheuvront & Haymes (2001) | Ad libitum fluid & thermoregulation, three environments | J Sports Sci | Drinking was not manipulated (environment was the independent variable) | All arms drank ad libitum and the independent variable was the environment, so no two eligible during-exercise hydration strategies were compared. | 10.1080/026404101753113796 |
| 12 | Cosgrove (2013) | Sodium supplementation has no effect on endurance performance during a cycling time-trial in cool conditions: a randomised cross-over trial | Journal of the International Society of Sports Nutrition | Unmatched fluid volume | Sodium supplementation was compared under ad libitum drinking with different achieved fluid volumes; the original-plan matched-fluid-volume requirement for a sodium contrast was not met. | 10.1186/1550-2783-10-30 |
| 13 | Cureton (2007) | Caffeinated sports drink: ergogenic effects | Int J Sport Nutr Exerc Metab | Carbohydrate or caffeine was the manipulated component | The manipulated components were carbohydrate and caffeine. | Not available |
| 14 | Daries (2000) | Fluid intake volume on 2-h running performance | Med Sci Sports Exerc | Fluid volume and carbohydrate intake varied together | Carbohydrate intake changed with fluid volume, so the fluid-dose effect could not be isolated. | 10.1097/00005768-200010000-00018 |
| 15 | Davis (2014) | 75% vs 150% between-bout fluid replacement, 10-km run | Eur J Sport Sci | Intervention was not during exercise (between-bout or overnight rehydration) | Fluid replacement occurred between the evening run and the following morning time trial, and the time trial itself lasted less than 60 min. | 10.1080/17461391.2014.894578 |
| 16 | De Melo-Marins (2018) | Personalized hydration on HR/skin temp & cycling capacity (heat) | Front Nutr | Randomized exercise exposure shorter than 60 min | The randomized time to exhaustion lasted about 37-38 min; a separate familiarization visit could not be added to the randomized exposure. | 10.3389/fnut.2018.00022 |
| 17 | Del Coso (2008) | Water vs sports drinks on anaerobic performance (heat) | Appl Physiol Nutr Metab | Target contrast confounded by beverage composition and outcome structure | Carbohydrate concentration, sodium and beverage type varied together and the primary performance outcome was anaerobic power. | 10.1139/H07-188 |
| 18 | Del Coso (2016) | Oral salt supplementation, half-ironman (RCT) | Scand J Med Sci Sports | Background fluid volume was not matched in the sodium comparison | Food and fluid were taken ad libitum during the race and the sodium group ingested about 0.4 L more fluid, so background fluid was not matched. | 10.1111/sms.12427 |
| 19 | Deshayes (2021) | Repeated hypohydration exposures & endurance performance | Nutrients | Chronic training-block hydration design | A four-week hydration-adaptation training-block design rather than an acute during-exercise strategy. | 10.3390/nu13124477 |
| 20 | Fan (2020) | ORS after exercise on fluid balance & endurance | Nutrients | Intervention was not during exercise (post-exercise rehydration) | The intervention was post-exercise rehydration and the beverage manipulated carbohydrate and sodium together. | 10.3390/nu12123826 |
| 21 | Funnell (2023) | Exercise-induced hypohydration impairs 3 km treadmill-running performance in temperate conditions | Journal of Sports Sciences | Non-continuous exercise | Repeated 6-min running bouts separated by programmed 1-min passive rests; does not meet the continuous-exercise criterion. | 10.1080/02640414.2023.2259728 |
| 22 | Ganio (2010) | Various CHO-electrolyte fluids on cycling & MVC | Int J Sport Nutr Exerc Metab | Carbohydrate or an unmatched active ingredient was the manipulated component | Beverages contained unmatched caffeine, carnitine or taurine, and the eligible contrast reduced to a carbohydrate comparison. | Not available |
| 23 | García-Berger (2020) | Skim milk vs isotonic drink before exercise, TT | J Int Soc Sports Nutr | Intervention was not during exercise (pre-exercise only) | Fluid was provided 3 h and 1.5 h before exercise, not during exercise. | 10.1186/s12970-020-00346-9 |
| 24 | González-Alonso (José) (1997) | Dehydration impairs cardiovascular function (hyperthermic) | J Appl Physiol | Mechanistic clamp design with intravenous volume manipulation | A water-perfused suit clamped skin temperature and blood volume was restored by infusion, so an oral hydration effect could not be isolated. | Not available |
| 25 | Hailes (2016) | Fluid volume & temperature (ice slurry) thermoregulation | Wilderness Environ Med | Beverage temperature or ice slurry was the manipulated component | The manipulated component was beverage temperature and physical state (ice slurry). | Not available |
| 26 | Hargreaves (1996) | Fluid ingestion on muscle metabolism, prolonged cycling | J Appl Physiol | Not randomized | No statement of random allocation; order was counterbalanced and the fluid volume was derived from a preceding no-fluid trial. | 10.1152/jappl.1996.80.1.363 |
| 27 | Hew-Butler (2006) | Sodium supplementation not required (Ironman) | Br J Sports Med | Field sodium trial without matched fluid or carbohydrate intake | Background beverage, carbohydrate and actual fluid volume were not matched in this field trial. | 10.1136/bjsm.2005.022418 |
| 28 | Hoffman (2018) | Ad libitum vs programmed drinking, 2-h running (temps) | Eur J Appl Physiol | No clean eligible hydration contrast | In one analysis all arms drank ad libitum and only the environment differed; in the other, the programmed-drinking arm also received glucose or whey hydrolysate. | 10.1007/s00421-018-3996-7 |
| 29 | Holmes (2011) | Exercise intensity on sweat rate and sweat sodium loss | J Sci Med Sport (Suppl, abstract) | Ineligible publication type | Conference abstract reporting an observational analysis with no during-exercise hydration intervention. | Not available |
| 30 | Huang (2020) | Low-osmolality CES on fluid loss & oxidative stress after EEE | Antioxidants | Intervention was not during exercise (before and after exercise) | The beverage was taken 1 h before and 1 h after exercise, and carbohydrate was the manipulated component. | 10.3390/antiox9040336 |
| 31 | Ito (2020) | Electrolyzed hydrogen water during endurance exercise | Temperature | Beverage with a special active property was the manipulated component | The manipulated component was the electrolyzed-hydrogen property of the water, not fluid volume or electrolyte dose. | 10.1080/23328940.2020.1742056 |
| 32 | Jeukendrup (1997) | CHO-E feedings improve 1-h TT cycling | Int J Sports Med | Carbohydrate was the manipulated component | Carbohydrate was the manipulated component at a matched fluid volume. | Not available |
| 33 | Kalpana (2013) | Sugarcane juice vs sports drink vs water, cycling | Ann Appl Sport Sci | Carbohydrate source or beverage type was the manipulated component | The manipulated component was the carbohydrate source or beverage type at a matched carbohydrate concentration. | Not available |
| 34 | Lambert (1992) | Fluid replacement after dehydration: carbonation & CHO | Int J Sports Med | Intervention was not during exercise (post-exercise rehydration) | Post-exercise rehydration with carbonation and carbohydrate manipulated; the performance test lasted 5 min. | Not available |
| 35 | Lau (2021) | ORS vs spring water on muscle cramp susceptibility | J Int Soc Sports Nutr | Randomized exercise exposure shorter than 60 min; outcome exploratory only | Downhill running of 40-60 min did not reliably reach the 60 min threshold and the primary outcome was exploratory cramp susceptibility. | 10.1186/s12970-021-00414-8 |
| 36 | Lee (2014) | Self-selecting fluid intake, high CHO, half-marathon | Int J Sports Med | Hydration strategy and nutrient-delivery format varied together | Fluid volume, drinking rule and nutrient-delivery format (drink versus gel plus electrolyte fluid) varied together. | 10.1055/s-0034-1375635 |
| 37 | Liu (Mi Dong) (2015) | Oligosaccharides in sports drink on performance | Adv J Food Sci Technol | Carbohydrate was the manipulated component | Oligosaccharide (carbohydrate) was the manipulated component and the cycling bout lasted about 47 min. | 10.19026/ajfst.8.1498 |
| 38 | Maresh (2004) | Hydration status on thirst/drinking during low-intensity exercise | J Appl Physiol | Exposure was pre-exercise hydration status, not a during-exercise strategy | The independent variable was pre-exercise hydration status; during-exercise drinking was observed rather than manipulated. | Not available |
| 39 | Maunder (2017) | Ad libitum ice slurry & cold fluid, cycling TT in heat | Int J Sports Physiol Perform | Beverage temperature or ice slurry was the manipulated component | The manipulated component was ice slurry versus cold fluid, that is, temperature and physical state. | 10.1123/ijspp.2015-0764 |
| 40 | McCartney (2019) | Post-exercise beverages with food on fluid recovery | Physiol Behav | Intervention was not during exercise (post-exercise rehydration) | Post-exercise beverage and food provision rather than a during-exercise strategy. | 10.1016/j.physbeh.2018.12.013 |
| 41 | McMurray (2006) | Timing of fluid intake, Olympic-distance triathlon | Int J Sport Nutr Exerc Metab | Not randomized (counterbalanced order only) | The report described a counterbalanced order only, with no statement of random allocation. | 10.1123/ijsnem.16.6.611 |
| 42 | Mears (2020) | Sodium alginate + pectin added to CHO-E solution | Appl Physiol Nutr Metab | Hydrogel additive was the manipulated component | The manipulated component was a sodium-alginate and pectin hydrogel additive at matched carbohydrate and electrolyte dose. | 10.1139/apnm-2019-0802 |
| 43 | Millard-Stafford (1992) | CHO-electrolyte replacement improves 40-km run | Med Sci Sports Exerc | Carbohydrate was the manipulated component | Carbohydrate was the manipulated component at a matched fluid volume. | Not available |
| 44 | Morris (2015) | Acute sodium before exercise → pre-exercise hyperhydration | Int J Sport Nutr Exerc Metab | Intervention was not during exercise (pre-exercise sodium loading) | Sodium was ingested during a 2 h pre-exercise hyperhydration period, not during exercise. | 10.1123/ijsnem.2014-0212 |
| 45 | Mündel (2006) | Drink temperature on fluid intake & endurance | Exp Physiol | Beverage temperature was the manipulated component | The manipulated component was beverage temperature (19 versus 4 degrees C). | 10.1113/expphysiol.2006.034223 |
| 46 | O'Brien & Rowlands (2011) | Fructose-maltodextrin ratio in CHO-electrolyte solution | Am J Physiol Gastrointest Liver Physiol | Carbohydrate was the manipulated component | The manipulated component was the fructose-to-maltodextrin ratio; sodium was a carrier only. | 10.1152/ajpgi.00419.2010 |
| 47 | Peters (2002) | Duodenal motility, run-bike-run: sports drink vs water | Eur J Gastroenterol Hepatol | Carbohydrate was the manipulated component | Carbohydrate was the manipulated component at a matched fluid volume. | Not available |
| 48 | Qin (2017) | Three sports drinks on substrate metabolism & performance | Nutrients | Carbohydrate or protein was the manipulated component | Carbohydrate concentration was matched and the manipulated components were carbohydrate molecular weight and protein. | 10.3390/nu9040377 |
| 49 | Roberts (2014) | Sports drink on exogenous CHO oxidation & performance | J Int Soc Sports Nutr | Carbohydrate was the manipulated component | Carbohydrate was the manipulated component and the primary outcome was exogenous carbohydrate oxidation. | 10.1186/1550-2783-11-8 |
| 50 | Rollo & Williams (2009) | CHO-E solution before & during 1-h run | Int J Sport Nutr Exerc Metab | Carbohydrate was the manipulated component | Carbohydrate was the manipulated component at a matched fluid volume. | Not available |
| 51 | Rollo (2012) | CHO-E drinking strategy (ad libitum/prescribed/none), 10-mile run | Int J Sport Nutr Exerc Metab | Fluid volume and carbohydrate intake varied together | Fluid volume and carbohydrate intake varied together across the no-drink, ad libitum and prescribed conditions. | 10.1123/ijsnem.22.5.338 |
| 52 | Rosales (2021) | Fluid delivery schedule & composition, physiologic strain (heat) | Wilderness Environ Med | Not randomized and exercise was not continuous | No statement of random allocation, and the 120 min task comprised 55 min walking, a 5 min standing rest and 55 min walking. | 10.1016/j.wem.2020.10.006 |
| 53 | Rowlands (2011) | Hypo/iso/hypertonic sports drinks on peak power | Int J Sport Nutr Exerc Metab | Carbohydrate concentration or beverage tonicity was the manipulated component | The manipulated components were beverage tonicity and carbohydrate concentration at a matched fluid volume. | Not available |
| 54 | Sanders (2001) | Sodium replacement and fluid shifts during prolonged exercise in humans | European Journal of Applied Physiology | Non-continuous exercise | Eight 25-min cycling bouts separated by programmed 5-min rests; does not meet the continuous-exercise criterion. | 10.1007/s004210000371 |
| 55 | Savoie (2015) | Intestinal temperature does not reflect rectal temperature | (measurement validation) | Ineligible publication type | Measurement-validation study comparing intestinal with rectal temperature rather than a randomized hydration trial. | Not available |
| 56 | Shaver (2018) | Drinking vs rinsing water, 15-km run (females) | Int J Exerc Sci | Mouth rinse was the only comparator | The only comparator was mouth rinsing, which is an excluded intervention, so no second eligible oral strategy was available. | Not available |
| 57 | Sims (2007) | Preexercise sodium loading — women in the heat | J Appl Physiol | Intervention was not during exercise (pre-exercise sodium loading) | Sodium loading occurred before exercise rather than during exercise. | 10.1152/japplphysiol.01203.2006 |
| 58 | Sims (2007) | Sodium loading — trained men in the heat | Med Sci Sports Exerc | Intervention was not during exercise (pre-exercise sodium loading) | Sodium loading occurred before exercise rather than during exercise. | 10.1249/01.mss.0000241639.97972.4a |
| 59 | Skotniczny (2026) | Core-perception coupling under different hydration status | J Hum Kinet | Counselling or correlational design | The intervention was structured hydration counselling influencing baseline hydration, and the analysis was correlational. | 10.5114/jhk/220099 |
| 60 | Snell (2010) | Non-caffeinated rehydration drinks on short-term performance | J Int Soc Sports Nutr | Intervention was not during exercise (post-exercise rehydration) | Rehydration beverages were taken during recovery and contained unmatched amino acids and vitamins. | 10.1186/1550-2783-7-28 |
| 61 | Sproule (1998) | No fluid vs CHO-E fluid & environment on running economy | Eur J Appl Physiol | No eligible randomized hydration contrast | The no-fluid condition was fixed as the first trial and was therefore not randomized; the subsequent randomized contrasts manipulated carbohydrate. | 10.1007/s004210050371 |
| 62 | Sun (2015) | CES enhances endurance capacity in active females | Nutrients | Carbohydrate was the manipulated component | Carbohydrate was the manipulated component at an approximately matched fluid volume. | 10.3390/nu7053739 |
| 63 | Twerenbold (2003) | Different sodium concentrations in replacement fluids (women) | Br J Sports Med | Sodium exposure confounded with environment across trials | The high-sodium, low-sodium and water trials took place in markedly different weather conditions, so sodium and environment could not be separated. | 10.1136/bjsm.37.4.300 |
| 64 | Vallier (2005) | Fluid ingestion on neuromuscular function, 180-min cycling | Br J Sports Med | Not randomized (no random sequence reported) | The report described a crossover design only, with no statement of random allocation or random order. | 10.1136/bjsm.2004.012393 |
| 65 | Van Nieuwenhoven (2005) | Two sports drinks & water on GI & 18-km run | Int J Sports Med | Carbohydrate or an unmatched active ingredient was the manipulated component | The manipulated components were carbohydrate and caffeine at a matched fluid volume. | 10.1055/s-2004-820931 |
| 66 | Vrijens & Rehrer (1999) | Sodium-free fluid decreases plasma Na (heat) | J Appl Physiol | Sodium and carbohydrate varied together | The randomized comparison was distilled water versus a beverage containing both 18 mmol/L sodium and 63 g/L carbohydrate, so carbohydrate was not matched. | 10.1152/jappl.1999.86.6.1847 |
| 67 | Wall (2015) | Blinded hydration status; dehydration & performance | Br J Sports Med | Non-oral (intravenous or reinfusion) fluid administration | Hydration status was manipulated by saline reinfusion, which is not oral administration. | 10.1136/bjsports-2013-092417 |
| 68 | Wells (1985) | 20-mile run under three fluid replacement treatments | Med Sci Sports Exerc | Carbohydrate or an unmatched active ingredient was the manipulated component | Water, an electrolyte-glucose beverage and a caffeinated beverage were compared at a matched volume, with no clean fluid-dose or sodium-dose manipulation. | Not available |
| 69 | Yeargin (2015) | Ad libitum fluid via self- or external administration | J Athl Train | Fluid-delivery mode in an intermittent team setting was the manipulated component | The manipulated component was the mode of fluid delivery in an intermittent team setting, and the exercise was not continuous. | 10.4085/1062-6050-49.3.76 |

Panel B. Reviewer-prompted examples and re-audit decisions. Reviewer examples that could not be fully verified are explicitly distinguished from the 69 completed exclusions; lack of a complete source is not evidence of ineligibility.

| **Candidate report** | **Final eligibility decision** | **Randomization audit** | **Exercise duration and structure** | **Basis for the decision** |
| --- | --- | --- | --- | --- |
| Arnaoutis (2012) | Excluded | Explicit statement of random allocation | Pre-dehydration of alternating 25 min exercise and 5 min rest over 2 h, followed by a randomized time to exhaustion of about 18-22 min | The pre-dehydration phase alternated exercise with rest and the randomized time to exhaustion was shorter than 60 min. |
| Backhouse (2007) | Excluded | Counterbalanced order only, no statement of random allocation | 90 min continuous running | No statement of random allocation. |
| Backx (2003) | Included | Explicit statement of random allocation | 1 h self-paced time trial | Randomized 1 h time trial with 900, 450 and 120 mL during exercise at matched total carbohydrate and sodium; added to the review. |
| Bardis (2017) | Excluded | Counterbalanced order only, no statement of random allocation | 30 km criterium-like cycling | Counterbalanced order only, with no statement of random allocation. |
| De Melo-Marins (2018) | Excluded | Explicit statement of random allocation | Randomized time to exhaustion of about 37-38 min | The randomized exercise exposure was about 37-38 min; the familiarization visit could not be added to it. |
| Del Coso (2016) | Excluded | Explicit statement of random allocation (parallel groups) | Half-ironman triathlon, longer than 60 min | Background fluid volume was not matched in the sodium comparison. |
| González-Alonso (1995) | Included | Explicit statement of random allocation | 2 h continuous cycling in the heat | Randomized continuous 2 h cycling in the heat with 3.60 L versus 0.20 L at identical total carbohydrate and electrolyte intake; added to the review. |
| Hoffman (2018) | Excluded | Explicit statement of random allocation | 2 h running | One analysis varied only the environment; the other confounded programmed drinking with glucose or whey hydrolysate. |
| Lee (2014) | Excluded | Explicit statement of random allocation | Half-marathon of about 110 min | Fluid volume and drinking rule varied together with the nutrient-delivery format. |
| Montain & Coyle (1992a) | Included; oral comparison only | Explicit random allocation of study conditions | 2 h continuous cycling in the heat | Oral fluid versus no-fluid contrast retained as a combined drinking strategy with carbohydrate co-ingestion, not a carbohydrate-matched isolated water effect. Intravenous dextran arm excluded. |
| Rosales (2021) | Excluded | No statement of random allocation | 55 min walking, a 5 min standing rest, then 55 min walking | No statement of random allocation and the protocol included a programmed 5 min rest. |
| Sproule (1998) | Excluded | Partly randomized; the target contrast was not randomized | 60 min running | The no-fluid condition was not randomized and the subsequent randomized contrasts manipulated carbohydrate. |
| Vallier (2005) | Excluded | No statement of random allocation | 180 min cycling | Crossover design only, with no statement of random allocation. |
| Vrijens & Rehrer (1999) | Excluded | Explicit statement of random allocation | 3 h cycling in the heat | Sodium and carbohydrate were changed together. |
| Montain & Coyle (1992b) | Included; narrative only | Explicit statement of random allocation | 2 h continuous cycling in the heat | Randomized graded-dose study retained in the current eligibility record. Unverified numerical entries were not used in any synthesis. |
| Che Muhamed (2019) | Excluded | Environmental order randomized; hydration order fixed | See source protocol | The full Methods specify that no-fluid trials preceded fluid-ingestion trials; only environmental order was randomized, not the hydration contrast. |
| Funnell (2023) | Excluded | Randomized crossover | See source protocol | Repeated 6-min running bouts separated by programmed 1-min passive rests; does not meet the continuous-exercise criterion. |
| Sanders (2001) | Excluded | Randomized crossover | See source protocol | Eight 25-min cycling bouts separated by programmed 5-min rests; does not meet the continuous-exercise criterion. |
| Cosgrove (2013) | Excluded | Randomized crossover | See source protocol | Sodium supplementation was compared under ad libitum drinking with different achieved fluid volumes; the original-plan matched-fluid-volume requirement for a sodium contrast was not met. |
| Below (1995) | Not quantitatively synthesized | Factorial design includes carbohydrate-matched contrasts | 50-min preload followed by a performance test | The factorial design includes carbohydrate-matched contrasts. Exact eligibility at the 60-min threshold was not established from the available report information; no full-text exclusion is claimed. |
| Fritzsche (2000) | Not quantitatively synthesized | Randomized four-condition design includes water-only contrasts | Repeated maximal neuromuscular-power tests within prolonged cycling | Not a time-trial or time-to-exhaustion performance endpoint. Full-text exercise structure and eligible physiological extraction could not be completed; no carbohydrate-based exclusion is claimed. |
| Candas series / Costill (1970) | Not quantitatively synthesized | Not verified from a complete report | Not verified from a complete report | The available information did not permit a completed full-text eligibility judgment. These examples remain unclassified and are acknowledged as a coverage limitation. |

**Table S4. Intervention and comparator details.**

| **Study** | **Intervention or exposure** | **Comparator** | **Ambient conditions** | **Beverage temperature** | **Timing of ingestion** | **Fluid intake relative to time or body mass** | **Notes** |
| --- | --- | --- | --- | --- | --- | --- | --- |
| Backx et al. (2003) | High fluid (900 mL) or moderate fluid (450 mL) consumed in three equal boluses at 15, 30 and 45 min of a 1 h time trial | Low fluid (120 mL) in three equal boluses at the same times | 20 °C, 70% relative humidity; environmental chamber | Not reported | 15, 30 and 45 min | Approximately 900, 450 and 120 mL/h during exercise (11.7, 5.9 and 1.6 mL/kg/h) | A 6 mL/kg pre-exercise carbohydrate bolus was given in every trial; added maltodextrin and sodium citrate kept total carbohydrate (57.6 g) and sodium (0.45 g) constant, so beverage osmolality differed between trials (280, 390 and 780 mOsm/kg). The non-randomized familiarization trial was excluded. |
| Claveau et al. (2022) | Ad libitum water intake | Water intake according to thirst sensation | 33 °C, 40% relative humidity | Not reported | Available throughout | Approximately 0.9 L/h | Single-study comparison domain; not meta-analysed. |
| Costa et al. (2019) | Euhydration through programmed water provision during 2 h running | Total water restriction producing hypohydration | 25 °C, 46% relative humidity | Not reported | Programmed through exercise | Not reported | — |
| Dion et al. (2013) | Programmed fluid intake above thirst (approximately 1,380 mL/h) | Thirst-driven fluid intake (approximately 384 mL/h) | 30 °C, 42% relative humidity | Not reported | Through the half-marathon | 1,380 ± 320 vs 384 ± 180 mL/h | — |
| Dugas et al. (2009) | Water replacing 0%, 33%, 66% or 100% of sweat loss, plus an ad libitum condition | No fluid / 0% replacement as the principal reference | 33 °C, environmental chamber with convective cooling | Approximately 20 °C | Through the 80 km time trial | 0, 426 ± 69, 865 ± 164 and 1,324 ± 180 mL/h | The WET mouth-rinse arm was excluded. Achieved replacement (0, 32.4, 59.9 and 94.6%) differed from the target. |
| Fallowfield et al. (1996) | Water bolus before exercise plus serial water feedings during exercise | No fluid | 20 °C | Not reported | Pre-exercise bolus plus serial feedings | 3 mL/kg pre-exercise plus 2 mL/kg serially; 929 ± 96 mL in total | Author-reported paired precision was available for time to exhaustion. |
| González-Alonso et al. (1995) | 3.60 L of carbohydrate-electrolyte fluid replacing 95.1 ± 3.1% of sweat losses | 0.20 L of fluid with the same total carbohydrate/electrolyte dose, replacing 2.7 ± 1.6% of sweat losses | 35.4 ± 0.2 °C, 48 ± 2% relative humidity, wind approximately 2 m/s | 38 °C | Euhydration trial at 20, 35, 50, 65, 80 and 95 min; dehydration trial at 30, 60 and 90 min | Approximately 1.80 vs 0.10 L/h | Total carbohydrate and electrolyte intake was identical in the two trials (70 g of sports-drink powder containing 41 g sucrose, 28 g glucose, 518 mg sodium, 518 mg chloride and 118 mg potassium), so fluid volume was the only manipulated component. |
| Jeker et al. (2022) | Programmed water intake aiming to limit body mass loss to approximately 1% | Thirst-driven water intake | 30 °C, 35% relative humidity | Not reported | Through 5 h of cycling | Approximately 1.3 vs 1.0 L/h | — |
| Juett et al. (2024) | Water replacing 100% of body mass losses | Minimal water (75–100 mL) to induce approximately 3% body mass loss | Approximately 35 °C, approximately 50% relative humidity | Not reported | Through the cycling bout | Not reported for the exercise period alone | — |
| Kay et al. (2003) | Fluid sufficient to prevent body mass loss in moderate and warm-humid conditions | No fluid within each environment | Moderate 21 °C; warm-humid 31 °C | Not reported | Through the 60 min bout | 1.3 ± 0.4 L/h (moderate) and 1.5 ± 0.4 L/h (warm) | Condition-specific contrasts were averaged within study. Temperature was measured rectally. |
| Lambert et al. (2008) | Water placebo, 3 mL/kg every 10 min | No fluid | 24.4 ± 0.04 °C; 32.7 ± 0.55% relative humidity | Not reported | Every 10 min | Approximately 18 mL/kg/h | Rest and 4% glucose-solution arms were excluded. Temperature was measured rectally. |
| Lopez et al. (2016) | Individualized water-replacement protocol | Ad libitum water intake | Outdoor trail race, wet-bulb globe temperature 28.3 ± 1.9 °C | Not reported | At aid stations | 1,700 ± 629 vs 749 ± 416 mL over the race | Achieved replacement 64 ± 16% vs 30 ± 14%. |
| Maughan et al. (1996) | Water, 100 mL every 10 min | No drink | 21 ± 1 °C | Not reported | Every 10 min | 600 mL/h | Isotonic and hypotonic carbohydrate-electrolyte arms were excluded. |
| McConell et al. (1997) | Water replacing 50% or 100% of estimated fluid losses | No fluid | 21.3 ± 0.3 °C; 43 ± 2% relative humidity | Room temperature | Six equal portions at 0, 20, 40, 60, 80 and 100 min | 0, 0.58 and 1.16 L/h | Achieved replacement 0, 55.3 ± 2.0 and 113.8 ± 4.6% exceeded the 50% and 100% targets. |
| Montain et al. (1992a) | Carbohydrate-electrolyte fluid replacing approximately 80% of sweat loss | No fluid | 33 °C | 37 °C | 0, 20, 35, 50, 65, 80 and 95 min; first two portions each 20% and last five each 12% of total | Approximately 1.2 L/h | The intravenous blood-volume-expansion arm was excluded. The oral condition used a carbohydrate-electrolyte solution; this is a combined drinking strategy, not a carbohydrate-matched water effect. Participant overlap with Montain et al. (1992b) cannot be excluded. |
| Montain et al. (1992b) | Numerical source verification unavailable; narrative only. | Numerical source verification unavailable; narrative only. | Numerical source verification unavailable; narrative only. | Numerical source verification unavailable; narrative only. | Numerical source verification unavailable; narrative only. | Numerical source verification unavailable; narrative only. | Numerical source verification unavailable; narrative only. |
| Montain et al. (1993) | The same total carbohydrate-electrolyte fluid consumed at exercise onset, 40 min, 80 min, or distributed throughout | Fluid at exercise onset as reference | 33 °C | Not reported | 0, 40, 80 min or distributed | 1,173 ± 44 mL in every condition | All timing arms used the same solution and total volume; only ingestion timing differed. Single-study comparison domain. |
| Nassis et al. (2002) | Water replacing approximately 60% of fluid losses during cycling or running | No fluid within each exercise mode | 23.8 ± 0.3 °C; 58.8 ± 1.4% relative humidity | Room-temperature tap water | 6.5 mL/kg after warm-up; further equal portions at 15, 30, 50 and 70 min | 1,165 ± 55 mL (cycling); 1,365 ± 108 mL (running), including the post-warm-up portion | Condition-specific contrasts were averaged within study. |
| Perreault-Brière et al. (2019) | Thirst-driven or programmed water intake | No fluid | 30 °C, 50% relative humidity | Not reported | Through the 1 h time trial | 1,606 ± 125 vs 565 ± 178 mL/h | Contributes to two comparison domains through different contrasts. |
| Robinson et al. (1995) | Artificially sweetened water replacing approximately 85% of fluid losses | No fluid | 20 °C, 60% relative humidity | Not reported | 8 mL/kg at warm-up and five equal aliquots at 0, 10, 20, 30 and 40 min | Approximately 1.49 L/h | — |
| Sage et al. (2026) | Water replacing 80% of sweat losses with serum sodium targeted; sodium intake individualized | Water replacing 60% of sweat losses with serum sodium targeted; sodium intake individualized | 32 °C, 50% relative humidity | Not reported | Through the 180 min preload and the time trial | 1.125 ± 0.269 vs 0.844 ± 0.202 L/h during the preload | Sodium exposure was matched between fluid-replacement conditions. |
| Sanders et al. (1999) | 1.2 L water or 1.2 L saline containing 100 mmol/L NaCl | No fluid; water also serves as the sodium-isolating comparator | 32 °C | Not reported | Through the 90 min bout | 0.8 L/h | Contributes to two comparison domains through different contrasts. |
| Walsh et al. (1994) | 20 mmol/L NaCl solution: 400 mL before and 120 mL every 10 min during the first 50 min | No fluid | 32 °C, 60% relative humidity | Not reported | Pre-exercise bolus plus every 10 min | Approximately 720 mL/h during the first 50 min | — |
| Wijering et al. (2023) | 6% carbohydrate beverage with 60 mmol/L sodium | Matched 6% carbohydrate beverage with 21 mmol/L sodium | 34 °C, 65% relative humidity | Not reported | Through up to 3 h of cycling | 1.14 ± 0.21 vs 1.12 ± 0.19 L/h | Both sodium conditions used the same carbohydrate beverage and matched fluid replacement; sodium concentration was the manipulated component. |

Values are those reported in the extracted source, except rates explicitly calculated from total volume and duration. “Not reported” is not a value of zero. Montain (1992b) remains narrative only. Sage used individualized sodium supplementation to target comparable serum sodium; sodium intake was not identical. The primary blood-analysis sample for Sage is nine participants.

**Table S5. Post hoc outcome-selection hierarchy and evidence outside the pooled models.**

| **Decision** | **Operational rule** |
| --- | --- |
| Performance | Separate TT/fixed-duration and TTE constructs; within TT prefer completion time, distance, mean power/work, then equivalent speed/pace. Capped protocol duration is descriptive only. |
| Core temperature | Rectal before esophageal before other core measures; do not substitute mixed mean body temperature. Site restrictions and alternative extracted sites are tested. |
| Timepoint/data form | Exclude baseline-only values. Prefer final post-exposure values when comparable alternatives exist; retain end-preload measurement when no later same-outcome result is available. Fluid-intake-corrected mass loss is not pooled with net body-mass deficit. |
| Several conditions/doses | Average eligible environmental/mode contrasts within a study with covariance; use the two highest eligible doses for a dose contrast and highest versus zero/minimal for hydration versus restriction. |
| Evidence destination | Pool only ≥2 independent studies with compatible precision. Retain single-study/point-only/alternative evidence; never manufacture precision for a point estimate. |

Panel A. Nonpooled contrast records. Full numerical inputs, including every extracted arm and reported-effect record, are linked in outputs/outcome_selection_audit.csv. “Not selected” denotes the documented hierarchy or an incompatible endpoint, not absence of a reported result.

| **ID** | **Study** | **Outcome** | **Timepoint** | **Effect (95% CI)** | **Evidence destination / reason** |
| --- | --- | --- | --- | --- | --- |
| C004 | Maughan et al. (1996) | Time-to-exhaustion performance | To exhaustion | 16.6%; precision unavailable | Compatible precision unavailable. |
| C029 | Fallowfield et al. (1996) | Fluid-intake-corrected body mass loss (%) | Post exercise | 0.70 (0.31 to 1.09) percentage points | Fluid intake was added back in this measure; descriptive only, not pooled with net body-mass deficit. |
| C035 | Walsh et al. (1994) | Body mass loss (%) | After 60 min | -1.60 percentage points; precision unavailable | Compatible precision unavailable. |
| C040 | Montain et al. (1992a) | Plasma volume change (%) | 5–120 min change | 3.00 percentage points; precision unavailable | Compatible precision unavailable. |
| C043 | Walsh et al. (1994) | Plasma volume change (%) | 60 min | 4.50 percentage points; precision unavailable | Compatible precision unavailable. |
| C047 | Perreault-Brière et al. (2019) | Abdominal discomfort | Mean during exercise | 0.40 (-0.11 to 0.91) AU | Single-study or supporting direct result; not pooled. |
| C051 | Walsh et al. (1994) | Plasma sodium concentration | Mean first hour | -1.00 mmol/L; precision unavailable | Compatible precision unavailable. |
| C052 | Perreault-Brière et al. (2019) | Plasma sodium change | Pre-to-post change | 0.20 (-7.13 to 7.53) mmol/L | Single-study or supporting direct result; not pooled. |
| C059 | Walsh et al. (1994) | Urine volume (L) | 60 min | 0.01 L; precision unavailable | Compatible precision unavailable. |
| C061 | Fallowfield et al. (1996) | Whole-body sweat rate | Exercise bout | -0.01 L/h; precision unavailable | Compatible precision unavailable. |
| C074 | Dugas et al. (2009) | Fluid intake rate (L/h) | 80 km total | 1.32 (1.18 to 1.47) L/h | Single-study or supporting direct result; not pooled. |
| C078 | Montain et al. (1992a) | Body mass change (kg) | Post 2 h | 2.18 kg; precision unavailable | Compatible precision unavailable. |
| C081 | Robinson et al. (1995) | Body mass change rate | Post exercise | 1.05 (0.78 to 1.32) kg/h | Single-study or supporting direct result; not pooled. |
| C082 | Costa et al. (2019) | Breath hydrogen AUC | 3 h response | -609.00 (-1160.01 to -57.99) ppm·3h | Single-study or supporting direct result; not pooled. |
| C083 | Nassis et al. (2002) | Cardiac output change | 85 min | 1.70 (0.80 to 2.60) L/min | Single-study or supporting direct result; not pooled. |
| C084 | Sanders et al. (1999) | Fluid balance | Trial total | 1.30 (1.10 to 1.50) L | Single-study or supporting direct result; not pooled. |
| C085 | Robinson et al. (1995) | Fluid retained | Trial total | 1.02 (0.78 to 1.26) L | Single-study or supporting direct result; not pooled. |
| C086 | Costa et al. (2019) | GI symptom incidence | During/post exercise | -18.00 %; precision unavailable | Compatible precision unavailable. |
| C087 | Robinson et al. (1995) | Gastric residual volume | Post exercise | 0.27 (0.11 to 0.43) L | Single-study or supporting direct result; not pooled. |
| C088 | Costa et al. (2019) | I-FABP change | Pre-to-post change | -168.00 pg/mL; precision unavailable | Compatible precision unavailable. |
| C089 | Nassis et al. (2002) | Local sweat rate | 90 min | -0.01 (-0.09 to 0.06) mg/cm²/min | Single-study or supporting direct result; not pooled. |
| C093 | Walsh et al. (1994) | Rating of perceived exertion (CR10) | 60 min | -0.80 (-1.38 to -0.22) AU | Single-study or supporting direct result; not pooled. |
| C094 | Nassis et al. (2002) | Skin blood flow | 90 min | 18.20 (1.64 to 34.76) % baseline | Single-study or supporting direct result; not pooled. |
| C095 | Sanders et al. (1999) | Sodium balance | Trial total | -4.00 (-19.31 to 11.31) mEq | Single-study or supporting direct result; not pooled. |
| C096 | Nassis et al. (2002) | Stroke volume change | 85 min | 12.00 (5.26 to 18.74) mL/beat | Single-study or supporting direct result; not pooled. |
| C100 | Walsh et al. (1994) | Sweat loss | 60 min | 0.00 L; precision unavailable | Compatible precision unavailable. |
| C104 | Perreault-Brière et al. (2019) | Thirst sensation | End exercise | -3.30 (-4.59 to -2.01) AU | Single-study or supporting direct result; not pooled. |
| C107 | Lopez et al. (2016) | Time-trial/fixed-duration performance | 20 km total | -0.5%; precision unavailable | Compatible precision unavailable. |
| C118 | Perreault-Brière et al. (2019) | Plasma volume change (%) | Post exercise | 2.70 (-2.57 to 7.97) percentage points | Single-study or supporting direct result; not pooled. |
| C121 | Perreault-Brière et al. (2019) | Plasma sodium change | Pre-to-post change | -3.90 (-11.77 to 3.97) mmol/L | Single-study or supporting direct result; not pooled. |
| C131 | Dion et al. (2013) | Fluid intake rate (L/h) | Half-marathon | 1.00 (0.82 to 1.17) L/h | Single-study or supporting direct result; not pooled. |
| C132 | Jeker et al. (2022) | Fluid balance | Trial total | 0.69 (0.16 to 1.22) L | Single-study or supporting direct result; not pooled. |
| C133 | Dion et al. (2013) | Mean body temperature | Mean during race | -0.40 (-0.61 to -0.19) °C | Single-study or supporting direct result; not pooled. |
| C134 | Jeker et al. (2022) | Sodium intake rate | Trial total | -1.00 (-27.74 to 25.74) mg/h | Single-study or supporting direct result; not pooled. |
| C138 | Lopez et al. (2016) | Sweat loss replaced | 20 km total | 34.00 (25.79 to 42.21) % | Single-study or supporting direct result; not pooled. |
| C139 | Perreault-Brière et al. (2019) | Thirst sensation | End exercise | -0.80 (-1.79 to 0.19) AU | Single-study or supporting direct result; not pooled. |
| C140 | Lopez et al. (2016) | Urine osmolality | Pre race | 45.00 (-65.86 to 155.86) mOsm/kg | Baseline measurement precedes the exercise intervention; not used as an intervention effect. |
| C142 | McConell et al. (1997) | Time-to-exhaustion performance | After 2 h preload | 32.3% (-37.2% to 178.4%) | Single-study or supporting direct result; not pooled. |
| C144 | Sage et al. (2026) | Mean power output (alternative measure) | Time trial | 22.00 (4.00 to 39.00) W | Additional author-reported outcome or timepoint retained for narrative synthesis; not entered in the model-defined single-contribution meta-analysis. |
| C148 | Sage et al. (2026) | Body mass loss (%) | End 180 min preload | -1.00 (-1.30 to -0.70) percentage points | Additional author-reported outcome or timepoint retained for narrative synthesis; not entered in the model-defined single-contribution meta-analysis. |
| C155 | Dugas et al. (2009) | Whole-body sweat rate | 80 km total | -0.25 (-0.34 to -0.16) L/h | Single-study or supporting direct result; not pooled. |
| C160 | McConell et al. (1997) | Body mass change (kg) | Post 2 h | 1.01 (0.86 to 1.16) kg | Single-study or supporting direct result; not pooled. |
| C165 | Sage et al. (2026) | Total body water | End 180 min preload | 1.00 (0.70 to 1.40) L | Single-study or supporting direct result; not pooled. |
| C166 | Sage et al. (2026) | Urine sodium loss | Trial total | 7.41 (1.69 to 13.13) mmol | Single-study or supporting direct result; not pooled. |
| C167 | Wijering et al. (2023) | Capped exercise duration | Exercise duration | 0.00 (-4.84 to 4.84) min | Descriptive protocol-completion measure capped at 180 min and influenced by safety stopping rules; not a time trial or uncensored time-to-exhaustion endpoint. |
| C168 | Sanders et al. (1999) | Heart rate | 90 min | -2.00 (-5.39 to 1.39) beats/min | Single-study or supporting direct result; not pooled. |
| C169 | Wijering et al. (2023) | Body mass loss (%) | Post exercise | -0.23 (-0.48 to 0.02) percentage points | Single-study or supporting direct result; not pooled. |
| C172 | Sanders et al. (1999) | Plasma sodium concentration | 90 min | 4.00 (2.04 to 5.96) mmol/L | Single-study or supporting direct result; not pooled. |
| C173 | Wijering et al. (2023) | Plasma sodium change | Baseline to final | 2.30 (0.02 to 4.52) mmol/L | Single-study or supporting direct result; not pooled. |
| C174 | Wijering et al. (2023) | Whole-body sweat rate | Trial | 0.01 (-0.14 to 0.16) L/h | Single-study or supporting direct result; not pooled. |
| C175 | Wijering et al. (2023) | Fluid intake (L) | Trial total | 0.06 (-0.31 to 0.43) L | Single-study or supporting direct result; not pooled. |
| C176 | Wijering et al. (2023) | Fluid intake rate (L/h) | Trial | 0.02 (-0.10 to 0.14) L/h | Single-study or supporting direct result; not pooled. |
| C177 | Wijering et al. (2023) | Body mass change (kg) | Post exercise | 0.18 (-0.01 to 0.37) kg | Single-study or supporting direct result; not pooled. |
| C178 | Sanders et al. (1999) | Fluid balance | Trial total | -0.20 (-0.40 to -0.00) L | Single-study or supporting direct result; not pooled. |
| C179 | Sanders et al. (1999) | Sodium balance | Trial total | 97.00 (79.36 to 114.64) mEq | Single-study or supporting direct result; not pooled. |
| C180 | Wijering et al. (2023) | Sodium intake | Trial total | 132.00 (110.99 to 153.01) mmol | Single-study or supporting direct result; not pooled. |
| C181 | Wijering et al. (2023) | Sweat loss | Trial total | 0.03 (-0.43 to 0.49) L | Single-study or supporting direct result; not pooled. |
| C182 | Montain et al. (1993) | Core temperature | 140 min | -0.10 (-0.30 to 0.10) °C | Single-study or supporting direct result; not pooled. |
| C183 | Montain et al. (1993) | Heart rate | 140 min | 1.00 (-4.88 to 6.88) beats/min | Single-study or supporting direct result; not pooled. |
| C184 | Montain et al. (1993) | Fluid intake (L) | 140 min total | 0.00 (-0.09 to 0.09) L | Single-study or supporting direct result; not pooled. |
| C185 | Montain et al. (1993) | Sweat loss replaced | 140 min total | 0.00 %; precision unavailable | Compatible precision unavailable. |
| C195 | Backx et al. (2003) | Abdominal discomfort | 60 min | 1.80 (1.14 to 2.46) AU | Single-study or supporting direct result; not pooled. |
| C199 | Backx et al. (2003) | Heart rate | Mean during exercise | 7.00 (1.12 to 12.88) beats/min | Single-study or supporting direct result; not pooled. |
| C201 | Claveau et al. (2022) | Fluid intake (L) | 120 km total | 0.10 (-0.42 to 0.63) L | Single-study or supporting direct result; not pooled. |
| C202 | Claveau et al. (2022) | Sweat loss replaced | 120 km total | 4.00 (-4.79 to 12.79) percentage points | Single-study or supporting direct result; not pooled. |
| C203 | Claveau et al. (2022) | Body mass loss (%) | Post exercise | -0.30 (-0.96 to 0.36) percentage points | Single-study or supporting direct result; not pooled. |
| C204 | Claveau et al. (2022) | Urine volume (L) | Post exercise | 0.02 (-0.05 to 0.10) L | Single-study or supporting direct result; not pooled. |
| C205 | Claveau et al. (2022) | Core temperature | Mean during exercise | -0.20 (-0.61 to 0.21) °C | Single-study or supporting direct result; not pooled. |
| C206 | Claveau et al. (2022) | Heart rate | Mean during exercise | -5.00 (-18.12 to 8.12) beats/min | Single-study or supporting direct result; not pooled. |
| C207 | Claveau et al. (2022) | Rating of perceived exertion (Borg 6-20) | Mean during exercise | -1.00 (-2.64 to 0.64) AU | Single-study or supporting direct result; not pooled. |
| C208 | Claveau et al. (2022) | Urine osmolality | Pre-to-post change | 227.00 (73.61 to 380.39) mOsm/kg | Single-study or supporting direct result; not pooled. |
| C209 | Claveau et al. (2022) | Urine specific gravity | Pre-to-post change | 0.01 (0.00 to 0.01) AU | Single-study or supporting direct result; not pooled. |

Panel B. Montain and Coyle (1992b): retained as narrative evidence from the published study summary; all unverified numerical entries were removed. Its n=8 is included in the study-reported participant total, not in any pooled sample. Below, Fritzsche, the Candas examples and Costill (1970) could not be fully assessed from complete sources and received no quantitative weight.

**Table S6. Study-specific paired-data calculations.**

One row per numerical contrast. Reported paired intervals take precedence where available. For reconstruction, SD=SE×√n; paired MD variance=(SD₁²+SD₀²−2rSD₁SD₀)/n. Log-ratio variance uses the corresponding delta method. Multiple conditions are averaged with covariance ρ√(vᵢvⱼ). Post hoc primary assumptions: r=.50 and ρ=.50. The full source IDs and calculations are in outputs/analysis_contrasts.csv.

| **ID** | **Study** | **Outcome** | **N** | **Precision source** | **Within-participant correlation** | **Variance** | **SE** |
| --- | --- | --- | --- | --- | --- | --- | --- |
| C001 | Dugas et al. (2009) | Time-trial/fixed-duration performance | 6 | Reconstructed from marginal dispersions | 0.50 assumed | 0.0003798791 | 0.0194905 |
| C002 | Fallowfield et al. (1996) | Time-to-exhaustion performance | 8 | Author-reported paired precision | 0.940 | 0.001896423 | 0.0435479 |
| C003 | Kay et al. (2003) | Time-trial/fixed-duration performance | 7 | Reconstructed from marginal dispersions | 0.50 assumed | 0.003342664 | 0.0578158 |
| C005 | McConell et al. (1997) | Time-to-exhaustion performance | 7 | Reconstructed from marginal dispersions | 0.50 assumed | 0.1484017 | 0.385229 |
| C006 | Perreault-Brière et al. (2019) | Time-trial/fixed-duration performance | 9 | Reconstructed from marginal dispersions | 0.50 assumed | 0.000332999 | 0.0182483 |
| C007 | Robinson et al. (1995) | Time-trial/fixed-duration performance | 8 | Reconstructed from marginal dispersions | 0.50 assumed | 0.0002208503 | 0.014861 |
| C008 | Walsh et al. (1994) | Time-to-exhaustion performance | 6 | Reconstructed from marginal dispersions | 0.50 assumed | 0.03128597 | 0.176878 |
| C009 | Dugas et al. (2009) | Core temperature | 6 | Reconstructed from marginal dispersions | 0.50 assumed | 0.02666667 | 0.163299 |
| C010 | Fallowfield et al. (1996) | Core temperature | 8 | Reconstructed from marginal dispersions | 0.50 assumed | 0.12 | 0.34641 |
| C011 | Kay et al. (2003) | Core temperature | 7 | Reconstructed from marginal dispersions | 0.50 assumed | 0.01804383 | 0.134327 |
| C012 | Lambert et al. (2008) | Core temperature | 20 | Reconstructed from marginal dispersions | 0.50 assumed | 0.0125 | 0.111803 |
| C013 | Montain et al. (1992a) | Core temperature | 7 | Reconstructed from marginal dispersions | 0.50 assumed | 0.01 | 0.1 |
| C014 | Nassis et al. (2002) | Core temperature | 11 | Reconstructed from marginal dispersions | 0.50 assumed | 0.0175 | 0.132288 |
| C015 | Robinson et al. (1995) | Core temperature | 8 | Reconstructed from marginal dispersions | 0.50 assumed | 0.04 | 0.2 |
| C016 | Walsh et al. (1994) | Core temperature | 6 | Reconstructed from marginal dispersions | 0.50 assumed | 0.02666667 | 0.163299 |
| C017 | Costa et al. (2019) | Heart rate | 11 | Reconstructed from marginal dispersions | 0.50 assumed | 32.28521 | 5.68201 |
| C018 | Fallowfield et al. (1996) | Heart rate | 8 | Reconstructed from marginal dispersions | 0.50 assumed | 48 | 6.9282 |
| C019 | Juett et al. (2024) | Heart rate | 14 | Reconstructed from marginal dispersions | 0.50 assumed | 7.928571 | 2.81577 |
| C020 | Kay et al. (2003) | Heart rate | 7 | Reconstructed from marginal dispersions | 0.50 assumed | 13.05368 | 3.61299 |
| C021 | Lambert et al. (2008) | Heart rate | 20 | Reconstructed from marginal dispersions | 0.50 assumed | 4.2 | 2.04939 |
| C022 | Maughan et al. (1996) | Heart rate | 12 | Reconstructed from marginal dispersions | 0.50 assumed | 117 | 10.8167 |
| C023 | Montain et al. (1992a) | Heart rate | 7 | Reconstructed from marginal dispersions | 0.50 assumed | 3 | 1.73205 |
| C024 | Perreault-Brière et al. (2019) | Heart rate | 9 | Reconstructed from marginal dispersions | 0.50 assumed | 4.111111 | 2.02759 |
| C025 | Robinson et al. (1995) | Heart rate | 8 | Reconstructed from marginal dispersions | 0.50 assumed | 1.09 | 1.04403 |
| C026 | Sanders et al. (1999) | Heart rate | 6 | Reconstructed from marginal dispersions | 0.50 assumed | 4 | 2 |
| C027 | Walsh et al. (1994) | Heart rate | 6 | Reconstructed from marginal dispersions | 0.50 assumed | 36.16667 | 6.01387 |
| C028 | Costa et al. (2019) | Body mass loss (%) | 11 | Reconstructed from marginal dispersions | 0.50 assumed | 0.04756364 | 0.218091 |
| C029 | Fallowfield et al. (1996) | Fluid-intake-corrected body mass loss (%) | 8 | Reconstructed from marginal dispersions | 0.50 assumed | 0.04 | 0.2 |
| C030 | Lambert et al. (2008) | Body mass loss (%) | 20 | Reconstructed from marginal dispersions | 0.50 assumed | 0.008105 | 0.0900278 |
| C031 | McConell et al. (1997) | Body mass loss (%) | 7 | Reconstructed from marginal dispersions | 0.50 assumed | 0.01 | 0.1 |
| C032 | Montain et al. (1992a) | Body mass loss (%) | 7 | Reconstructed from marginal dispersions | 0.50 assumed | 0.04 | 0.2 |
| C033 | Nassis et al. (2002) | Body mass loss (%) | 11 | Reconstructed from marginal dispersions | 0.50 assumed | 0.02853565 | 0.168925 |
| C034 | Perreault-Brière et al. (2019) | Body mass loss (%) | 9 | Reconstructed from marginal dispersions | 0.50 assumed | 0.01333333 | 0.11547 |
| C036 | Costa et al. (2019) | Plasma volume change (%) | 11 | Reconstructed from marginal dispersions | 0.50 assumed | 2.368882 | 1.53912 |
| C037 | Fallowfield et al. (1996) | Plasma volume change (%) | 8 | Reconstructed from marginal dispersions | 0.50 assumed | 1.21 | 1.1 |
| C038 | Maughan et al. (1996) | Plasma volume change (%) | 12 | Reconstructed from marginal dispersions | 0.50 assumed | 1.57 | 1.253 |
| C039 | McConell et al. (1997) | Plasma volume change (%) | 7 | Reconstructed from marginal dispersions | 0.50 assumed | 2.73 | 1.65227 |
| C041 | Perreault-Brière et al. (2019) | Plasma volume change (%) | 9 | Reconstructed from marginal dispersions | 0.50 assumed | 5.92 | 2.43311 |
| C042 | Sanders et al. (1999) | Plasma volume change (%) | 6 | Reconstructed from marginal dispersions | 0.50 assumed | 1.83 | 1.35277 |
| C044 | Costa et al. (2019) | Rating of perceived exertion (Borg 6-20) | 11 | Reconstructed from marginal dispersions | 0.50 assumed | 0.8446182 | 0.919031 |
| C045 | Lambert et al. (2008) | Rating of perceived exertion (Borg 6-20) | 20 | Reconstructed from marginal dispersions | 0.50 assumed | 0.1505 | 0.387943 |
| C046 | Robinson et al. (1995) | Rating of perceived exertion (Borg 6-20) | 8 | Reconstructed from marginal dispersions | 0.50 assumed | 0.12 | 0.34641 |
| C047 | Perreault-Brière et al. (2019) | Abdominal discomfort | 9 | Reconstructed from marginal dispersions | 0.50 assumed | 0.06777778 | 0.260342 |
| C048 | Fallowfield et al. (1996) | Plasma sodium concentration | 8 | Reconstructed from marginal dispersions | 0.50 assumed | 1.29 | 1.13578 |
| C049 | Robinson et al. (1995) | Plasma sodium concentration | 8 | Reconstructed from marginal dispersions | 0.50 assumed | 0.31 | 0.556776 |
| C050 | Sanders et al. (1999) | Plasma sodium concentration | 6 | Reconstructed from marginal dispersions | 0.50 assumed | 1 | 1 |
| C052 | Perreault-Brière et al. (2019) | Plasma sodium change | 9 | Reconstructed from marginal dispersions | 0.50 assumed | 13.98111 | 3.73913 |
| C053 | Costa et al. (2019) | Serum/plasma osmolality | 11 | Reconstructed from marginal dispersions | 0.50 assumed | 17.44545 | 4.17677 |
| C054 | Robinson et al. (1995) | Serum/plasma osmolality | 8 | Reconstructed from marginal dispersions | 0.50 assumed | 3.27 | 1.80831 |
| C055 | Dugas et al. (2009) | Urine volume (L) | 6 | Reconstructed from marginal dispersions | 0.50 assumed | 0.002579167 | 0.0507855 |
| C056 | McConell et al. (1997) | Urine volume (L) | 7 | Reconstructed from marginal dispersions | 0.50 assumed | 0.018228 | 0.135011 |
| C057 | Perreault-Brière et al. (2019) | Urine volume (L) | 9 | Reconstructed from marginal dispersions | 0.50 assumed | 0.001908333 | 0.0436845 |
| C058 | Robinson et al. (1995) | Urine volume (L) | 8 | Reconstructed from marginal dispersions | 0.50 assumed | 0.0019 | 0.043589 |
| C060 | Dugas et al. (2009) | Whole-body sweat rate | 6 | Reconstructed from marginal dispersions | 0.50 assumed | 0.001716667 | 0.0414327 |
| C062 | Kay et al. (2003) | Whole-body sweat rate | 7 | Reconstructed from marginal dispersions | 0.50 assumed | 0.02000991 | 0.141456 |
| C063 | Lambert et al. (2008) | Whole-body sweat rate | 20 | Reconstructed from marginal dispersions | 0.50 assumed | 0.0043368 | 0.0658544 |
| C064 | Montain et al. (1992a) | Whole-body sweat rate | 7 | Reconstructed from marginal dispersions | 0.50 assumed | 0.0049 | 0.07 |
| C065 | Nassis et al. (2002) | Whole-body sweat rate | 11 | Reconstructed from marginal dispersions | 0.50 assumed | 0.004091874 | 0.0639678 |
| C066 | Robinson et al. (1995) | Whole-body sweat rate | 8 | Reconstructed from marginal dispersions | 0.50 assumed | 0.0175 | 0.132288 |
| C067 | Juett et al. (2024) | Fluid intake (L) | 12 | Reconstructed from marginal dispersions | 0.50 assumed | 0.06243358 | 0.249867 |
| C068 | Kay et al. (2003) | Fluid intake (L) | 7 | Reconstructed from marginal dispersions | 0.50 assumed | 0.01714286 | 0.130931 |
| C069 | McConell et al. (1997) | Fluid intake (L) | 7 | Reconstructed from marginal dispersions | 0.50 assumed | 0.01 | 0.1 |
| C070 | Montain et al. (1992a) | Fluid intake (L) | 7 | Reconstructed from marginal dispersions | 0.50 assumed | 0.010609 | 0.103 |
| C071 | Nassis et al. (2002) | Fluid intake (L) | 11 | Reconstructed from marginal dispersions | 0.50 assumed | 0.00515725 | 0.071814 |
| C072 | Perreault-Brière et al. (2019) | Fluid intake (L) | 9 | Reconstructed from marginal dispersions | 0.50 assumed | 0.001736111 | 0.0416667 |
| C073 | Robinson et al. (1995) | Fluid intake (L) | 8 | Reconstructed from marginal dispersions | 0.50 assumed | 0.0196 | 0.14 |
| C074 | Dugas et al. (2009) | Fluid intake rate (L/h) | 6 | Reconstructed from marginal dispersions | 0.50 assumed | 0.0054 | 0.0734847 |
| C075 | Costa et al. (2019) | Body mass change (kg) | 11 | Reconstructed from marginal dispersions | 0.50 assumed | 2.199527 | 1.48308 |
| C076 | Kay et al. (2003) | Body mass change (kg) | 7 | Reconstructed from marginal dispersions | 0.50 assumed | 0.01704151 | 0.130543 |
| C077 | McConell et al. (1997) | Body mass change (kg) | 7 | Reconstructed from marginal dispersions | 0.50 assumed | 0.0093 | 0.0964365 |
| C079 | Nassis et al. (2002) | Body mass change (kg) | 11 | Reconstructed from marginal dispersions | 0.50 assumed | 0.0099758 | 0.0998789 |
| C080 | Walsh et al. (1994) | Body mass change (kg) | 6 | Reconstructed from marginal dispersions | 0.50 assumed | 0.01911667 | 0.138263 |
| C081 | Robinson et al. (1995) | Body mass change rate | 8 | Reconstructed from marginal dispersions | 0.50 assumed | 0.0189 | 0.137477 |
| C082 | Costa et al. (2019) | Breath hydrogen AUC | 11 | Reconstructed from marginal dispersions | 0.50 assumed | 79031.99 | 281.126 |
| C083 | Nassis et al. (2002) | Cardiac output change | 11 | Reconstructed from marginal dispersions | 0.50 assumed | 0.21 | 0.458258 |
| C084 | Sanders et al. (1999) | Fluid balance | 6 | Reconstructed from marginal dispersions | 0.50 assumed | 0.01 | 0.1 |
| C085 | Robinson et al. (1995) | Fluid retained | 8 | Reconstructed from marginal dispersions | 0.50 assumed | 0.0144 | 0.12 |
| C087 | Robinson et al. (1995) | Gastric residual volume | 8 | Reconstructed from marginal dispersions | 0.50 assumed | 0.0064 | 0.08 |
| C089 | Nassis et al. (2002) | Local sweat rate | 11 | Reconstructed from marginal dispersions | 0.50 assumed | 0.001476925 | 0.0384308 |
| C090 | Nassis et al. (2002) | Mean skin temperature | 11 | Reconstructed from marginal dispersions | 0.50 assumed | 0.3789275 | 0.615571 |
| C091 | Robinson et al. (1995) | Mean skin temperature | 8 | Reconstructed from marginal dispersions | 0.50 assumed | 0.36 | 0.6 |
| C092 | Walsh et al. (1994) | Mean skin temperature | 6 | Reconstructed from marginal dispersions | 0.50 assumed | 0.06 | 0.244949 |
| C093 | Walsh et al. (1994) | Rating of perceived exertion (CR10) | 6 | Reconstructed from marginal dispersions | 0.50 assumed | 0.08666667 | 0.294392 |
| C094 | Nassis et al. (2002) | Skin blood flow | 11 | Reconstructed from marginal dispersions | 0.50 assumed | 71.41 | 8.45044 |
| C095 | Sanders et al. (1999) | Sodium balance | 6 | Reconstructed from marginal dispersions | 0.50 assumed | 61 | 7.81025 |
| C096 | Nassis et al. (2002) | Stroke volume change | 11 | Reconstructed from marginal dispersions | 0.50 assumed | 11.83 | 3.43948 |
| C097 | Dugas et al. (2009) | Sweat loss | 6 | Reconstructed from marginal dispersions | 0.50 assumed | 0.005216667 | 0.0722265 |
| C098 | McConell et al. (1997) | Sweat loss | 7 | Reconstructed from marginal dispersions | 0.50 assumed | 0.01 | 0.1 |
| C099 | Perreault-Brière et al. (2019) | Sweat loss | 9 | Reconstructed from marginal dispersions | 0.50 assumed | 0.003488111 | 0.0590602 |
| C101 | Dugas et al. (2009) | Sweat loss replaced | 6 | Reconstructed from marginal dispersions | 0.50 assumed | 19.44 | 4.40908 |
| C102 | McConell et al. (1997) | Sweat loss replaced | 7 | Reconstructed from marginal dispersions | 0.50 assumed | 21.16 | 4.6 |
| C103 | Montain et al. (1992a) | Sweat loss replaced | 7 | Reconstructed from marginal dispersions | 0.50 assumed | 4.84 | 2.2 |
| C104 | Perreault-Brière et al. (2019) | Thirst sensation | 9 | Reconstructed from marginal dispersions | 0.50 assumed | 0.4311111 | 0.656591 |
| C105 | Dion et al. (2013) | Time-trial/fixed-duration performance | 10 | Reconstructed from marginal dispersions | 0.50 assumed | 0.0007368842 | 0.0271456 |
| C106 | Jeker et al. (2022) | Time-trial/fixed-duration performance | 8 | Reconstructed from marginal dispersions | 0.50 assumed | 0.0003937908 | 0.0198442 |
| C108 | Perreault-Brière et al. (2019) | Time-trial/fixed-duration performance | 9 | Reconstructed from marginal dispersions | 0.50 assumed | 0.000347752 | 0.0186481 |
| C109 | Dion et al. (2013) | Core temperature | 10 | Reconstructed from marginal dispersions | 0.50 assumed | 0.013 | 0.114018 |
| C110 | Jeker et al. (2022) | Core temperature | 8 | Reconstructed from marginal dispersions | 0.50 assumed | 0.02 | 0.141421 |
| C111 | Lopez et al. (2016) | Core temperature | 13 | Reconstructed from marginal dispersions | 0.50 assumed | 0.03762308 | 0.193967 |
| C112 | Dion et al. (2013) | Heart rate | 10 | Reconstructed from marginal dispersions | 0.50 assumed | 7.3 | 2.70185 |
| C113 | Perreault-Brière et al. (2019) | Heart rate | 9 | Reconstructed from marginal dispersions | 0.50 assumed | 2.333333 | 1.52753 |
| C114 | Dion et al. (2013) | Body mass loss (%) | 10 | Reconstructed from marginal dispersions | 0.50 assumed | 0.043 | 0.207364 |
| C115 | Jeker et al. (2022) | Body mass loss (%) | 8 | Reconstructed from marginal dispersions | 0.50 assumed | 0.11375 | 0.337268 |
| C116 | Lopez et al. (2016) | Body mass loss (%) | 13 | Reconstructed from marginal dispersions | 0.50 assumed | 0.02120769 | 0.145629 |
| C117 | Perreault-Brière et al. (2019) | Body mass loss (%) | 9 | Reconstructed from marginal dispersions | 0.50 assumed | 0.007777778 | 0.0881917 |
| C118 | Perreault-Brière et al. (2019) | Plasma volume change (%) | 9 | Reconstructed from marginal dispersions | 0.50 assumed | 7.241111 | 2.69093 |
| C119 | Jeker et al. (2022) | Abdominal discomfort | 8 | Reconstructed from marginal dispersions | 0.50 assumed | 0.02375 | 0.15411 |
| C120 | Perreault-Brière et al. (2019) | Abdominal discomfort | 9 | Reconstructed from marginal dispersions | 0.50 assumed | 0.06777778 | 0.260342 |
| C121 | Perreault-Brière et al. (2019) | Plasma sodium change | 9 | Reconstructed from marginal dispersions | 0.50 assumed | 16.12 | 4.01497 |
| C122 | Jeker et al. (2022) | Urine volume (L) | 8 | Reconstructed from marginal dispersions | 0.50 assumed | 0.1009916 | 0.317792 |
| C123 | Perreault-Brière et al. (2019) | Urine volume (L) | 9 | Reconstructed from marginal dispersions | 0.50 assumed | 0.001855444 | 0.0430749 |
| C124 | Dion et al. (2013) | Whole-body sweat rate | 10 | Reconstructed from marginal dispersions | 0.50 assumed | 0.00903 | 0.0950263 |
| C125 | Jeker et al. (2022) | Whole-body sweat rate | 8 | Reconstructed from marginal dispersions | 0.50 assumed | 0.001431125 | 0.0378302 |
| C126 | Lopez et al. (2016) | Whole-body sweat rate | 13 | Reconstructed from marginal dispersions | 0.50 assumed | 0.01039231 | 0.101943 |
| C127 | Dion et al. (2013) | Fluid intake (L) | 10 | Reconstructed from marginal dispersions | 0.50 assumed | 0.01687 | 0.129885 |
| C128 | Jeker et al. (2022) | Fluid intake (L) | 8 | Reconstructed from marginal dispersions | 0.50 assumed | 0.1349126 | 0.367305 |
| C129 | Lopez et al. (2016) | Fluid intake (L) | 13 | Reconstructed from marginal dispersions | 0.50 assumed | 0.02361792 | 0.153681 |
| C130 | Perreault-Brière et al. (2019) | Fluid intake (L) | 9 | Reconstructed from marginal dispersions | 0.50 assumed | 0.002784333 | 0.0527668 |
| C131 | Dion et al. (2013) | Fluid intake rate (L/h) | 10 | Reconstructed from marginal dispersions | 0.50 assumed | 0.00772 | 0.0878635 |
| C132 | Jeker et al. (2022) | Fluid balance | 8 | Reconstructed from marginal dispersions | 0.50 assumed | 0.07262362 | 0.269488 |
| C133 | Dion et al. (2013) | Mean body temperature | 10 | Reconstructed from marginal dispersions | 0.50 assumed | 0.012 | 0.109545 |
| C134 | Jeker et al. (2022) | Sodium intake rate | 8 | Reconstructed from marginal dispersions | 0.50 assumed | 186.125 | 13.6428 |
| C135 | Dion et al. (2013) | Sweat loss | 10 | Reconstructed from marginal dispersions | 0.50 assumed | 0.00999 | 0.09995 |
| C136 | Jeker et al. (2022) | Sweat loss | 8 | Reconstructed from marginal dispersions | 0.50 assumed | 0.04636162 | 0.215317 |
| C137 | Perreault-Brière et al. (2019) | Sweat loss | 9 | Reconstructed from marginal dispersions | 0.50 assumed | 0.003164778 | 0.0562564 |
| C138 | Lopez et al. (2016) | Sweat loss replaced | 13 | Reconstructed from marginal dispersions | 0.50 assumed | 17.53846 | 4.18789 |
| C139 | Perreault-Brière et al. (2019) | Thirst sensation | 9 | Reconstructed from marginal dispersions | 0.50 assumed | 0.2533333 | 0.503322 |
| C140 | Lopez et al. (2016) | Urine osmolality | 13 | Reconstructed from marginal dispersions | 0.50 assumed | 3199 | 56.5597 |
| C141 | Dugas et al. (2009) | Time-trial/fixed-duration performance | 6 | Reconstructed from marginal dispersions | 0.50 assumed | 0.0003046016 | 0.0174528 |
| C142 | McConell et al. (1997) | Time-to-exhaustion performance | 7 | Reconstructed from marginal dispersions | 0.50 assumed | 0.1442112 | 0.379751 |
| C143 | Sage et al. (2026) | Time-trial/fixed-duration performance | 11 | Author-reported paired precision | 0.513 | 0.001145962 | 0.0338521 |
| C144 | Sage et al. (2026) | Mean power output (alternative measure) | 11 | Author-reported paired precision | Not required / directly reported | 61.68671 | 7.85409 |
| C145 | Dugas et al. (2009) | Core temperature | 6 | Reconstructed from marginal dispersions | 0.50 assumed | 0.035 | 0.187083 |
| C146 | Sage et al. (2026) | Core temperature | 11 | Author-reported paired precision | Not required / directly reported | 0.005821211 | 0.0762969 |
| C147 | McConell et al. (1997) | Body mass loss (%) | 7 | Reconstructed from marginal dispersions | 0.50 assumed | 0.01 | 0.1 |
| C148 | Sage et al. (2026) | Body mass loss (%) | 11 | Author-reported paired precision | Not required / directly reported | 0.01812834 | 0.134642 |
| C149 | Sage et al. (2026) | Body mass loss (%) | 11 | Reconstructed from marginal dispersions | 0.50 assumed | 0.06636364 | 0.257611 |
| C150 | McConell et al. (1997) | Plasma volume change (%) | 7 | Reconstructed from marginal dispersions | 0.50 assumed | 2.17 | 1.47309 |
| C151 | Sage et al. (2026) | Plasma volume change (%) | 9 | Author-reported paired precision | Not required / directly reported | 1.692475 | 1.30095 |
| C152 | Dugas et al. (2009) | Urine volume (L) | 6 | Reconstructed from marginal dispersions | 0.50 assumed | 0.002300167 | 0.0479601 |
| C153 | McConell et al. (1997) | Urine volume (L) | 7 | Reconstructed from marginal dispersions | 0.50 assumed | 0.017353 | 0.131731 |
| C154 | Sage et al. (2026) | Urine volume (L) | 11 | Reconstructed from marginal dispersions | 0.50 assumed | 0.007960727 | 0.0892229 |
| C155 | Dugas et al. (2009) | Whole-body sweat rate | 6 | Reconstructed from marginal dispersions | 0.50 assumed | 0.00195 | 0.0441588 |
| C156 | McConell et al. (1997) | Fluid intake (L) | 7 | Reconstructed from marginal dispersions | 0.50 assumed | 0.0075 | 0.0866025 |
| C157 | Sage et al. (2026) | Fluid intake (L) | 11 | Reconstructed from marginal dispersions | 0.50 assumed | 0.02147264 | 0.146535 |
| C158 | Dugas et al. (2009) | Fluid intake rate (L/h) | 6 | Reconstructed from marginal dispersions | 0.50 assumed | 0.004962667 | 0.0704462 |
| C159 | Sage et al. (2026) | Fluid intake rate (L/h) | 11 | Reconstructed from marginal dispersions | 0.50 assumed | 0.005347909 | 0.0731294 |
| C160 | McConell et al. (1997) | Body mass change (kg) | 7 | Reconstructed from marginal dispersions | 0.50 assumed | 0.0057 | 0.0754983 |
| C161 | Dugas et al. (2009) | Sweat loss | 6 | Reconstructed from marginal dispersions | 0.50 assumed | 0.00755 | 0.0868907 |
| C162 | McConell et al. (1997) | Sweat loss | 7 | Reconstructed from marginal dispersions | 0.50 assumed | 0.01 | 0.1 |
| C163 | Dugas et al. (2009) | Sweat loss replaced | 6 | Reconstructed from marginal dispersions | 0.50 assumed | 14.74667 | 3.84014 |
| C164 | McConell et al. (1997) | Sweat loss replaced | 7 | Reconstructed from marginal dispersions | 0.50 assumed | 15.96 | 3.995 |
| C165 | Sage et al. (2026) | Total body water | 11 | Author-reported paired precision | Not required / directly reported | 0.02467468 | 0.157082 |
| C166 | Sage et al. (2026) | Urine sodium loss | 11 | Reconstructed from marginal dispersions | 0.50 assumed | 8.528518 | 2.92036 |
| C167 | Wijering et al. (2023) | Capped exercise duration | 11 | Reconstructed from marginal dispersions | 0.50 assumed | 6.090909 | 2.46798 |
| C168 | Sanders et al. (1999) | Heart rate | 6 | Reconstructed from marginal dispersions | 0.50 assumed | 3 | 1.73205 |
| C169 | Wijering et al. (2023) | Body mass loss (%) | 11 | Reconstructed from marginal dispersions | 0.50 assumed | 0.01659091 | 0.128806 |
| C170 | Sanders et al. (1999) | Plasma volume change (%) | 6 | Reconstructed from marginal dispersions | 0.50 assumed | 1.83 | 1.35277 |
| C171 | Wijering et al. (2023) | Plasma volume change (%) | 11 | Author-reported paired precision | Not required / directly reported | 0.3670989 | 0.605887 |
| C172 | Sanders et al. (1999) | Plasma sodium concentration | 6 | Reconstructed from marginal dispersions | 0.50 assumed | 1 | 1 |
| C173 | Wijering et al. (2023) | Plasma sodium change | 11 | Author-reported paired precision | Not required / directly reported | 1.019719 | 1.00981 |
| C174 | Wijering et al. (2023) | Whole-body sweat rate | 7 | Reconstructed from marginal dispersions | 0.50 assumed | 0.005842857 | 0.0764386 |
| C175 | Wijering et al. (2023) | Fluid intake (L) | 11 | Reconstructed from marginal dispersions | 0.50 assumed | 0.03587891 | 0.189417 |
| C176 | Wijering et al. (2023) | Fluid intake rate (L/h) | 11 | Reconstructed from marginal dispersions | 0.50 assumed | 0.003663636 | 0.060528 |
| C177 | Wijering et al. (2023) | Body mass change (kg) | 11 | Reconstructed from marginal dispersions | 0.50 assumed | 0.009772727 | 0.0988571 |
| C178 | Sanders et al. (1999) | Fluid balance | 6 | Reconstructed from marginal dispersions | 0.50 assumed | 0.01 | 0.1 |
| C179 | Sanders et al. (1999) | Sodium balance | 6 | Reconstructed from marginal dispersions | 0.50 assumed | 81 | 9 |
| C180 | Wijering et al. (2023) | Sodium intake | 11 | Reconstructed from marginal dispersions | 0.50 assumed | 114.9091 | 10.7196 |
| C181 | Wijering et al. (2023) | Sweat loss | 7 | Reconstructed from marginal dispersions | 0.50 assumed | 0.0553 | 0.23516 |
| C182 | Montain et al. (1993) | Core temperature | 7 | Reconstructed from marginal dispersions | 0.50 assumed | 0.0103 | 0.101489 |
| C183 | Montain et al. (1993) | Heart rate | 7 | Reconstructed from marginal dispersions | 0.50 assumed | 9 | 3 |
| C184 | Montain et al. (1993) | Fluid intake (L) | 7 | Reconstructed from marginal dispersions | 0.50 assumed | 0.001936 | 0.044 |
| C186 | Gonzalez-Alonso et al. (1995) | Body mass loss (%) | 7 | Reconstructed from marginal dispersions | 0.50 assumed | 0.0271 | 0.164621 |
| C187 | Gonzalez-Alonso et al. (1995) | Core temperature | 7 | Reconstructed from marginal dispersions | 0.50 assumed | 0.03 | 0.173205 |
| C188 | Gonzalez-Alonso et al. (1995) | Fluid intake (L) | 7 | Reconstructed from marginal dispersions | 0.50 assumed | 0.0183 | 0.135277 |
| C189 | Gonzalez-Alonso et al. (1995) | Heart rate | 7 | Reconstructed from marginal dispersions | 0.50 assumed | 7 | 2.64575 |
| C190 | Gonzalez-Alonso et al. (1995) | Plasma sodium concentration | 7 | Reconstructed from marginal dispersions | 0.50 assumed | 1 | 1 |
| C191 | Gonzalez-Alonso et al. (1995) | Plasma volume change (%) | 7 | Reconstructed from marginal dispersions | 0.50 assumed | 1.51 | 1.22882 |
| C192 | Gonzalez-Alonso et al. (1995) | Serum/plasma osmolality | 7 | Reconstructed from marginal dispersions | 0.50 assumed | 7 | 2.64575 |
| C193 | Gonzalez-Alonso et al. (1995) | Sweat loss replaced | 7 | Reconstructed from marginal dispersions | 0.50 assumed | 7.21 | 2.68514 |
| C194 | Gonzalez-Alonso et al. (1995) | Whole-body sweat rate | 7 | Reconstructed from marginal dispersions | 0.50 assumed | 0.0121 | 0.11 |
| C195 | Backx et al. (2003) | Abdominal discomfort | 8 | Reconstructed from marginal dispersions | 0.50 assumed | 0.11375 | 0.337268 |
| C196 | Backx et al. (2003) | Body mass loss (%) | 8 | Reconstructed from marginal dispersions | 0.50 assumed | 0.0278915 | 0.167007 |
| C197 | Backx et al. (2003) | Time-trial/fixed-duration performance | 8 | Reconstructed from marginal dispersions | 0.50 assumed | 0.0003278577 | 0.0181068 |
| C198 | Backx et al. (2003) | Fluid intake (L) | 8 | Reconstructed from marginal dispersions | 0.50 assumed | 0.000351125 | 0.0187383 |
| C199 | Backx et al. (2003) | Heart rate | 8 | Reconstructed from marginal dispersions | 0.50 assumed | 9 | 3 |
| C200 | Backx et al. (2003) | Sweat loss replaced | 8 | Reconstructed from marginal dispersions | 0.50 assumed | 13.875 | 3.72492 |
| C201 | Claveau et al. (2022) | Fluid intake (L) | 10 | Reconstructed from marginal dispersions | 0.50 assumed | 0.0716119 | 0.267604 |
| C202 | Claveau et al. (2022) | Sweat loss replaced | 10 | Reconstructed from marginal dispersions | 0.50 assumed | 20.1 | 4.4833 |
| C203 | Claveau et al. (2022) | Body mass loss (%) | 10 | Reconstructed from marginal dispersions | 0.50 assumed | 0.112 | 0.334664 |
| C204 | Claveau et al. (2022) | Urine volume (L) | 10 | Reconstructed from marginal dispersions | 0.50 assumed | 0.0016527 | 0.0406534 |
| C205 | Claveau et al. (2022) | Core temperature | 10 | Reconstructed from marginal dispersions | 0.50 assumed | 0.043 | 0.207364 |
| C206 | Claveau et al. (2022) | Heart rate | 9 | Reconstructed from marginal dispersions | 0.50 assumed | 44.77778 | 6.69162 |
| C207 | Claveau et al. (2022) | Rating of perceived exertion (Borg 6-20) | 10 | Reconstructed from marginal dispersions | 0.50 assumed | 0.7 | 0.83666 |
| C208 | Claveau et al. (2022) | Urine osmolality | 9 | Reconstructed from marginal dispersions | 0.50 assumed | 6124.333 | 78.2581 |
| C209 | Claveau et al. (2022) | Urine specific gravity | 10 | Reconstructed from marginal dispersions | 0.50 assumed | 4.9e-06 | 0.00221359 |

Fallowfield TTE and Sage TT recover r≈0.940 and r≈0.513, respectively. Wijering PV/sodium change and Sage’s additional paired contrasts are also author-reported paired evidence. The denominator n is outcome-specific; Sage PV uses nine blood samples. CI-width recovery assumes t(n−1) and is an approximation when the source uses another model.

**Table S7. GRADE judgments for every estimable critical or important comparison–outcome pair.**

Forty assessments cover 22 pooled and 18 single-study pairs. Supporting fluid-intake/manipulation outcomes are not graded as efficacy endpoints. Rules and thresholds are stated in the Methods; all rules are post hoc and the 300/200 information-size benchmarks are review-defined decision aids, not calculated optimal information sizes. No certainty category is chosen according to whether p<.05.

| **Comparison** | **Outcome** | **Studies (N)** | **Effect (95% CI)** | **Domain judgments and rationale** | **Certainty** |
| --- | --- | --- | --- | --- | --- |
| Oral hydration vs no or minimal fluid | Abdominal discomfort | 1 (9) | 0.40 (-0.11 to 0.91) AU | Risk of bias −2: 100.0% high-risk and 0.0% some-concerns random-effects weight. Inconsistency −0: Single study; between-study inconsistency cannot be evaluated. Imprecision −2: N/benchmark=0.04; boundary ±1.0 AU. Indirectness −0: direct eligible contrast. Publication bias −0: no additional directional evidence; absence not established. | Very low |
| Oral hydration vs no or minimal fluid | Body mass loss (%) | 7 (72) | -2.63 (-3.58 to -1.69) percentage points | Risk of bias −1: 0.0% high-risk and 100.0% some-concerns random-effects weight. Inconsistency −1: I²=98.3%; prediction interval -5.41 to 0.14; opposite-signed study estimates: no. Imprecision −1: N/benchmark=0.36; boundary ±1.0 percentage points. Indirectness −0: direct eligible contrast. Publication bias −0: no additional directional evidence; absence not established. | Very low |
| Oral hydration vs no or minimal fluid | Core temperature | 9 (80) | -0.38 (-0.74 to -0.02) °C | Risk of bias −1: 0.0% high-risk and 100.0% some-concerns random-effects weight. Inconsistency −2: I²=87.3%; prediction interval -1.46 to 0.69; opposite-signed study estimates: yes. Imprecision −1: N/benchmark=0.40; boundary ±0.3 °C. Indirectness −0: direct eligible contrast. Publication bias −0: no additional directional evidence; absence not established. | Very low |
| Oral hydration vs no or minimal fluid | Heart rate | 12 (115) | -6.82 (-11.16 to -2.48) beats/min | Risk of bias −1: 0.0% high-risk and 100.0% some-concerns random-effects weight. Inconsistency −2: I²=83.3%; prediction interval -20.38 to 6.73; opposite-signed study estimates: yes. Imprecision −1: N/benchmark=0.57; boundary ±5.0 beats/min. Indirectness −0: direct eligible contrast. Publication bias −0: no additional directional evidence; absence not established. | Very low |
| Oral hydration vs no or minimal fluid | Plasma sodium change | 1 (9) | 0.20 (-7.13 to 7.53) mmol/L | Risk of bias −1: 0.0% high-risk and 100.0% some-concerns random-effects weight. Inconsistency −0: Single study; between-study inconsistency cannot be evaluated. Imprecision −2: N/benchmark=0.04; boundary ±2.0 mmol/L. Indirectness −0: direct eligible contrast. Publication bias −0: no additional directional evidence; absence not established. | Very low |
| Oral hydration vs no or minimal fluid | Plasma sodium concentration | 4 (29) | -4.37 (-10.18 to 1.43) mmol/L | Risk of bias −1: 0.0% high-risk and 100.0% some-concerns random-effects weight. Inconsistency −1: I²=91.7%; prediction interval -21.32 to 12.57; opposite-signed study estimates: no. Imprecision −2: N/benchmark=0.14; boundary ±2.0 mmol/L. Indirectness −0: direct eligible contrast. Publication bias −0: no additional directional evidence; absence not established. | Very low |
| Oral hydration vs no or minimal fluid | Plasma volume change (%) | 7 (60) | 3.28 (0.42 to 6.14) percentage points | Risk of bias −1: 0.0% high-risk and 100.0% some-concerns random-effects weight. Inconsistency −2: I²=82.7%; prediction interval -4.29 to 10.84; opposite-signed study estimates: yes. Imprecision −1: N/benchmark=0.30; boundary ±3.0 percentage points. Indirectness −0: direct eligible contrast. Publication bias −0: no additional directional evidence; absence not established. | Very low |
| Oral hydration vs no or minimal fluid | Rating of perceived exertion (Borg 6-20) | 3 (39) | -0.54 (-2.11 to 1.03) AU | Risk of bias −2: 100.0% high-risk and 0.0% some-concerns random-effects weight. Inconsistency −0: I²=38.8%; prediction interval -7.55 to 6.47; opposite-signed study estimates: no. Imprecision −2: N/benchmark=0.20; boundary ±1.0 AU. Indirectness −0: direct eligible contrast. Publication bias −0: no additional directional evidence; absence not established. | Very low |
| Oral hydration vs no or minimal fluid | Serum/plasma osmolality | 3 (26) | -12.75 (-34.19 to 8.69) mOsm/kg | Risk of bias −1: 0.0% high-risk and 100.0% some-concerns random-effects weight. Inconsistency −1: I²=92.5%; prediction interval -133.64 to 108.14; opposite-signed study estimates: no. Imprecision −2: N/benchmark=0.13; boundary ±5.0 mOsm/kg. Indirectness −0: direct eligible contrast. Publication bias −0: no additional directional evidence; absence not established. | Very low |
| Oral hydration vs no or minimal fluid | Time-to-exhaustion performance | 3 (21) | 34.1% (11.9% to 60.7%) | Risk of bias −1: 0.0% high-risk and 100.0% some-concerns random-effects weight. Inconsistency −0: I²=0.0%; prediction interval -21.4% to 128.8%; opposite-signed study estimates: no. Imprecision −2: N/benchmark=0.07; boundary ±2%. Indirectness −0: direct eligible contrast. Publication bias −0: no additional directional evidence; absence not established. | Very low |
| Oral hydration vs no or minimal fluid | Time-trial/fixed-duration performance | 4 (30) | -0.1% (-3.6% to 3.5%) | Risk of bias −1: 0.0% high-risk and 100.0% some-concerns random-effects weight. Inconsistency −0: I²=1.8%; prediction interval -6.2% to 6.4%; opposite-signed study estimates: yes. Imprecision −2: N/benchmark=0.10; boundary ±2%. Indirectness −0: direct eligible contrast. Publication bias −0: no additional directional evidence; absence not established. | Very low |
| Oral hydration vs no or minimal fluid | Urine volume (L) | 4 (30) | 0.06 (-0.05 to 0.16) L | Risk of bias −1: 0.0% high-risk and 100.0% some-concerns random-effects weight. Inconsistency −0: I²=28.3%; prediction interval -0.15 to 0.26; opposite-signed study estimates: yes. Imprecision −2: N/benchmark=0.15; boundary ±0.2 L. Indirectness −0: direct eligible contrast. Publication bias −0: no additional directional evidence; absence not established. | Very low |
| Programmed or individualized vs self-regulated drinking | Abdominal discomfort | 2 (17) | 0.23 (-1.46 to 1.91) AU | Risk of bias −2: 100.0% high-risk and 0.0% some-concerns random-effects weight. Inconsistency −0: I²=0.0%; prediction interval Not estimable (k<3); opposite-signed study estimates: no. Imprecision −2: N/benchmark=0.09; boundary ±1.0 AU. Indirectness −0: direct eligible contrast. Publication bias −0: no additional directional evidence; absence not established. | Very low |
| Programmed or individualized vs self-regulated drinking | Body mass loss (%) | 4 (40) | -1.47 (-1.96 to -0.99) percentage points | Risk of bias −1: 0.0% high-risk and 100.0% some-concerns random-effects weight. Inconsistency −1: I²=62.4%; prediction interval -2.61 to -0.34; opposite-signed study estimates: no. Imprecision −2: N/benchmark=0.20; boundary ±1.0 percentage points. Indirectness −0: direct eligible contrast. Publication bias −0: no additional directional evidence; absence not established. | Very low |
| Programmed or individualized vs self-regulated drinking | Core temperature | 3 (31) | -0.23 (-0.58 to 0.12) °C | Risk of bias −1: 0.0% high-risk and 100.0% some-concerns random-effects weight. Inconsistency −0: I²=0.0%; prediction interval -1.26 to 0.79; opposite-signed study estimates: no. Imprecision −2: N/benchmark=0.15; boundary ±0.3 °C. Indirectness −0: direct eligible contrast. Publication bias −0: no additional directional evidence; absence not established. | Very low |
| Programmed or individualized vs self-regulated drinking | Heart rate | 2 (19) | -3.24 (-20.14 to 13.65) beats/min | Risk of bias −1: 0.0% high-risk and 100.0% some-concerns random-effects weight. Inconsistency −0: I²=0.0%; prediction interval Not estimable (k<3); opposite-signed study estimates: no. Imprecision −2: N/benchmark=0.10; boundary ±5.0 beats/min. Indirectness −0: direct eligible contrast. Publication bias −0: no additional directional evidence; absence not established. | Very low |
| Programmed or individualized vs self-regulated drinking | Plasma sodium change | 1 (9) | -3.90 (-11.77 to 3.97) mmol/L | Risk of bias −1: 0.0% high-risk and 100.0% some-concerns random-effects weight. Inconsistency −0: Single study; between-study inconsistency cannot be evaluated. Imprecision −2: N/benchmark=0.04; boundary ±2.0 mmol/L. Indirectness −0: direct eligible contrast. Publication bias −0: no additional directional evidence; absence not established. | Very low |
| Programmed or individualized vs self-regulated drinking | Plasma volume change (%) | 1 (9) | 2.70 (-2.57 to 7.97) percentage points | Risk of bias −1: 0.0% high-risk and 100.0% some-concerns random-effects weight. Inconsistency −0: Single study; between-study inconsistency cannot be evaluated. Imprecision −2: N/benchmark=0.04; boundary ±3.0 percentage points. Indirectness −0: direct eligible contrast. Publication bias −0: no additional directional evidence; absence not established. | Very low |
| Programmed or individualized vs self-regulated drinking | Time-trial/fixed-duration performance | 3 (27) | 0.7% (-4.4% to 6.2%) | Risk of bias −1: 0.0% high-risk and 100.0% some-concerns random-effects weight. Inconsistency −0: I²=0.0%; prediction interval -13.7% to 17.6%; opposite-signed study estimates: yes. Imprecision −2: N/benchmark=0.09; boundary ±2%. Indirectness −0: direct eligible contrast. Publication bias −0: no additional directional evidence; absence not established. | Very low |
| Programmed or individualized vs self-regulated drinking | Urine volume (L) | 2 (17) | 0.39 (-4.81 to 5.59) L | Risk of bias −1: 0.0% high-risk and 100.0% some-concerns random-effects weight. Inconsistency −1: I²=85.0%; prediction interval Not estimable (k<3); opposite-signed study estimates: no. Imprecision −2: N/benchmark=0.09; boundary ±0.2 L. Indirectness −0: direct eligible contrast. Publication bias −0: no additional directional evidence; absence not established. | Very low |
| Higher vs lower fluid-replacement dose | Abdominal discomfort | 1 (8) | 1.80 (1.14 to 2.46) AU | Risk of bias −2: 100.0% high-risk and 0.0% some-concerns random-effects weight. Inconsistency −0: Single study; between-study inconsistency cannot be evaluated. Imprecision −2: N/benchmark=0.04; boundary ±1.0 AU. Indirectness −0: direct eligible contrast. Publication bias −0: no additional directional evidence; absence not established. | Very low |
| Higher vs lower fluid-replacement dose | Body mass loss (%) | 3 (26) | -1.10 (-2.54 to 0.34) percentage points | Risk of bias −1: 0.0% high-risk and 100.0% some-concerns random-effects weight. Inconsistency −1: I²=94.0%; prediction interval -9.28 to 7.09; opposite-signed study estimates: no. Imprecision −2: N/benchmark=0.13; boundary ±1.0 percentage points. Indirectness −0: direct eligible contrast. Publication bias −0: no additional directional evidence; absence not established. | Very low |
| Higher vs lower fluid-replacement dose | Core temperature | 2 (17) | -0.30 (-1.20 to 0.60) °C | Risk of bias −1: 0.0% high-risk and 100.0% some-concerns random-effects weight. Inconsistency −0: I²=0.0%; prediction interval Not estimable (k<3); opposite-signed study estimates: no. Imprecision −2: N/benchmark=0.09; boundary ±0.3 °C. Indirectness −0: direct eligible contrast. Publication bias −0: no additional directional evidence; absence not established. | Very low |
| Higher vs lower fluid-replacement dose | Heart rate | 1 (8) | 7.00 (1.12 to 12.88) beats/min | Risk of bias −1: 0.0% high-risk and 100.0% some-concerns random-effects weight. Inconsistency −0: Single study; between-study inconsistency cannot be evaluated. Imprecision −2: N/benchmark=0.04; boundary ±5.0 beats/min. Indirectness −0: direct eligible contrast. Publication bias −0: no additional directional evidence; absence not established. | Very low |
| Higher vs lower fluid-replacement dose | Plasma volume change (%) | 2 (16) | 5.18 (-7.21 to 17.57) percentage points | Risk of bias −1: 0.0% high-risk and 100.0% some-concerns random-effects weight. Inconsistency −0: I²=0.0%; prediction interval Not estimable (k<3); opposite-signed study estimates: no. Imprecision −2: N/benchmark=0.08; boundary ±3.0 percentage points. Indirectness −0: direct eligible contrast. Publication bias −0: no additional directional evidence; absence not established. | Very low |
| Higher vs lower fluid-replacement dose | Time-to-exhaustion performance | 1 (7) | 32.3% (-37.2% to 178.4%) | Risk of bias −1: 0.0% high-risk and 100.0% some-concerns random-effects weight. Inconsistency −0: Single study; between-study inconsistency cannot be evaluated. Imprecision −2: N/benchmark=0.02; boundary ±2%. Indirectness −0: direct eligible contrast. Publication bias −0: no additional directional evidence; absence not established. | Very low |
| Higher vs lower fluid-replacement dose | Time-trial/fixed-duration performance | 3 (25) | 2.7% (-8.8% to 15.6%) | Risk of bias −1: 0.0% high-risk and 100.0% some-concerns random-effects weight. Inconsistency −1: I²=67.4%; prediction interval -43.3% to 85.9%; opposite-signed study estimates: no. Imprecision −2: N/benchmark=0.08; boundary ±2%. Indirectness −0: direct eligible contrast. Publication bias −0: no additional directional evidence; absence not established. | Very low |
| Higher vs lower fluid-replacement dose | Urine volume (L) | 3 (24) | 0.13 (-0.05 to 0.30) L | Risk of bias −1: 0.0% high-risk and 100.0% some-concerns random-effects weight. Inconsistency −0: I²=0.0%; prediction interval -0.39 to 0.64; opposite-signed study estimates: no. Imprecision −2: N/benchmark=0.12; boundary ±0.2 L. Indirectness −0: direct eligible contrast. Publication bias −0: no additional directional evidence; absence not established. | Very low |
| Higher vs lower sodium/electrolyte dose | Body mass loss (%) | 1 (11) | -0.23 (-0.48 to 0.02) percentage points | Risk of bias −1: 0.0% high-risk and 100.0% some-concerns random-effects weight. Inconsistency −0: Single study; between-study inconsistency cannot be evaluated. Imprecision −2: N/benchmark=0.06; boundary ±1.0 percentage points. Indirectness −0: direct eligible contrast. Publication bias −0: no additional directional evidence; absence not established. | Very low |
| Higher vs lower sodium/electrolyte dose | Heart rate | 1 (6) | -2.00 (-5.39 to 1.39) beats/min | Risk of bias −1: 0.0% high-risk and 100.0% some-concerns random-effects weight. Inconsistency −0: Single study; between-study inconsistency cannot be evaluated. Imprecision −2: N/benchmark=0.03; boundary ±5.0 beats/min. Indirectness −0: direct eligible contrast. Publication bias −0: no additional directional evidence; absence not established. | Very low |
| Higher vs lower sodium/electrolyte dose | Plasma sodium change | 1 (11) | 2.30 (0.02 to 4.52) mmol/L | Risk of bias −1: 0.0% high-risk and 100.0% some-concerns random-effects weight. Inconsistency −0: Single study; between-study inconsistency cannot be evaluated. Imprecision −2: N/benchmark=0.06; boundary ±2.0 mmol/L. Indirectness −0: direct eligible contrast. Publication bias −0: no additional directional evidence; absence not established. | Very low |
| Higher vs lower sodium/electrolyte dose | Plasma sodium concentration | 1 (6) | 4.00 (2.04 to 5.96) mmol/L | Risk of bias −1: 0.0% high-risk and 100.0% some-concerns random-effects weight. Inconsistency −0: Single study; between-study inconsistency cannot be evaluated. Imprecision −2: N/benchmark=0.03; boundary ±2.0 mmol/L. Indirectness −0: direct eligible contrast. Publication bias −0: no additional directional evidence; absence not established. | Very low |
| Higher vs lower sodium/electrolyte dose | Plasma volume change (%) | 2 (17) | 1.78 (-5.24 to 8.81) percentage points | Risk of bias −1: 0.0% high-risk and 100.0% some-concerns random-effects weight. Inconsistency −0: I²=0.0%; prediction interval Not estimable (k<3); opposite-signed study estimates: no. Imprecision −2: N/benchmark=0.09; boundary ±3.0 percentage points. Indirectness −0: direct eligible contrast. Publication bias −0: no additional directional evidence; absence not established. | Very low |
| Ad libitum drinking vs thirst-driven drinking | Body mass loss (%) | 1 (10) | -0.30 (-0.96 to 0.36) percentage points | Risk of bias −1: 0.0% high-risk and 100.0% some-concerns random-effects weight. Inconsistency −0: Single study; between-study inconsistency cannot be evaluated. Imprecision −2: N/benchmark=0.05; boundary ±1.0 percentage points. Indirectness −0: direct eligible contrast. Publication bias −0: no additional directional evidence; absence not established. | Very low |
| Ad libitum drinking vs thirst-driven drinking | Core temperature | 1 (10) | -0.20 (-0.61 to 0.21) °C | Risk of bias −1: 0.0% high-risk and 100.0% some-concerns random-effects weight. Inconsistency −0: Single study; between-study inconsistency cannot be evaluated. Imprecision −2: N/benchmark=0.05; boundary ±0.3 °C. Indirectness −0: direct eligible contrast. Publication bias −0: no additional directional evidence; absence not established. | Very low |
| Ad libitum drinking vs thirst-driven drinking | Heart rate | 1 (9) | -5.00 (-18.12 to 8.12) beats/min | Risk of bias −1: 0.0% high-risk and 100.0% some-concerns random-effects weight. Inconsistency −0: Single study; between-study inconsistency cannot be evaluated. Imprecision −2: N/benchmark=0.04; boundary ±5.0 beats/min. Indirectness −0: direct eligible contrast. Publication bias −0: no additional directional evidence; absence not established. | Very low |
| Ad libitum drinking vs thirst-driven drinking | Rating of perceived exertion (Borg 6-20) | 1 (10) | -1.00 (-2.64 to 0.64) AU | Risk of bias −2: 100.0% high-risk and 0.0% some-concerns random-effects weight. Inconsistency −0: Single study; between-study inconsistency cannot be evaluated. Imprecision −2: N/benchmark=0.05; boundary ±1.0 AU. Indirectness −0: direct eligible contrast. Publication bias −0: no additional directional evidence; absence not established. | Very low |
| Ad libitum drinking vs thirst-driven drinking | Urine volume (L) | 1 (10) | 0.02 (-0.05 to 0.10) L | Risk of bias −1: 0.0% high-risk and 100.0% some-concerns random-effects weight. Inconsistency −0: Single study; between-study inconsistency cannot be evaluated. Imprecision −2: N/benchmark=0.05; boundary ±0.2 L. Indirectness −0: direct eligible contrast. Publication bias −0: no additional directional evidence; absence not established. | Very low |
| Distributed vs front-loaded fluid timing | Core temperature | 1 (7) | -0.10 (-0.30 to 0.10) °C | Risk of bias −1: 0.0% high-risk and 100.0% some-concerns random-effects weight. Inconsistency −0: Single study; between-study inconsistency cannot be evaluated. Imprecision −2: N/benchmark=0.04; boundary ±0.3 °C. Indirectness −0: direct eligible contrast. Publication bias −0: no additional directional evidence; absence not established. | Very low |
| Distributed vs front-loaded fluid timing | Heart rate | 1 (7) | 1.00 (-4.88 to 6.88) beats/min | Risk of bias −1: 0.0% high-risk and 100.0% some-concerns random-effects weight. Inconsistency −0: Single study; between-study inconsistency cannot be evaluated. Imprecision −2: N/benchmark=0.04; boundary ±5.0 beats/min. Indirectness −0: direct eligible contrast. Publication bias −0: no additional directional evidence; absence not established. | Very low |

**Table S8. Sensitivity, subgroup and alternative-selection results.**

Panel A reports targeted restrictions and correlation/selection alternatives. Each row is a separate post hoc exploratory analysis, not an independent confirmatory test. Study counts and sample sizes are recomputed after each restriction. Empty or one-study restrictions do not generate pooled estimates. The complete leave-one-out output, including non-estimable cases, is in outputs/sensitivity_results.csv.

| **Comparison** | **Outcome** | **Restriction / alternative** | **Studies (N)** | **Effect (95% CI)** | **I² (%)** |
| --- | --- | --- | --- | --- | --- |
| Oral hydration vs no or minimal fluid | Body mass loss (%) | Within-participant r=0.25 | 7 (72) | -2.63 (-3.58 to -1.69) percentage points | 97.5 |
| Oral hydration vs no or minimal fluid | Core temperature | Within-participant r=0.25 | 9 (80) | -0.39 (-0.75 to -0.03) °C | 82.0 |
| Oral hydration vs no or minimal fluid | Heart rate | Within-participant r=0.25 | 12 (115) | -7.00 (-11.43 to -2.58) beats/min | 76.6 |
| Oral hydration vs no or minimal fluid | Plasma sodium concentration | Within-participant r=0.25 | 4 (29) | -4.38 (-10.16 to 1.41) mmol/L | 87.9 |
| Oral hydration vs no or minimal fluid | Plasma volume change (%) | Within-participant r=0.25 | 7 (60) | 3.27 (0.33 to 6.20) percentage points | 75.3 |
| Oral hydration vs no or minimal fluid | Rating of perceived exertion (Borg 6-20) | Within-participant r=0.25 | 3 (39) | -0.55 (-2.15 to 1.06) AU | 12.8 |
| Oral hydration vs no or minimal fluid | Serum/plasma osmolality | Within-participant r=0.25 | 3 (26) | -12.65 (-34.52 to 9.21) mOsm/kg | 89.1 |
| Oral hydration vs no or minimal fluid | Time-to-exhaustion performance | Within-participant r=0.25 | 3 (21) | 33.6% (11.3% to 60.5%) | 0.0 |
| Oral hydration vs no or minimal fluid | Time-trial/fixed-duration performance | Within-participant r=0.25 | 4 (30) | -0.2% (-3.9% to 3.7%) | 0.0 |
| Oral hydration vs no or minimal fluid | Urine volume (L) | Within-participant r=0.25 | 4 (30) | 0.06 (-0.05 to 0.16) L | 1.7 |
| Programmed or individualized vs self-regulated drinking | Abdominal discomfort | Within-participant r=0.25 | 2 (17) | 0.22 (-1.69 to 2.14) AU | 0.0 |
| Programmed or individualized vs self-regulated drinking | Body mass loss (%) | Within-participant r=0.25 | 4 (40) | -1.49 (-1.93 to -1.05) percentage points | 44.0 |
| Programmed or individualized vs self-regulated drinking | Core temperature | Within-participant r=0.25 | 3 (31) | -0.23 (-0.65 to 0.19) °C | 0.0 |
| Programmed or individualized vs self-regulated drinking | Heart rate | Within-participant r=0.25 | 2 (19) | -3.24 (-23.80 to 17.32) beats/min | 0.0 |
| Programmed or individualized vs self-regulated drinking | Time-trial/fixed-duration performance | Within-participant r=0.25 | 3 (27) | 0.7% (-5.5% to 7.4%) | 0.0 |
| Programmed or individualized vs self-regulated drinking | Urine volume (L) | Within-participant r=0.25 | 2 (17) | 0.36 (-4.77 to 5.49) L | 77.8 |
| Higher vs lower fluid-replacement dose | Body mass loss (%) | Within-participant r=0.25 | 3 (26) | -1.10 (-2.57 to 0.36) percentage points | 91.2 |
| Higher vs lower fluid-replacement dose | Core temperature | Within-participant r=0.25 | 2 (17) | -0.30 (-1.22 to 0.62) °C | 0.0 |
| Higher vs lower fluid-replacement dose | Plasma volume change (%) | Within-participant r=0.25 | 2 (16) | 5.15 (-8.02 to 18.31) percentage points | 0.0 |
| Higher vs lower fluid-replacement dose | Time-trial/fixed-duration performance | Within-participant r=0.25 | 3 (25) | 2.8% (-8.9% to 16.0%) | 65.4 |
| Higher vs lower fluid-replacement dose | Urine volume (L) | Within-participant r=0.25 | 3 (24) | 0.13 (-0.08 to 0.33) L | 0.0 |
| Higher vs lower sodium/electrolyte dose | Plasma volume change (%) | Within-participant r=0.25 | 2 (17) | 1.85 (-5.38 to 9.08) percentage points | 0.0 |
| Oral hydration vs no or minimal fluid | Body mass loss (%) | Within-participant r=0.75 | 7 (72) | -2.63 (-3.57 to -1.69) percentage points | 99.0 |
| Oral hydration vs no or minimal fluid | Core temperature | Within-participant r=0.75 | 9 (80) | -0.38 (-0.74 to -0.01) °C | 92.8 |
| Oral hydration vs no or minimal fluid | Heart rate | Within-participant r=0.75 | 12 (115) | -6.58 (-10.80 to -2.37) beats/min | 90.1 |
| Oral hydration vs no or minimal fluid | Plasma sodium concentration | Within-participant r=0.75 | 4 (29) | -4.37 (-10.20 to 1.45) mmol/L | 95.5 |
| Oral hydration vs no or minimal fluid | Plasma volume change (%) | Within-participant r=0.75 | 7 (60) | 3.29 (0.51 to 6.07) percentage points | 90.5 |
| Oral hydration vs no or minimal fluid | Rating of perceived exertion (Borg 6-20) | Within-participant r=0.75 | 3 (39) | -0.55 (-2.06 to 0.96) AU | 64.9 |
| Oral hydration vs no or minimal fluid | Serum/plasma osmolality | Within-participant r=0.75 | 3 (26) | -12.85 (-33.81 to 8.11) mOsm/kg | 95.9 |
| Oral hydration vs no or minimal fluid | Time-to-exhaustion performance | Within-participant r=0.75 | 3 (21) | 38.3% (5.6% to 81.1%) | 15.5 |
| Oral hydration vs no or minimal fluid | Time-trial/fixed-duration performance | Within-participant r=0.75 | 4 (30) | -0.1% (-3.5% to 3.5%) | 50.5 |
| Oral hydration vs no or minimal fluid | Urine volume (L) | Within-participant r=0.75 | 4 (30) | 0.06 (-0.05 to 0.16) L | 56.7 |
| Programmed or individualized vs self-regulated drinking | Abdominal discomfort | Within-participant r=0.75 | 2 (17) | 0.23 (-1.19 to 1.65) AU | 0.0 |
| Programmed or individualized vs self-regulated drinking | Body mass loss (%) | Within-participant r=0.75 | 4 (40) | -1.45 (-1.98 to -0.92) percentage points | 80.8 |
| Programmed or individualized vs self-regulated drinking | Core temperature | Within-participant r=0.75 | 3 (31) | -0.23 (-0.48 to 0.02) °C | 0.0 |
| Programmed or individualized vs self-regulated drinking | Heart rate | Within-participant r=0.75 | 2 (19) | -3.25 (-15.43 to 8.93) beats/min | 0.0 |
| Programmed or individualized vs self-regulated drinking | Time-trial/fixed-duration performance | Within-participant r=0.75 | 3 (27) | 0.7% (-3.0% to 4.6%) | 0.0 |
| Programmed or individualized vs self-regulated drinking | Urine volume (L) | Within-participant r=0.75 | 2 (17) | 0.42 (-4.82 to 5.66) L | 92.1 |
| Higher vs lower fluid-replacement dose | Body mass loss (%) | Within-participant r=0.75 | 3 (26) | -1.09 (-2.50 to 0.32) percentage points | 96.9 |
| Higher vs lower fluid-replacement dose | Core temperature | Within-participant r=0.75 | 2 (17) | -0.30 (-1.14 to 0.54) °C | 0.0 |
| Higher vs lower fluid-replacement dose | Plasma volume change (%) | Within-participant r=0.75 | 2 (16) | 5.22 (-5.91 to 16.35) percentage points | 0.0 |
| Higher vs lower fluid-replacement dose | Time-trial/fixed-duration performance | Within-participant r=0.75 | 3 (25) | 2.5% (-8.6% to 15.0%) | 69.4 |
| Higher vs lower fluid-replacement dose | Urine volume (L) | Within-participant r=0.75 | 3 (24) | 0.13 (-0.03 to 0.29) L | 0.0 |
| Higher vs lower sodium/electrolyte dose | Plasma volume change (%) | Within-participant r=0.75 | 2 (17) | 1.56 (-6.24 to 9.37) percentage points | 23.8 |
| Oral hydration vs no or minimal fluid | Body mass loss (%) | Between-condition correlation=0.0 | 7 (72) | -2.63 (-3.57 to -1.69) percentage points | 98.3 |
| Oral hydration vs no or minimal fluid | Core temperature | Between-condition correlation=0.0 | 9 (80) | -0.38 (-0.74 to -0.02) °C | 87.6 |
| Oral hydration vs no or minimal fluid | Heart rate | Between-condition correlation=0.0 | 12 (115) | -6.79 (-11.11 to -2.46) beats/min | 83.6 |
| Oral hydration vs no or minimal fluid | Plasma sodium concentration | Between-condition correlation=0.0 | 4 (29) | -4.37 (-10.18 to 1.43) mmol/L | 91.7 |
| Oral hydration vs no or minimal fluid | Plasma volume change (%) | Between-condition correlation=0.0 | 7 (60) | 3.28 (0.42 to 6.14) percentage points | 82.7 |
| Oral hydration vs no or minimal fluid | Rating of perceived exertion (Borg 6-20) | Between-condition correlation=0.0 | 3 (39) | -0.54 (-2.11 to 1.03) AU | 38.8 |
| Oral hydration vs no or minimal fluid | Serum/plasma osmolality | Between-condition correlation=0.0 | 3 (26) | -12.75 (-34.19 to 8.69) mOsm/kg | 92.5 |
| Oral hydration vs no or minimal fluid | Time-to-exhaustion performance | Between-condition correlation=0.0 | 3 (21) | 34.1% (11.9% to 60.7%) | 0.0 |
| Oral hydration vs no or minimal fluid | Time-trial/fixed-duration performance | Between-condition correlation=0.0 | 4 (30) | -0.2% (-3.6% to 3.4%) | 6.7 |
| Oral hydration vs no or minimal fluid | Urine volume (L) | Between-condition correlation=0.0 | 4 (30) | 0.06 (-0.05 to 0.16) L | 28.3 |
| Programmed or individualized vs self-regulated drinking | Abdominal discomfort | Between-condition correlation=0.0 | 2 (17) | 0.23 (-1.46 to 1.91) AU | 0.0 |
| Programmed or individualized vs self-regulated drinking | Body mass loss (%) | Between-condition correlation=0.0 | 4 (40) | -1.47 (-1.96 to -0.99) percentage points | 62.4 |
| Programmed or individualized vs self-regulated drinking | Core temperature | Between-condition correlation=0.0 | 3 (31) | -0.23 (-0.58 to 0.12) °C | 0.0 |
| Programmed or individualized vs self-regulated drinking | Heart rate | Between-condition correlation=0.0 | 2 (19) | -3.24 (-20.14 to 13.65) beats/min | 0.0 |
| Programmed or individualized vs self-regulated drinking | Time-trial/fixed-duration performance | Between-condition correlation=0.0 | 3 (27) | 0.7% (-4.4% to 6.2%) | 0.0 |
| Programmed or individualized vs self-regulated drinking | Urine volume (L) | Between-condition correlation=0.0 | 2 (17) | 0.39 (-4.81 to 5.59) L | 85.0 |
| Higher vs lower fluid-replacement dose | Body mass loss (%) | Between-condition correlation=0.0 | 3 (26) | -1.10 (-2.54 to 0.34) percentage points | 94.0 |
| Higher vs lower fluid-replacement dose | Core temperature | Between-condition correlation=0.0 | 2 (17) | -0.30 (-1.20 to 0.60) °C | 0.0 |
| Higher vs lower fluid-replacement dose | Plasma volume change (%) | Between-condition correlation=0.0 | 2 (16) | 5.18 (-7.21 to 17.57) percentage points | 0.0 |
| Higher vs lower fluid-replacement dose | Time-trial/fixed-duration performance | Between-condition correlation=0.0 | 3 (25) | 2.7% (-8.8% to 15.6%) | 67.4 |
| Higher vs lower fluid-replacement dose | Urine volume (L) | Between-condition correlation=0.0 | 3 (24) | 0.13 (-0.05 to 0.30) L | 0.0 |
| Higher vs lower sodium/electrolyte dose | Plasma volume change (%) | Between-condition correlation=0.0 | 2 (17) | 1.78 (-5.24 to 8.81) percentage points | 0.0 |
| Oral hydration vs no or minimal fluid | Body mass loss (%) | Between-condition correlation=0.9 | 7 (72) | -2.63 (-3.58 to -1.69) percentage points | 98.2 |
| Oral hydration vs no or minimal fluid | Core temperature | Between-condition correlation=0.9 | 9 (80) | -0.39 (-0.75 to -0.02) °C | 87.2 |
| Oral hydration vs no or minimal fluid | Heart rate | Between-condition correlation=0.9 | 12 (115) | -6.85 (-11.20 to -2.50) beats/min | 83.2 |
| Oral hydration vs no or minimal fluid | Plasma sodium concentration | Between-condition correlation=0.9 | 4 (29) | -4.37 (-10.18 to 1.43) mmol/L | 91.7 |
| Oral hydration vs no or minimal fluid | Plasma volume change (%) | Between-condition correlation=0.9 | 7 (60) | 3.28 (0.42 to 6.14) percentage points | 82.7 |
| Oral hydration vs no or minimal fluid | Rating of perceived exertion (Borg 6-20) | Between-condition correlation=0.9 | 3 (39) | -0.54 (-2.11 to 1.03) AU | 38.8 |
| Oral hydration vs no or minimal fluid | Serum/plasma osmolality | Between-condition correlation=0.9 | 3 (26) | -12.75 (-34.19 to 8.69) mOsm/kg | 92.5 |
| Oral hydration vs no or minimal fluid | Time-to-exhaustion performance | Between-condition correlation=0.9 | 3 (21) | 34.1% (11.9% to 60.7%) | 0.0 |
| Oral hydration vs no or minimal fluid | Time-trial/fixed-duration performance | Between-condition correlation=0.9 | 4 (30) | -0.1% (-3.6% to 3.6%) | 0.0 |
| Oral hydration vs no or minimal fluid | Urine volume (L) | Between-condition correlation=0.9 | 4 (30) | 0.06 (-0.05 to 0.16) L | 28.3 |
| Programmed or individualized vs self-regulated drinking | Abdominal discomfort | Between-condition correlation=0.9 | 2 (17) | 0.23 (-1.46 to 1.91) AU | 0.0 |
| Programmed or individualized vs self-regulated drinking | Body mass loss (%) | Between-condition correlation=0.9 | 4 (40) | -1.47 (-1.96 to -0.99) percentage points | 62.4 |
| Programmed or individualized vs self-regulated drinking | Core temperature | Between-condition correlation=0.9 | 3 (31) | -0.23 (-0.58 to 0.12) °C | 0.0 |
| Programmed or individualized vs self-regulated drinking | Heart rate | Between-condition correlation=0.9 | 2 (19) | -3.24 (-20.14 to 13.65) beats/min | 0.0 |
| Programmed or individualized vs self-regulated drinking | Time-trial/fixed-duration performance | Between-condition correlation=0.9 | 3 (27) | 0.7% (-4.4% to 6.2%) | 0.0 |
| Programmed or individualized vs self-regulated drinking | Urine volume (L) | Between-condition correlation=0.9 | 2 (17) | 0.39 (-4.81 to 5.59) L | 85.0 |
| Higher vs lower fluid-replacement dose | Body mass loss (%) | Between-condition correlation=0.9 | 3 (26) | -1.10 (-2.54 to 0.34) percentage points | 94.0 |
| Higher vs lower fluid-replacement dose | Core temperature | Between-condition correlation=0.9 | 2 (17) | -0.30 (-1.20 to 0.60) °C | 0.0 |
| Higher vs lower fluid-replacement dose | Plasma volume change (%) | Between-condition correlation=0.9 | 2 (16) | 5.18 (-7.21 to 17.57) percentage points | 0.0 |
| Higher vs lower fluid-replacement dose | Time-trial/fixed-duration performance | Between-condition correlation=0.9 | 3 (25) | 2.7% (-8.8% to 15.6%) | 67.4 |
| Higher vs lower fluid-replacement dose | Urine volume (L) | Between-condition correlation=0.9 | 3 (24) | 0.13 (-0.05 to 0.30) L | 0.0 |
| Higher vs lower sodium/electrolyte dose | Plasma volume change (%) | Between-condition correlation=0.9 | 2 (17) | 1.78 (-5.24 to 8.81) percentage points | 0.0 |
| Oral hydration vs no or minimal fluid | Body mass loss (%) | Exclude high-risk results | 7 (72) | -2.63 (-3.58 to -1.69) percentage points | 98.3 |
| Oral hydration vs no or minimal fluid | Core temperature | Exclude high-risk results | 9 (80) | -0.38 (-0.74 to -0.02) °C | 87.3 |
| Oral hydration vs no or minimal fluid | Heart rate | Exclude high-risk results | 12 (115) | -6.82 (-11.16 to -2.48) beats/min | 83.3 |
| Oral hydration vs no or minimal fluid | Plasma sodium concentration | Exclude high-risk results | 4 (29) | -4.37 (-10.18 to 1.43) mmol/L | 91.7 |
| Oral hydration vs no or minimal fluid | Plasma volume change (%) | Exclude high-risk results | 7 (60) | 3.28 (0.42 to 6.14) percentage points | 82.7 |
| Oral hydration vs no or minimal fluid | Serum/plasma osmolality | Exclude high-risk results | 3 (26) | -12.75 (-34.19 to 8.69) mOsm/kg | 92.5 |
| Oral hydration vs no or minimal fluid | Time-to-exhaustion performance | Exclude high-risk results | 3 (21) | 34.1% (11.9% to 60.7%) | 0.0 |
| Oral hydration vs no or minimal fluid | Time-trial/fixed-duration performance | Exclude high-risk results | 4 (30) | -0.1% (-3.6% to 3.5%) | 1.8 |
| Oral hydration vs no or minimal fluid | Urine volume (L) | Exclude high-risk results | 4 (30) | 0.06 (-0.05 to 0.16) L | 28.3 |
| Programmed or individualized vs self-regulated drinking | Body mass loss (%) | Exclude high-risk results | 4 (40) | -1.47 (-1.96 to -0.99) percentage points | 62.4 |
| Programmed or individualized vs self-regulated drinking | Core temperature | Exclude high-risk results | 3 (31) | -0.23 (-0.58 to 0.12) °C | 0.0 |
| Programmed or individualized vs self-regulated drinking | Heart rate | Exclude high-risk results | 2 (19) | -3.24 (-20.14 to 13.65) beats/min | 0.0 |
| Programmed or individualized vs self-regulated drinking | Time-trial/fixed-duration performance | Exclude high-risk results | 3 (27) | 0.7% (-4.4% to 6.2%) | 0.0 |
| Programmed or individualized vs self-regulated drinking | Urine volume (L) | Exclude high-risk results | 2 (17) | 0.39 (-4.81 to 5.59) L | 85.0 |
| Higher vs lower fluid-replacement dose | Body mass loss (%) | Exclude high-risk results | 3 (26) | -1.10 (-2.54 to 0.34) percentage points | 94.0 |
| Higher vs lower fluid-replacement dose | Core temperature | Exclude high-risk results | 2 (17) | -0.30 (-1.20 to 0.60) °C | 0.0 |
| Higher vs lower fluid-replacement dose | Plasma volume change (%) | Exclude high-risk results | 2 (16) | 5.18 (-7.21 to 17.57) percentage points | 0.0 |
| Higher vs lower fluid-replacement dose | Time-trial/fixed-duration performance | Exclude high-risk results | 3 (25) | 2.7% (-8.8% to 15.6%) | 67.4 |
| Higher vs lower fluid-replacement dose | Urine volume (L) | Exclude high-risk results | 3 (24) | 0.13 (-0.05 to 0.30) L | 0.0 |
| Higher vs lower sodium/electrolyte dose | Plasma volume change (%) | Exclude high-risk results | 2 (17) | 1.78 (-5.24 to 8.81) percentage points | 0.0 |
| Oral hydration vs no or minimal fluid | Body mass loss (%) | Exclude exactly 60-min trials | 5 (43) | -2.94 (-4.22 to -1.66) percentage points | 97.4 |
| Oral hydration vs no or minimal fluid | Core temperature | Exclude exactly 60-min trials | 6 (45) | -0.39 (-1.02 to 0.23) °C | 91.3 |
| Oral hydration vs no or minimal fluid | Heart rate | Exclude exactly 60-min trials | 8 (71) | -9.02 (-15.18 to -2.87) beats/min | 74.3 |
| Oral hydration vs no or minimal fluid | Plasma sodium concentration | Exclude exactly 60-min trials | 3 (21) | -4.35 (-15.55 to 6.84) mmol/L | 94.5 |
| Oral hydration vs no or minimal fluid | Plasma volume change (%) | Exclude exactly 60-min trials | 6 (51) | 3.32 (-0.05 to 6.69) percentage points | 85.6 |
| Oral hydration vs no or minimal fluid | Serum/plasma osmolality | Exclude exactly 60-min trials | 2 (18) | -18.54 (-47.40 to 10.32) mOsm/kg | 2.2 |
| Oral hydration vs no or minimal fluid | Time-to-exhaustion performance | Exclude exactly 60-min trials | 3 (21) | 34.1% (11.9% to 60.7%) | 0.0 |
| Oral hydration vs no or minimal fluid | Urine volume (L) | Exclude exactly 60-min trials | 2 (13) | 0.09 (-0.52 to 0.69) L | 0.0 |
| Programmed or individualized vs self-regulated drinking | Body mass loss (%) | Exclude exactly 60-min trials | 3 (31) | -1.39 (-2.36 to -0.43) percentage points | 66.2 |
| Programmed or individualized vs self-regulated drinking | Core temperature | Exclude exactly 60-min trials | 3 (31) | -0.23 (-0.58 to 0.12) °C | 0.0 |
| Programmed or individualized vs self-regulated drinking | Time-trial/fixed-duration performance | Exclude exactly 60-min trials | 2 (18) | 1.5% (-17.2% to 24.4%) | 0.0 |
| Higher vs lower fluid-replacement dose | Body mass loss (%) | Exclude exactly 60-min trials | 2 (18) | -1.34 (-6.40 to 3.73) percentage points | 88.1 |
| Higher vs lower fluid-replacement dose | Core temperature | Exclude exactly 60-min trials | 2 (17) | -0.30 (-1.20 to 0.60) °C | 0.0 |
| Higher vs lower fluid-replacement dose | Plasma volume change (%) | Exclude exactly 60-min trials | 2 (16) | 5.18 (-7.21 to 17.57) percentage points | 0.0 |
| Higher vs lower fluid-replacement dose | Time-trial/fixed-duration performance | Exclude exactly 60-min trials | 2 (17) | 4.6% (-39.9% to 81.8%) | 81.1 |
| Higher vs lower fluid-replacement dose | Urine volume (L) | Exclude exactly 60-min trials | 3 (24) | 0.13 (-0.05 to 0.30) L | 0.0 |
| Higher vs lower sodium/electrolyte dose | Plasma volume change (%) | Exclude exactly 60-min trials | 2 (17) | 1.78 (-5.24 to 8.81) percentage points | 0.0 |
| Oral hydration vs no or minimal fluid | Body mass loss (%) | Exclude preload-plus-performance-test protocols | 6 (65) | -2.55 (-3.70 to -1.40) percentage points | 98.3 |
| Oral hydration vs no or minimal fluid | Core temperature | Exclude preload-plus-performance-test protocols | 8 (74) | -0.46 (-0.83 to -0.10) °C | 84.8 |
| Oral hydration vs no or minimal fluid | Heart rate | Exclude preload-plus-performance-test protocols | 11 (109) | -6.90 (-11.56 to -2.24) beats/min | 84.7 |
| Oral hydration vs no or minimal fluid | Plasma sodium concentration | Exclude preload-plus-performance-test protocols | 4 (29) | -4.37 (-10.18 to 1.43) mmol/L | 91.7 |
| Oral hydration vs no or minimal fluid | Plasma volume change (%) | Exclude preload-plus-performance-test protocols | 6 (53) | 3.01 (-0.38 to 6.41) percentage points | 84.8 |
| Oral hydration vs no or minimal fluid | Rating of perceived exertion (Borg 6-20) | Exclude preload-plus-performance-test protocols | 3 (39) | -0.54 (-2.11 to 1.03) AU | 38.8 |
| Oral hydration vs no or minimal fluid | Serum/plasma osmolality | Exclude preload-plus-performance-test protocols | 3 (26) | -12.75 (-34.19 to 8.69) mOsm/kg | 92.5 |
| Oral hydration vs no or minimal fluid | Time-trial/fixed-duration performance | Exclude preload-plus-performance-test protocols | 4 (30) | -0.1% (-3.6% to 3.5%) | 1.8 |
| Oral hydration vs no or minimal fluid | Urine volume (L) | Exclude preload-plus-performance-test protocols | 3 (23) | 0.05 (-0.09 to 0.19) L | 33.8 |
| Programmed or individualized vs self-regulated drinking | Body mass loss (%) | Exclude preload-plus-performance-test protocols | 3 (32) | -1.54 (-2.16 to -0.92) percentage points | 63.4 |
| Programmed or individualized vs self-regulated drinking | Core temperature | Exclude preload-plus-performance-test protocols | 2 (23) | -0.25 (-1.50 to 1.00) °C | 0.0 |
| Programmed or individualized vs self-regulated drinking | Heart rate | Exclude preload-plus-performance-test protocols | 2 (19) | -3.24 (-20.14 to 13.65) beats/min | 0.0 |
| Programmed or individualized vs self-regulated drinking | Time-trial/fixed-duration performance | Exclude preload-plus-performance-test protocols | 2 (19) | -0.1% (-17.8% to 21.4%) | 0.0 |
| Higher vs lower fluid-replacement dose | Time-trial/fixed-duration performance | Exclude preload-plus-performance-test protocols | 2 (14) | 0.4% (-14.4% to 17.8%) | 0.0 |
| Higher vs lower sodium/electrolyte dose | Plasma volume change (%) | Exclude preload-plus-performance-test protocols | 2 (17) | 1.78 (-5.24 to 8.81) percentage points | 0.0 |
| Oral hydration vs no or minimal fluid | Body mass loss (%) | Exclude carbohydrate co-ingestion contrast (Montain 1992a) | 6 (65) | -2.57 (-3.73 to -1.42) percentage points | 98.5 |
| Oral hydration vs no or minimal fluid | Core temperature | Exclude carbohydrate co-ingestion contrast (Montain 1992a) | 8 (73) | -0.33 (-0.72 to 0.07) °C | 84.9 |
| Oral hydration vs no or minimal fluid | Heart rate | Exclude carbohydrate co-ingestion contrast (Montain 1992a) | 11 (108) | -5.89 (-10.28 to -1.51) beats/min | 79.1 |
| Oral hydration vs no or minimal fluid | Plasma sodium concentration | Exclude carbohydrate co-ingestion contrast (Montain 1992a) | 4 (29) | -4.37 (-10.18 to 1.43) mmol/L | 91.7 |
| Oral hydration vs no or minimal fluid | Plasma volume change (%) | Exclude carbohydrate co-ingestion contrast (Montain 1992a) | 7 (60) | 3.28 (0.42 to 6.14) percentage points | 82.7 |
| Oral hydration vs no or minimal fluid | Rating of perceived exertion (Borg 6-20) | Exclude carbohydrate co-ingestion contrast (Montain 1992a) | 3 (39) | -0.54 (-2.11 to 1.03) AU | 38.8 |
| Oral hydration vs no or minimal fluid | Serum/plasma osmolality | Exclude carbohydrate co-ingestion contrast (Montain 1992a) | 3 (26) | -12.75 (-34.19 to 8.69) mOsm/kg | 92.5 |
| Oral hydration vs no or minimal fluid | Time-to-exhaustion performance | Exclude carbohydrate co-ingestion contrast (Montain 1992a) | 3 (21) | 34.1% (11.9% to 60.7%) | 0.0 |
| Oral hydration vs no or minimal fluid | Time-trial/fixed-duration performance | Exclude carbohydrate co-ingestion contrast (Montain 1992a) | 4 (30) | -0.1% (-3.6% to 3.5%) | 1.8 |
| Oral hydration vs no or minimal fluid | Urine volume (L) | Exclude carbohydrate co-ingestion contrast (Montain 1992a) | 4 (30) | 0.06 (-0.05 to 0.16) L | 28.3 |
| Programmed or individualized vs self-regulated drinking | Abdominal discomfort | Exclude carbohydrate co-ingestion contrast (Montain 1992a) | 2 (17) | 0.23 (-1.46 to 1.91) AU | 0.0 |
| Programmed or individualized vs self-regulated drinking | Body mass loss (%) | Exclude carbohydrate co-ingestion contrast (Montain 1992a) | 4 (40) | -1.47 (-1.96 to -0.99) percentage points | 62.4 |
| Programmed or individualized vs self-regulated drinking | Core temperature | Exclude carbohydrate co-ingestion contrast (Montain 1992a) | 3 (31) | -0.23 (-0.58 to 0.12) °C | 0.0 |
| Programmed or individualized vs self-regulated drinking | Heart rate | Exclude carbohydrate co-ingestion contrast (Montain 1992a) | 2 (19) | -3.24 (-20.14 to 13.65) beats/min | 0.0 |
| Programmed or individualized vs self-regulated drinking | Time-trial/fixed-duration performance | Exclude carbohydrate co-ingestion contrast (Montain 1992a) | 3 (27) | 0.7% (-4.4% to 6.2%) | 0.0 |
| Programmed or individualized vs self-regulated drinking | Urine volume (L) | Exclude carbohydrate co-ingestion contrast (Montain 1992a) | 2 (17) | 0.39 (-4.81 to 5.59) L | 85.0 |
| Higher vs lower fluid-replacement dose | Body mass loss (%) | Exclude carbohydrate co-ingestion contrast (Montain 1992a) | 3 (26) | -1.10 (-2.54 to 0.34) percentage points | 94.0 |
| Higher vs lower fluid-replacement dose | Core temperature | Exclude carbohydrate co-ingestion contrast (Montain 1992a) | 2 (17) | -0.30 (-1.20 to 0.60) °C | 0.0 |
| Higher vs lower fluid-replacement dose | Plasma volume change (%) | Exclude carbohydrate co-ingestion contrast (Montain 1992a) | 2 (16) | 5.18 (-7.21 to 17.57) percentage points | 0.0 |
| Higher vs lower fluid-replacement dose | Time-trial/fixed-duration performance | Exclude carbohydrate co-ingestion contrast (Montain 1992a) | 3 (25) | 2.7% (-8.8% to 15.6%) | 67.4 |
| Higher vs lower fluid-replacement dose | Urine volume (L) | Exclude carbohydrate co-ingestion contrast (Montain 1992a) | 3 (24) | 0.13 (-0.05 to 0.30) L | 0.0 |
| Higher vs lower sodium/electrolyte dose | Plasma volume change (%) | Exclude carbohydrate co-ingestion contrast (Montain 1992a) | 2 (17) | 1.78 (-5.24 to 8.81) percentage points | 0.0 |
| Oral hydration vs no or minimal fluid | Body mass loss (%) | True no-fluid comparator | 6 (65) | -2.33 (-3.05 to -1.60) percentage points | 97.2 |
| Oral hydration vs no or minimal fluid | Core temperature | True no-fluid comparator | 8 (73) | -0.29 (-0.57 to -0.01) °C | 80.8 |
| Oral hydration vs no or minimal fluid | Heart rate | True no-fluid comparator | 10 (94) | -5.74 (-10.13 to -1.34) beats/min | 81.0 |
| Oral hydration vs no or minimal fluid | Plasma sodium concentration | True no-fluid comparator | 3 (22) | -2.91 (-8.85 to 3.02) mmol/L | 83.7 |
| Oral hydration vs no or minimal fluid | Plasma volume change (%) | True no-fluid comparator | 6 (53) | 2.69 (-0.45 to 5.84) percentage points | 79.8 |
| Oral hydration vs no or minimal fluid | Rating of perceived exertion (Borg 6-20) | True no-fluid comparator | 3 (39) | -0.54 (-2.11 to 1.03) AU | 38.8 |
| Oral hydration vs no or minimal fluid | Serum/plasma osmolality | True no-fluid comparator | 2 (19) | -8.86 (-78.26 to 60.55) mOsm/kg | 82.9 |
| Oral hydration vs no or minimal fluid | Time-to-exhaustion performance | True no-fluid comparator | 3 (21) | 34.1% (11.9% to 60.7%) | 0.0 |
| Oral hydration vs no or minimal fluid | Time-trial/fixed-duration performance | True no-fluid comparator | 4 (30) | -0.1% (-3.6% to 3.5%) | 1.8 |
| Oral hydration vs no or minimal fluid | Urine volume (L) | True no-fluid comparator | 4 (30) | 0.06 (-0.05 to 0.16) L | 28.3 |
| Oral hydration vs no or minimal fluid | Body mass loss (%) | Environment: Hot | 3 (23) | -3.25 (-6.00 to -0.50) percentage points | 98.3 |
| Oral hydration vs no or minimal fluid | Core temperature | Environment: Hot | 4 (26) | -0.55 (-1.57 to 0.47) °C | 93.7 |
| Oral hydration vs no or minimal fluid | Heart rate | Environment: Hot | 6 (49) | -9.28 (-16.72 to -1.83) beats/min | 88.5 |
| Oral hydration vs no or minimal fluid | Plasma sodium concentration | Environment: Hot | 2 (13) | -6.50 (-38.27 to 25.27) mmol/L | 92.0 |
| Oral hydration vs no or minimal fluid | Plasma volume change (%) | Environment: Hot | 3 (22) | 4.61 (-0.51 to 9.73) percentage points | 39.6 |
| Oral hydration vs no or minimal fluid | Time-trial/fixed-duration performance | Environment: Hot | 2 (15) | 1.2% (-14.5% to 19.9%) | 0.0 |
| Oral hydration vs no or minimal fluid | Urine volume (L) | Environment: Hot | 2 (15) | 0.03 (-0.50 to 0.55) L | 34.9 |
| Oral hydration vs no or minimal fluid | Body mass loss (%) | Environment: Temperate | 4 (49) | -2.17 (-3.41 to -0.93) percentage points | 98.1 |
| Oral hydration vs no or minimal fluid | Core temperature | Environment: Temperate | 4 (47) | -0.30 (-0.58 to -0.02) °C | 25.0 |
| Oral hydration vs no or minimal fluid | Heart rate | Environment: Temperate | 5 (59) | -4.21 (-11.79 to 3.37) beats/min | 74.1 |
| Oral hydration vs no or minimal fluid | Plasma sodium concentration | Environment: Temperate | 2 (16) | -2.31 (-30.23 to 25.61) mmol/L | 91.7 |
| Oral hydration vs no or minimal fluid | Plasma volume change (%) | Environment: Temperate | 4 (38) | 2.53 (-3.15 to 8.20) percentage points | 87.2 |
| Oral hydration vs no or minimal fluid | Rating of perceived exertion (Borg 6-20) | Environment: Temperate | 3 (39) | -0.54 (-2.11 to 1.03) AU | 38.8 |
| Oral hydration vs no or minimal fluid | Serum/plasma osmolality | Environment: Temperate | 2 (19) | -8.86 (-78.26 to 60.55) mOsm/kg | 82.9 |
| Oral hydration vs no or minimal fluid | Time-to-exhaustion performance | Environment: Temperate | 2 (15) | 33.2% (-23.1% to 130.8%) | 0.0 |
| Oral hydration vs no or minimal fluid | Urine volume (L) | Environment: Temperate | 2 (15) | 0.10 (-0.43 to 0.63) L | 0.0 |
| Oral hydration vs no or minimal fluid | Core temperature | Rectal temperature only | 8 (73) | -0.29 (-0.57 to -0.01) °C | 80.8 |
| Programmed or individualized vs self-regulated drinking | Core temperature | Rectal temperature only | 2 (18) | -0.26 (-1.39 to 0.87) °C | 0.0 |
| Higher vs lower fluid-replacement dose | Core temperature | Rectal temperature only | 2 (17) | -0.30 (-1.20 to 0.60) °C | 0.0 |
| Oral hydration vs no or minimal fluid | Core temperature | Rectal or esophageal temperature | 9 (80) | -0.38 (-0.74 to -0.02) °C | 87.3 |
| Programmed or individualized vs self-regulated drinking | Core temperature | Rectal or esophageal temperature | 2 (18) | -0.26 (-1.39 to 0.87) °C | 0.0 |
| Higher vs lower fluid-replacement dose | Core temperature | Rectal or esophageal temperature | 2 (17) | -0.30 (-1.20 to 0.60) °C | 0.0 |
| Oral hydration vs no or minimal fluid | Heart rate | Heart rate: fixed-timepoint/end-exercise only | 8 (77) | -8.05 (-14.88 to -1.22) beats/min | 84.7 |
| Programmed or individualized vs self-regulated drinking | Abdominal discomfort | Regimen temperature: exclude change-score result | 2 (17) | 0.23 (-1.46 to 1.91) AU | 0.0 |
| Programmed or individualized vs self-regulated drinking | Body mass loss (%) | Regimen temperature: exclude change-score result | 3 (27) | -1.60 (-2.08 to -1.11) percentage points | 51.1 |
| Programmed or individualized vs self-regulated drinking | Core temperature | Regimen temperature: exclude change-score result | 2 (18) | -0.26 (-1.39 to 0.87) °C | 0.0 |
| Programmed or individualized vs self-regulated drinking | Heart rate | Regimen temperature: exclude change-score result | 2 (19) | -3.24 (-20.14 to 13.65) beats/min | 0.0 |
| Programmed or individualized vs self-regulated drinking | Time-trial/fixed-duration performance | Regimen temperature: exclude change-score result | 3 (27) | 0.7% (-4.4% to 6.2%) | 0.0 |
| Programmed or individualized vs self-regulated drinking | Urine volume (L) | Regimen temperature: exclude change-score result | 2 (17) | 0.39 (-4.81 to 5.59) L | 85.0 |
| Higher vs lower fluid-replacement dose | Time-trial/fixed-duration performance | Backx: mean power instead of distance | 3 (25) | 3.3% (-9.0% to 17.2%) | 63.4 |
| Oral hydration vs no or minimal fluid | Core temperature | Montain 1992a: esophageal instead of rectal temperature | 9 (78) | -0.37 (-0.72 to -0.02) °C | 85.9 |
| Programmed or individualized vs self-regulated drinking | Core temperature | Montain 1992a: esophageal instead of rectal temperature | 3 (31) | -0.23 (-0.58 to 0.12) °C | 0.0 |
| Higher vs lower fluid-replacement dose | Core temperature | Montain 1992a: esophageal instead of rectal temperature | 2 (17) | -0.30 (-1.20 to 0.60) °C | 0.0 |

Panel B. Separate within-study GLS dose slopes. No pooled slope is calculated because performance constructs differ. Target doses are exposures; achieved doses are not substituted without study-level uncertainty.

| **Study** | **Performance construct** | **N** | **Assumed r** | **Slope (95% CI), per 10 percentage points** |
| --- | --- | --- | --- | --- |
| Dugas_2009 | Time-trial completion time | 6 | 0.25 | 0.3% (-0.1% to 0.8%) per 10 percentage points |
| Dugas_2009 | Time-trial completion time | 6 | 0.5 | 0.3% (-0.1% to 0.7%) per 10 percentage points |
| Dugas_2009 | Time-trial completion time | 6 | 0.75 | 0.3% (0.0% to 0.6%) per 10 percentage points |
| McConell_1997 | Time to exhaustion | 7 | 0.25 | 6.6% (-2.4% to 16.5%) per 10 percentage points |
| McConell_1997 | Time to exhaustion | 7 | 0.5 | 6.6% (-1.0% to 14.9%) per 10 percentage points |
| McConell_1997 | Time to exhaustion | 7 | 0.75 | 6.7% (0.7% to 13.0%) per 10 percentage points |

**Table S9. Study-level contributions to all pooled syntheses.**

| **ID** | **Study** | **Comparison** | **Outcome** | **Timepoint** | **N** | **Effect (95% CI)** | **Weight** | **RoB** |
| --- | --- | --- | --- | --- | --- | --- | --- | --- |
| C001 | Dugas et al. (2009) | Oral hydration vs no or minimal fluid | Time-trial/fixed-duration performance | 80 km total | 6 | 2.3% (-1.5% to 6.3%) | 26.6% | Some concerns |
| C002 | Fallowfield et al. (1996) | Oral hydration vs no or minimal fluid | Time-to-exhaustion performance | To exhaustion | 8 | 32.6% (21.7% to 44.4%) | 93.2% | Some concerns |
| C003 | Kay et al. (2003) | Oral hydration vs no or minimal fluid | Time-trial/fixed-duration performance | 60 min | 7 | -3.4% (-13.8% to 8.1%) | 3.6% | Some concerns |
| C005 | McConell et al. (1997) | Oral hydration vs no or minimal fluid | Time-to-exhaustion performance | After 2 h preload | 7 | 91.8% (-9.9% to 308.1%) | 1.2% | Some concerns |
| C006 | Perreault-Brière et al. (2019) | Oral hydration vs no or minimal fluid | Time-trial/fixed-duration performance | 60 min | 9 | 0.3% (-3.2% to 3.9%) | 29.5% | Some concerns |
| C007 | Robinson et al. (1995) | Oral hydration vs no or minimal fluid | Time-trial/fixed-duration performance | 60 min | 8 | -1.7% (-4.5% to 1.2%) | 40.2% | Some concerns |
| C008 | Walsh et al. (1994) | Oral hydration vs no or minimal fluid | Time-to-exhaustion performance | After 60 min preload | 6 | 50.8% (6.6% to 113.2%) | 5.6% | Some concerns |
| C009 | Dugas et al. (2009) | Oral hydration vs no or minimal fluid | Core temperature | Post 80 km | 6 | -0.30 (-0.62 to 0.02) °C | 11.3% | Some concerns |
| C010 | Fallowfield et al. (1996) | Oral hydration vs no or minimal fluid | Core temperature | End exercise | 8 | 0.30 (-0.38 to 0.98) °C | 7.8% | Some concerns |
| C011 | Kay et al. (2003) | Oral hydration vs no or minimal fluid | Core temperature | End exercise | 7 | -0.25 (-0.51 to 0.01) °C | 11.8% | Some concerns |
| C012 | Lambert et al. (2008) | Oral hydration vs no or minimal fluid | Core temperature | End exercise | 20 | -0.30 (-0.52 to -0.08) °C | 12.1% | Some concerns |
| C013 | Montain et al. (1992a) | Oral hydration vs no or minimal fluid | Core temperature | 120 min | 7 | -0.80 (-1.00 to -0.60) °C | 12.3% | Some concerns |
| C014 | Nassis et al. (2002) | Oral hydration vs no or minimal fluid | Core temperature | 90 min | 11 | -0.30 (-0.56 to -0.04) °C | 11.8% | Some concerns |
| C015 | Robinson et al. (1995) | Oral hydration vs no or minimal fluid | Core temperature | End exercise | 8 | -0.50 (-0.89 to -0.11) °C | 10.6% | Some concerns |
| C016 | Walsh et al. (1994) | Oral hydration vs no or minimal fluid | Core temperature | 60 min | 6 | 0.20 (-0.12 to 0.52) °C | 11.3% | Some concerns |
| C017 | Costa et al. (2019) | Oral hydration vs no or minimal fluid | Heart rate | 120 min | 11 | -7.00 (-18.14 to 4.14) beats/min | 5.9% | Some concerns |
| C018 | Fallowfield et al. (1996) | Oral hydration vs no or minimal fluid | Heart rate | End exercise | 8 | 5.00 (-8.58 to 18.58) beats/min | 4.8% | Some concerns |
| C019 | Juett et al. (2024) | Oral hydration vs no or minimal fluid | Heart rate | Mean during exercise | 14 | -5.00 (-10.52 to 0.52) beats/min | 9.5% | Some concerns |
| C020 | Kay et al. (2003) | Oral hydration vs no or minimal fluid | Heart rate | Mean during exercise | 7 | -2.50 (-9.58 to 4.58) beats/min | 8.4% | Some concerns |
| C021 | Lambert et al. (2008) | Oral hydration vs no or minimal fluid | Heart rate | End exercise | 20 | -1.00 (-5.02 to 3.02) beats/min | 10.4% | Some concerns |
| C022 | Maughan et al. (1996) | Oral hydration vs no or minimal fluid | Heart rate | Exhaustion | 12 | -2.00 (-23.20 to 19.20) beats/min | 2.6% | Some concerns |
| C023 | Montain et al. (1992a) | Oral hydration vs no or minimal fluid | Heart rate | 110 min | 7 | -15.00 (-18.39 to -11.61) beats/min | 10.8% | Some concerns |
| C024 | Perreault-Brière et al. (2019) | Oral hydration vs no or minimal fluid | Heart rate | Mean during exercise | 9 | -1.00 (-4.97 to 2.97) beats/min | 10.4% | Some concerns |
| C025 | Robinson et al. (1995) | Oral hydration vs no or minimal fluid | Heart rate | Mean during exercise | 8 | -9.00 (-11.05 to -6.95) beats/min | 11.4% | Some concerns |
| C026 | Sanders et al. (1999) | Oral hydration vs no or minimal fluid | Heart rate | 90 min | 6 | -9.00 (-12.92 to -5.08) beats/min | 10.5% | Some concerns |
| C027 | Walsh et al. (1994) | Oral hydration vs no or minimal fluid | Heart rate | 60 min | 6 | -5.00 (-16.79 to 6.79) beats/min | 5.6% | Some concerns |
| C028 | Costa et al. (2019) | Oral hydration vs no or minimal fluid | Body mass loss (%) | Post exercise | 11 | -2.50 (-2.93 to -2.07) percentage points | 14.0% | Some concerns |
| C030 | Lambert et al. (2008) | Oral hydration vs no or minimal fluid | Body mass loss (%) | Post exercise | 20 | -1.46 (-1.64 to -1.28) percentage points | 14.5% | Some concerns |
| C031 | McConell et al. (1997) | Oral hydration vs no or minimal fluid | Body mass loss (%) | Post 2 h | 7 | -3.10 (-3.30 to -2.90) percentage points | 14.5% | Some concerns |
| C032 | Montain et al. (1992a) | Oral hydration vs no or minimal fluid | Body mass loss (%) | Post 2 h | 7 | -3.00 (-3.39 to -2.61) percentage points | 14.1% | Some concerns |
| C033 | Nassis et al. (2002) | Oral hydration vs no or minimal fluid | Body mass loss (%) | Post 90 min | 11 | -1.62 (-1.96 to -1.29) percentage points | 14.2% | Some concerns |
| C034 | Perreault-Brière et al. (2019) | Oral hydration vs no or minimal fluid | Body mass loss (%) | Post 60 min | 9 | -2.30 (-2.53 to -2.07) percentage points | 14.4% | Some concerns |
| C036 | Costa et al. (2019) | Oral hydration vs no or minimal fluid | Plasma volume change (%) | Post exercise | 11 | 4.80 (1.78 to 7.82) percentage points | 14.1% | Some concerns |
| C037 | Fallowfield et al. (1996) | Oral hydration vs no or minimal fluid | Plasma volume change (%) | Post exercise | 8 | -2.40 (-4.56 to -0.24) percentage points | 16.1% | Some concerns |
| C038 | Maughan et al. (1996) | Oral hydration vs no or minimal fluid | Plasma volume change (%) | Exhaustion | 12 | 3.20 (0.74 to 5.66) percentage points | 15.4% | Some concerns |
| C039 | McConell et al. (1997) | Oral hydration vs no or minimal fluid | Plasma volume change (%) | End 2 h | 7 | 5.00 (1.76 to 8.24) percentage points | 13.6% | Some concerns |
| C041 | Perreault-Brière et al. (2019) | Oral hydration vs no or minimal fluid | Plasma volume change (%) | Post exercise | 9 | 3.00 (-1.77 to 7.77) percentage points | 10.3% | Some concerns |
| C042 | Sanders et al. (1999) | Oral hydration vs no or minimal fluid | Plasma volume change (%) | 90 min | 6 | 3.40 (0.75 to 6.05) percentage points | 15.0% | Some concerns |
| C044 | Costa et al. (2019) | Oral hydration vs no or minimal fluid | Rating of perceived exertion (Borg 6-20) | 120 min | 11 | -1.00 (-2.80 to 0.80) AU | 13.1% | High |
| C045 | Lambert et al. (2008) | Oral hydration vs no or minimal fluid | Rating of perceived exertion (Borg 6-20) | Peak | 20 | 0.00 (-0.76 to 0.76) AU | 41.3% | High |
| C046 | Robinson et al. (1995) | Oral hydration vs no or minimal fluid | Rating of perceived exertion (Borg 6-20) | End exercise | 8 | -0.90 (-1.58 to -0.22) AU | 45.6% | High |
| C048 | Fallowfield et al. (1996) | Oral hydration vs no or minimal fluid | Plasma sodium concentration | Post exercise | 8 | 0.00 (-2.23 to 2.23) mmol/L | 24.3% | Some concerns |
| C049 | Robinson et al. (1995) | Oral hydration vs no or minimal fluid | Plasma sodium concentration | Post exercise | 8 | -4.40 (-5.49 to -3.31) mmol/L | 26.2% | Some concerns |
| C050 | Sanders et al. (1999) | Oral hydration vs no or minimal fluid | Plasma sodium concentration | 90 min | 6 | -4.00 (-5.96 to -2.04) mmol/L | 24.8% | Some concerns |
| C053 | Costa et al. (2019) | Oral hydration vs no or minimal fluid | Serum/plasma osmolality | Post exercise | 11 | -15.00 (-23.19 to -6.81) mOsm/kg | 29.9% | Some concerns |
| C054 | Robinson et al. (1995) | Oral hydration vs no or minimal fluid | Serum/plasma osmolality | Post exercise | 8 | -4.00 (-7.54 to -0.46) mOsm/kg | 36.0% | Some concerns |
| C055 | Dugas et al. (2009) | Oral hydration vs no or minimal fluid | Urine volume (L) | Post exercise | 6 | 0.07 (-0.03 to 0.17) L | 27.5% | Some concerns |
| C056 | McConell et al. (1997) | Oral hydration vs no or minimal fluid | Urine volume (L) | Trial total | 7 | 0.20 (-0.07 to 0.46) L | 5.4% | Some concerns |
| C057 | Perreault-Brière et al. (2019) | Oral hydration vs no or minimal fluid | Urine volume (L) | Post exercise | 9 | -0.01 (-0.10 to 0.07) L | 33.5% | Some concerns |
| C058 | Robinson et al. (1995) | Oral hydration vs no or minimal fluid | Urine volume (L) | Trial total | 8 | 0.09 (0.00 to 0.18) L | 33.6% | Some concerns |
| C060 | Dugas et al. (2009) | Oral hydration vs no or minimal fluid | Whole-body sweat rate | 80 km total | 6 | -0.13 (-0.21 to -0.05) L/h | 25.2% | Some concerns |
| C062 | Kay et al. (2003) | Oral hydration vs no or minimal fluid | Whole-body sweat rate | 60 min | 7 | -0.05 (-0.33 to 0.23) L/h | 6.3% | Some concerns |
| C063 | Lambert et al. (2008) | Oral hydration vs no or minimal fluid | Whole-body sweat rate | 60 min | 20 | 0.03 (-0.10 to 0.16) L/h | 17.6% | Some concerns |
| C064 | Montain et al. (1992a) | Oral hydration vs no or minimal fluid | Whole-body sweat rate | 2 h | 7 | 0.06 (-0.08 to 0.20) L/h | 16.5% | Some concerns |
| C065 | Nassis et al. (2002) | Oral hydration vs no or minimal fluid | Whole-body sweat rate | 90 min | 11 | -0.01 (-0.14 to 0.12) L/h | 18.1% | Some concerns |
| C066 | Robinson et al. (1995) | Oral hydration vs no or minimal fluid | Whole-body sweat rate | 60 min | 8 | -0.04 (-0.30 to 0.22) L/h | 7.0% | Some concerns |
| C067 | Juett et al. (2024) | Oral hydration vs no or minimal fluid | Fluid intake (L) | Trial total | 12 | 1.17 (0.69 to 1.66) L | 11.6% | Some concerns |
| C068 | Kay et al. (2003) | Oral hydration vs no or minimal fluid | Fluid intake (L) | 60 min | 7 | 1.40 (1.14 to 1.66) L | 12.5% | Some concerns |
| C069 | McConell et al. (1997) | Oral hydration vs no or minimal fluid | Fluid intake (L) | 2 h | 7 | 2.32 (2.12 to 2.52) L | 12.7% | Some concerns |
| C070 | Montain et al. (1992a) | Oral hydration vs no or minimal fluid | Fluid intake (L) | 2 h | 7 | 2.41 (2.20 to 2.61) L | 12.7% | Some concerns |
| C071 | Nassis et al. (2002) | Oral hydration vs no or minimal fluid | Fluid intake (L) | 90 min | 11 | 1.27 (1.12 to 1.41) L | 12.8% | Some concerns |
| C072 | Perreault-Brière et al. (2019) | Oral hydration vs no or minimal fluid | Fluid intake (L) | 60 min | 9 | 1.61 (1.52 to 1.69) L | 12.9% | Some concerns |
| C073 | Robinson et al. (1995) | Oral hydration vs no or minimal fluid | Fluid intake (L) | Trial total | 8 | 1.49 (1.22 to 1.76) L | 12.5% | Some concerns |
| C075 | Costa et al. (2019) | Oral hydration vs no or minimal fluid | Body mass change (kg) | Post exercise | 11 | 1.40 (-1.51 to 4.31) kg | 1.4% | Some concerns |
| C076 | Kay et al. (2003) | Oral hydration vs no or minimal fluid | Body mass change (kg) | Post exercise | 7 | 1.47 (1.21 to 1.73) kg | 24.0% | Some concerns |
| C077 | McConell et al. (1997) | Oral hydration vs no or minimal fluid | Body mass change (kg) | Post 2 h | 7 | 1.96 (1.77 to 2.15) kg | 25.5% | Some concerns |
| C079 | Nassis et al. (2002) | Oral hydration vs no or minimal fluid | Body mass change (kg) | Post 90 min | 11 | 1.27 (1.08 to 1.47) kg | 25.4% | Some concerns |
| C080 | Walsh et al. (1994) | Oral hydration vs no or minimal fluid | Body mass change (kg) | After 60 min | 6 | 1.14 (0.87 to 1.41) kg | 23.7% | Some concerns |
| C090 | Nassis et al. (2002) | Oral hydration vs no or minimal fluid | Mean skin temperature | 90 min | 11 | 0.50 (-0.71 to 1.71) °C | 16.6% | Some concerns |
| C091 | Robinson et al. (1995) | Oral hydration vs no or minimal fluid | Mean skin temperature | End exercise | 8 | -0.40 (-1.58 to 0.78) °C | 17.4% | Some concerns |
| C092 | Walsh et al. (1994) | Oral hydration vs no or minimal fluid | Mean skin temperature | 60 min | 6 | 0.60 (0.12 to 1.08) °C | 66.0% | Some concerns |
| C097 | Dugas et al. (2009) | Oral hydration vs no or minimal fluid | Sweat loss | 80 km total | 6 | -0.22 (-0.36 to -0.08) L | 34.4% | Some concerns |
| C098 | McConell et al. (1997) | Oral hydration vs no or minimal fluid | Sweat loss | 2 h | 7 | 0.10 (-0.10 to 0.30) L | 28.1% | Some concerns |
| C099 | Perreault-Brière et al. (2019) | Oral hydration vs no or minimal fluid | Sweat loss | 60 min | 9 | -0.09 (-0.21 to 0.02) L | 37.4% | Some concerns |
| C101 | Dugas et al. (2009) | Oral hydration vs no or minimal fluid | Sweat loss replaced | 80 km total | 6 | 94.60 (85.96 to 103.24) % | 24.2% | Some concerns |
| C102 | McConell et al. (1997) | Oral hydration vs no or minimal fluid | Sweat loss replaced | 2 h | 7 | 113.80 (104.78 to 122.82) % | 24.0% | Some concerns |
| C103 | Montain et al. (1992a) | Oral hydration vs no or minimal fluid | Sweat loss replaced | 2 h | 7 | 80.00 (75.69 to 84.31) % | 26.1% | Some concerns |
| C105 | Dion et al. (2013) | Programmed or individualized vs self-regulated drinking | Time-trial/fixed-duration performance | Half-marathon total | 10 | 0.2% (-5.0% to 5.7%) | 20.0% | Some concerns |
| C106 | Jeker et al. (2022) | Programmed or individualized vs self-regulated drinking | Time-trial/fixed-duration performance | 20 km TT | 8 | 2.2% (-1.7% to 6.3%) | 37.5% | Some concerns |
| C108 | Perreault-Brière et al. (2019) | Programmed or individualized vs self-regulated drinking | Time-trial/fixed-duration performance | 60 min | 9 | -0.3% (-3.9% to 3.4%) | 42.5% | Some concerns |
| C109 | Dion et al. (2013) | Programmed or individualized vs self-regulated drinking | Core temperature | Mean during race | 10 | -0.30 (-0.52 to -0.08) °C | 50.1% | Some concerns |
| C110 | Jeker et al. (2022) | Programmed or individualized vs self-regulated drinking | Core temperature | Post 20 km TT | 8 | -0.20 (-0.48 to 0.08) °C | 32.6% | Some concerns |
| C111 | Lopez et al. (2016) | Programmed or individualized vs self-regulated drinking | Core temperature | Pre-to-post increase | 13 | -0.09 (-0.47 to 0.29) °C | 17.3% | Some concerns |
| C112 | Dion et al. (2013) | Programmed or individualized vs self-regulated drinking | Heart rate | Mean during race | 10 | -4.00 (-9.30 to 1.30) beats/min | 24.2% | Some concerns |
| C113 | Perreault-Brière et al. (2019) | Programmed or individualized vs self-regulated drinking | Heart rate | Mean during exercise | 9 | -3.00 (-5.99 to -0.01) beats/min | 75.8% | Some concerns |
| C114 | Dion et al. (2013) | Programmed or individualized vs self-regulated drinking | Body mass loss (%) | Post exercise | 10 | -1.80 (-2.21 to -1.39) percentage points | 22.1% | Some concerns |
| C115 | Jeker et al. (2022) | Programmed or individualized vs self-regulated drinking | Body mass loss (%) | Post 20 km TT | 8 | -1.00 (-1.66 to -0.34) percentage points | 12.4% | Some concerns |
| C116 | Lopez et al. (2016) | Programmed or individualized vs self-regulated drinking | Body mass loss (%) | Post race | 13 | -1.27 (-1.56 to -0.98) percentage points | 29.2% | Some concerns |
| C117 | Perreault-Brière et al. (2019) | Programmed or individualized vs self-regulated drinking | Body mass loss (%) | Post 60 min | 9 | -1.60 (-1.77 to -1.43) percentage points | 36.3% | Some concerns |
| C119 | Jeker et al. (2022) | Programmed or individualized vs self-regulated drinking | Abdominal discomfort | Mean during exercise | 8 | 0.20 (-0.10 to 0.50) AU | 74.1% | High |
| C120 | Perreault-Brière et al. (2019) | Programmed or individualized vs self-regulated drinking | Abdominal discomfort | Mean during exercise | 9 | 0.30 (-0.21 to 0.81) AU | 25.9% | High |
| C122 | Jeker et al. (2022) | Programmed or individualized vs self-regulated drinking | Urine volume (L) | Trial total | 8 | 0.86 (0.24 to 1.48) L | 42.8% | Some concerns |
| C123 | Perreault-Brière et al. (2019) | Programmed or individualized vs self-regulated drinking | Urine volume (L) | Post exercise | 9 | 0.04 (-0.05 to 0.12) L | 57.2% | Some concerns |
| C124 | Dion et al. (2013) | Programmed or individualized vs self-regulated drinking | Whole-body sweat rate | Half-marathon | 10 | 0.10 (-0.09 to 0.29) L/h | 12.2% | Some concerns |
| C125 | Jeker et al. (2022) | Programmed or individualized vs self-regulated drinking | Whole-body sweat rate | Trial total | 8 | 0.00 (-0.07 to 0.08) L/h | 77.1% | Some concerns |
| C126 | Lopez et al. (2016) | Programmed or individualized vs self-regulated drinking | Whole-body sweat rate | 20 km race | 13 | 0.06 (-0.14 to 0.26) L/h | 10.6% | Some concerns |
| C127 | Dion et al. (2013) | Programmed or individualized vs self-regulated drinking | Fluid intake (L) | Half-marathon total | 10 | 1.48 (1.23 to 1.73) L | 28.3% | Some concerns |
| C128 | Jeker et al. (2022) | Programmed or individualized vs self-regulated drinking | Fluid intake (L) | 5 h + TT total | 8 | 1.55 (0.83 to 2.27) L | 10.8% | Some concerns |
| C129 | Lopez et al. (2016) | Programmed or individualized vs self-regulated drinking | Fluid intake (L) | 20 km total | 13 | 0.95 (0.65 to 1.25) L | 25.9% | Some concerns |
| C130 | Perreault-Brière et al. (2019) | Programmed or individualized vs self-regulated drinking | Fluid intake (L) | 60 min | 9 | 1.04 (0.94 to 1.14) L | 35.0% | Some concerns |
| C135 | Dion et al. (2013) | Programmed or individualized vs self-regulated drinking | Sweat loss | Half-marathon total | 10 | 0.14 (-0.06 to 0.34) L | 35.9% | Some concerns |
| C136 | Jeker et al. (2022) | Programmed or individualized vs self-regulated drinking | Sweat loss | Trial total | 8 | 0.00 (-0.42 to 0.42) L | 14.5% | Some concerns |
| C137 | Perreault-Brière et al. (2019) | Programmed or individualized vs self-regulated drinking | Sweat loss | 60 min | 9 | -0.11 (-0.22 to 0.00) L | 49.6% | Some concerns |
| C141 | Dugas et al. (2009) | Higher vs lower fluid-replacement dose | Time-trial/fixed-duration performance | 80 km total | 6 | 0.6% (-2.8% to 4.1%) | 37.6% | Some concerns |
| C143 | Sage et al. (2026) | Higher vs lower fluid-replacement dose | Time-trial/fixed-duration performance | Time trial | 11 | 9.8% (2.7% to 17.3%) | 25.3% | Some concerns |
| C145 | Dugas et al. (2009) | Higher vs lower fluid-replacement dose | Core temperature | Post 80 km | 6 | -0.30 (-0.67 to 0.07) °C | 14.3% | Some concerns |
| C146 | Sage et al. (2026) | Higher vs lower fluid-replacement dose | Core temperature | End 180 min preload | 11 | -0.30 (-0.47 to -0.13) °C | 85.7% | Some concerns |
| C147 | McConell et al. (1997) | Higher vs lower fluid-replacement dose | Body mass loss (%) | Post 2 h | 7 | -1.70 (-1.90 to -1.50) percentage points | 35.8% | Some concerns |
| C149 | Sage et al. (2026) | Higher vs lower fluid-replacement dose | Body mass loss (%) | Post time trial | 11 | -0.90 (-1.40 to -0.40) percentage points | 30.3% | Some concerns |
| C150 | McConell et al. (1997) | Higher vs lower fluid-replacement dose | Plasma volume change (%) | End 2 h | 7 | 5.40 (2.51 to 8.29) percentage points | 43.8% | Some concerns |
| C151 | Sage et al. (2026) | Higher vs lower fluid-replacement dose | Plasma volume change (%) | End 180 min preload | 9 | 5.00 (2.00 to 8.00) percentage points | 56.2% | Some concerns |
| C152 | Dugas et al. (2009) | Higher vs lower fluid-replacement dose | Urine volume (L) | Post exercise | 6 | 0.10 (0.01 to 0.19) L | 70.3% | Some concerns |
| C153 | McConell et al. (1997) | Higher vs lower fluid-replacement dose | Urine volume (L) | Trial total | 7 | 0.17 (-0.09 to 0.42) L | 9.3% | Some concerns |
| C154 | Sage et al. (2026) | Higher vs lower fluid-replacement dose | Urine volume (L) | Trial total | 11 | 0.20 (0.02 to 0.37) L | 20.3% | Some concerns |
| C156 | McConell et al. (1997) | Higher vs lower fluid-replacement dose | Fluid intake (L) | 2 h | 7 | 1.16 (0.99 to 1.33) L | 33.5% | Some concerns |
| C157 | Sage et al. (2026) | Higher vs lower fluid-replacement dose | Fluid intake (L) | Time trial | 11 | -0.11 (-0.40 to 0.18) L | 32.4% | Some concerns |
| C158 | Dugas et al. (2009) | Higher vs lower fluid-replacement dose | Fluid intake rate (L/h) | 80 km total | 6 | 0.46 (0.32 to 0.60) L/h | 50.6% | Some concerns |
| C159 | Sage et al. (2026) | Higher vs lower fluid-replacement dose | Fluid intake rate (L/h) | 180 min preload | 11 | 0.28 (0.14 to 0.42) L/h | 49.4% | Some concerns |
| C161 | Dugas et al. (2009) | Higher vs lower fluid-replacement dose | Sweat loss | 80 km total | 6 | -0.35 (-0.52 to -0.18) L | 51.6% | Some concerns |
| C162 | McConell et al. (1997) | Higher vs lower fluid-replacement dose | Sweat loss | 2 h | 7 | -0.07 (-0.27 to 0.13) L | 48.4% | Some concerns |
| C163 | Dugas et al. (2009) | Higher vs lower fluid-replacement dose | Sweat loss replaced | 80 km total | 6 | 34.70 (27.17 to 42.23) % | 33.3% | Some concerns |
| C164 | McConell et al. (1997) | Higher vs lower fluid-replacement dose | Sweat loss replaced | 2 h | 7 | 58.50 (50.67 to 66.33) % | 33.2% | Some concerns |
| C170 | Sanders et al. (1999) | Higher vs lower sodium/electrolyte dose | Plasma volume change (%) | 90 min | 6 | 0.70 (-1.95 to 3.35) percentage points | 16.7% | Some concerns |
| C171 | Wijering et al. (2023) | Higher vs lower sodium/electrolyte dose | Plasma volume change (%) | Baseline to final | 11 | 2.00 (0.50 to 3.20) percentage points | 83.3% | Some concerns |
| C186 | Gonzalez-Alonso et al. (1995) | Oral hydration vs no or minimal fluid | Body mass loss (%) | Post 2 h | 7 | -4.46 (-4.78 to -4.14) percentage points | 14.3% | Some concerns |
| C187 | Gonzalez-Alonso et al. (1995) | Oral hydration vs no or minimal fluid | Core temperature | 120 min | 7 | -1.30 (-1.64 to -0.96) °C | 11.1% | Some concerns |
| C188 | Gonzalez-Alonso et al. (1995) | Oral hydration vs no or minimal fluid | Fluid intake (L) | 2 h | 7 | 3.40 (3.13 to 3.67) L | 12.5% | Some concerns |
| C189 | Gonzalez-Alonso et al. (1995) | Oral hydration vs no or minimal fluid | Heart rate | 110-120 min | 7 | -19.00 (-24.19 to -13.81) beats/min | 9.7% | Some concerns |
| C190 | Gonzalez-Alonso et al. (1995) | Oral hydration vs no or minimal fluid | Plasma sodium concentration | 120 min | 7 | -9.00 (-10.96 to -7.04) mmol/L | 24.8% | Some concerns |
| C191 | Gonzalez-Alonso et al. (1995) | Oral hydration vs no or minimal fluid | Plasma volume change (%) | 120 min | 7 | 6.40 (3.99 to 8.81) percentage points | 15.5% | Some concerns |
| C192 | Gonzalez-Alonso et al. (1995) | Oral hydration vs no or minimal fluid | Serum/plasma osmolality | 120 min | 7 | -20.00 (-25.19 to -14.81) mOsm/kg | 34.1% | Some concerns |
| C193 | Gonzalez-Alonso et al. (1995) | Oral hydration vs no or minimal fluid | Sweat loss replaced | 2 h | 7 | 92.40 (87.14 to 97.66) % | 25.8% | Some concerns |
| C194 | Gonzalez-Alonso et al. (1995) | Oral hydration vs no or minimal fluid | Whole-body sweat rate | 2 h | 7 | 0.13 (-0.09 to 0.35) L/h | 9.3% | Some concerns |
| C196 | Backx et al. (2003) | Higher vs lower fluid-replacement dose | Body mass loss (%) | Post exercise | 8 | -0.64 (-0.96 to -0.31) percentage points | 33.9% | Some concerns |
| C197 | Backx et al. (2003) | Higher vs lower fluid-replacement dose | Time-trial/fixed-duration performance | 60 min | 8 | 0.2% (-3.3% to 3.8%) | 37.1% | Some concerns |
| C198 | Backx et al. (2003) | Higher vs lower fluid-replacement dose | Fluid intake (L) | 60 min | 8 | 0.45 (0.42 to 0.49) L | 34.1% | Some concerns |
| C200 | Backx et al. (2003) | Higher vs lower fluid-replacement dose | Sweat loss replaced | 60 min | 8 | 24.00 (16.70 to 31.30) % | 33.4% | Some concerns |

The 137 rows include both main and supporting models; outputs/pooled_results.csv identifies model role. Within-study dependence is resolved before assigning random-effects weights. Each study contributes at most once to a given comparison/construct. Baseline-only values, capped-duration performance and unverified Montain (1992b) values have no quantitative weight.

**Table S10. Supporting pooled outcomes outside the critical/important efficacy summaries.**

| **Comparison** | **Outcome** | **Studies (N)** | **Effect (95% CI)** | **95% prediction interval** | **I² (%)** |
| --- | --- | --- | --- | --- | --- |
| Oral hydration vs no or minimal fluid | Body mass change (kg) | 5 (42) | 1.47 (0.97 to 1.96) kg | 0.24 to 2.69 | 88.4 |
| Oral hydration vs no or minimal fluid | Fluid intake (L) | 8 (68) | 1.89 (1.25 to 2.53) L | -0.07 to 3.84 | 97.6 |
| Oral hydration vs no or minimal fluid | Mean skin temperature | 3 (25) | 0.41 (-0.73 to 1.55) °C | -3.95 to 4.77 | 16.2 |
| Oral hydration vs no or minimal fluid | Sweat loss | 3 (22) | -0.08 (-0.47 to 0.30) L | -2.06 to 1.90 | 70.5 |
| Oral hydration vs no or minimal fluid | Sweat loss replaced | 4 (27) | 94.83 (72.69 to 116.96) % | 29.88 to 159.77 | 93.9 |
| Oral hydration vs no or minimal fluid | Whole-body sweat rate | 7 (66) | -0.01 (-0.11 to 0.08) L/h | -0.21 to 0.18 | 43.1 |
| Programmed or individualized vs self-regulated drinking | Fluid intake (L) | 4 (40) | 1.20 (0.74 to 1.65) L | 0.00 to 2.39 | 75.5 |
| Programmed or individualized vs self-regulated drinking | Sweat loss | 3 (27) | -0.00 (-0.41 to 0.40) L | -1.95 to 1.95 | 57.7 |
| Programmed or individualized vs self-regulated drinking | Whole-body sweat rate | 3 (31) | 0.02 (-0.12 to 0.16) L/h | -0.40 to 0.44 | 0.0 |
| Higher vs lower fluid-replacement dose | Fluid intake (L) | 3 (26) | 0.51 (-1.06 to 2.08) L | -8.66 to 9.67 | 97.5 |
| Higher vs lower fluid-replacement dose | Fluid intake rate (L/h) | 2 (17) | 0.37 (-0.76 to 1.50) L/h | Not estimable (k<3) | 67.5 |
| Higher vs lower fluid-replacement dose | Sweat loss | 2 (13) | -0.21 (-1.99 to 1.56) L | Not estimable (k<3) | 77.6 |
| Higher vs lower fluid-replacement dose | Sweat loss replaced | 3 (21) | 39.03 (-4.82 to 82.87) % | -215.18 to 293.23 | 95.1 |

These 13 models describe fluid provision, balance and other supporting measures. They are not additional graded efficacy claims or independent confirmations of benefit. Measurement periods can differ across studies and are listed in Table S9. A statistically non-null manipulation measure does not establish improved performance or safety.

**Figure S1. Supporting hydration physiology.**

Oral hydration versus no or minimal fluid: A, plasma sodium concentration; B, serum/plasma osmolality; C, urine volume. Mean differences are hydration minus comparator. Sodium and osmolality are context-dependent biomarkers; a lower value is not automatically a clinical benefit. No baseline-only sodium value is included.

**Figure S2. Programmed or individualized versus self-regulated drinking.**

A, time-trial/fixed-duration performance; B, body-mass loss; C, core temperature; D, heart rate. Performance ratios above 1 favor the first-named strategy; negative mean differences indicate a smaller deficit or lower physiological value. The gastrointestinal-temperature change score from Lopez is identified in the source table and examined separately in sensitivity analysis.

**Figure S3. Higher versus lower fluid-replacement dose.**

A, time-trial/fixed-duration performance; B, body-mass loss; C, core temperature; D, plasma-volume change. Sage’s end-preload temperature and plasma-volume contrasts are included as the latest available measurements for those outcomes; blood outcomes use n=9. No unverified Montain (1992b) value is included.

**Figure S4. Higher versus lower sodium/electrolyte dose under matched fluid provision.**

A, pooled plasma-volume change; B, single-study plasma sodium concentration; C, single-study sodium change; D, single-study body-mass loss. Panels B–D are direct evidence, not meta-analyses. The capped duration in Wijering is not classified as time-trial performance. No prediction interval is reported for the two-study model.

**Figure S5. Exploratory within-study fluid-dose slopes, reported separately by performance construct.**

Slopes are direction-harmonized log-ratio changes per 10 percentage points of target sweat-loss replacement, back-transformed to percentages. Within-study generalized least squares incorporates shared-reference covariance with r=0.50. Dugas assessed a time trial and McConell time to exhaustion; no cross-construct pooled slope, nonlinear model or optimal-dose estimate is presented.

**Figure S6. Leave-one-out sensitivity analyses for selected hydration outcomes.**

Each point omits the named study and recalculates the corresponding random-effects estimate and modified Hartung–Knapp confidence interval. Performance is expressed as percentage difference; temperature and heart rate retain their original units. The full sensitivity dataset also records cases with fewer than two remaining studies, for which no pooled estimate is reported.

**Figure S7. Exploratory funnel plot for the hydration heart-rate synthesis.**

Heart rate was the only synthesis with at least ten independent studies (k=12). The vertical line is the pooled estimate and dashed lines are pseudo 95% limits. Egger’s intercept test gave p=0.413. High heterogeneity and limited power prevent exclusion of small-study or reporting effects; the plot is not evidence that publication bias is absent.
